# Linkage mapping of root shape traits in two carrot populations

**DOI:** 10.1093/g3journal/jkae041

**Published:** 2024-02-27

**Authors:** Andrey Vega, Scott H Brainard, Irwin L Goldman

**Affiliations:** Department of Plant and Agroecosystem Sciences, University of Wisconsin-Madison, Madison, WI 53706, USA; Department of Plant and Agroecosystem Sciences, University of Wisconsin-Madison, Madison, WI 53706, USA; Department of Plant and Agroecosystem Sciences, University of Wisconsin-Madison, Madison, WI 53706, USA

**Keywords:** genetic mapping, *Daucus carota* var sativus, OFP-TRM and IQD plant regulon

## Abstract

This study investigated the genetic basis of carrot root shape traits using composite interval mapping in two biparental populations (*n* = 119 and *n* = 128). The roots of carrot F_2:3_ progenies were grown over 2 years and analyzed using a digital imaging pipeline to extract root phenotypes that compose market class. Broad-sense heritability on an entry-mean basis ranged from 0.46 to 0.80 for root traits. Reproducible quantitative trait loci (QTL) were identified on chromosomes 2 and 6 on both populations. Colocalization of QTLs for phenotypically correlated root traits was also observed and coincided with previously identified QTLs in published association and linkage mapping studies. Individual QTLs explained between 14 and 27% of total phenotypic variance across traits, while four QTLs for length-to-width ratio collectively accounted for up to 73% of variation. Predicted genes associated with the *OFP-TRM* (OVATE Family Proteins—TONNEAU1 Recruiting Motif) and *IQD* (IQ67 domain) pathway were identified within QTL support intervals. This observation raises the possibility of extending the current regulon model of fruit shape to include carrot storage roots. Nevertheless, the precise molecular mechanisms through which this pathway operates in roots characterized by secondary growth originating from cambium layers remain unknown.

## Introduction

Carrot (*Daucus carota* var. *sativus*, *2n = 2x =* 18) is a biennial vegetable crop known for its diverse root shapes (Rubatzky *et al*. 1999; Simon 2021). The total national carrot production in the United States was valued at $1.2 billion USD (about $4 per person in the United States) in 2022 [United States Department of Agriculture—National Agricultural Statistics Service (USDA-NASS 2023)]. Carrots are commercialized in market classes which are primarily determined by root shape and end-use. A carrot market class is defined as a group of carrot cultivars that share similar root shape phenotypes and are grouped together to facilitate crop breeding and trade. In Europe, the classification of carrots by shape traces back to at least the 1,600 s (Banga 1957, 1963a, 1963b) and in North America, by the 1940s, the USDA had already established the practice of categorizing carrot cultivars into market classes using descriptions of standard root shapes (Magruder *et al*. 1940).

While there are 10–15 recognized carrot market classes (Geoffriau and Simon 2020), over 80% of released carrot cultivars in the last 85 years have been classified into only four market classes, Imperator, Nantes, Chantenay, and Danvers, according to *HortScience* Vegetable Cultivar Descriptions for North America (Mou. 2022). This emphasizes the role of market classes in carrot breeding and economics and the prevalence of certain shape types that are tied to a specific end use. Imperator, for example, is the most used market class for baby-cut carrots in North America (Lucier and Lin 2007). Root shape traits including length, width, and curvature of the shoulders and tip play a crucial role in categorizing carrot cultivars into market classes. While traditionally assessed subjectively, these traits are now analyzed using digital imaging pipelines (Turner *et al*. 2018; Brainard *et al*. 2021, Vega and Goldman 2023). Understanding the genetic basis of root traits composing market class is essential for carrot improvement efforts. This is because carrot breeding often occurs within market classes as interclass crosses require lengthy breeding cycles to regain a desired shape adding to challenge of selecting for the targeted traits. Alternatively, choosing to breed using only plants within a market class to circumvent this problem may limit the availability of germplasm that is otherwise available through interclass breeding.

Over the course of domestication, selective breeding played a role in shaping the array of root shapes observed in the collection of carrot varieties (Wu *et al*. 2018; Ellison 2019; Geoffriau and Simon 2020). The ability to form a storage root was key in the transition from the wild (*D. carota* var *carota*) to the cultivated carrot (*D. carota* var. *sativus*). The literature on genetic control of carrot root traits suggests two main findings: first, market class is composed of several traits and each trait is likely controlled by multiple genes (polygenic inheritance), and second, chromosome 2 seems to be a key region associated with both the domestication syndrome and the ability of cultivated carrots to develop swollen roots.

For example, Macko-Podgórni *et al*. (2017) identified a polymorphism with signatures for selection on chromosome 2 which distinguished between wild and cultivated carrot accessions. The proposed gene, *DcAHLc1*, belongs to the *AT-hook* nuclear motif of plant regulatory genes, responsible for root tissue patterning. Similarly, using an image analysis pipeline to study root morphology, Turner *et al*. (2018) found evidence of colocalization of QTLs in chromosomes 1, 2, and 7 for correlated carrot root traits, suggesting these traits may be controlled by genetic linkage and quantitative inheritance. Furthermore, Brainard *et al*. (2022) found that phenotypic determinants of market class in carrot are under additive but highly polygenic genetic control. The authors also identified QTLs for four morphological traits that compose root market class in carrot. This included a significant SNP on chromosome 2 associated with root fill, defined as the degree to which a carrot maintains its full width along its length. Their results also indicate the presence of an *OFP8-like* transcription factor less than 40 kb of a significant QTL identified for maximum width on chromosome 3. *OFP8-like* belongs to the *OFP-TRM* (OVATE Family Protein—TONNEAU1 Recruiting Motif) and *IQD* (IQ67 domain) pathway which contain conserved domains involved in regulating biological shape by modulating patterns of cell division in plants.

The plant-specific *OFP-TRM* and *IQD* regulatory pathway is implicated in shape patterning and is well-studied in various plant organs including fruit, leaves, stems, and tubers (van der Knaap *et al*. 2014; Pan *et al*. 2017; Li *et al*. 2023) However, the understanding of the role that this plant organ shape regulon plays in true roots, including carrots, remains limited. To the best of our knowledge, the only study linking the *OFP-TRM* and *IQD* regulon to root shape control is in radish (*Raphanus sativus*) (Wang *et al*. 2020).

These pathways, conserved across plant species of economic and research importance, determine plant organ shape by regulating cell division patterns and integrating external cues (Bürstenbinder *et al*. 2017; Schaefer *et al*. 2017; Zhao *et al*. 2018). Interactions within the *OFP-TRM* and *IQD* pathway also influence protein complex localization, microtubule organization, and cell division patterns which determinate plant organ shape (Lazzaro *et al*. 2018; Yang *et al*. 2020). Research suggests the involvement of the *OFP-TRM* and *IQD* pathways in phytohormone biosynthesis and signaling, microtubule reorganization, and protein interactions (van der Knaap and Østergaard 2018; Zhang *et al*. 2022). The study of the plant-specific *OFP-TRM* and *IQD* regulatory pathways in carrot genetics may help explain the diversity of root shapes that current genetic models don't entirely describe.

In this study, we conducted linkage mapping of two carrot populations to explore the genetic basis of root traits associated with market class. Our objective was to identify loci controlling root shape differences, describe the genetic basis of root shape traits, and investigate whether members of the *OFP-TRM* and *IQD* regulon overlapped with root shape QTLs. Understanding the genetic architecture of root traits can inform breeding decisions and open new opportunities for expansion beyond the carrot market classes available today.

## Materials and methods

### Plant materials

**Fig. 1.**
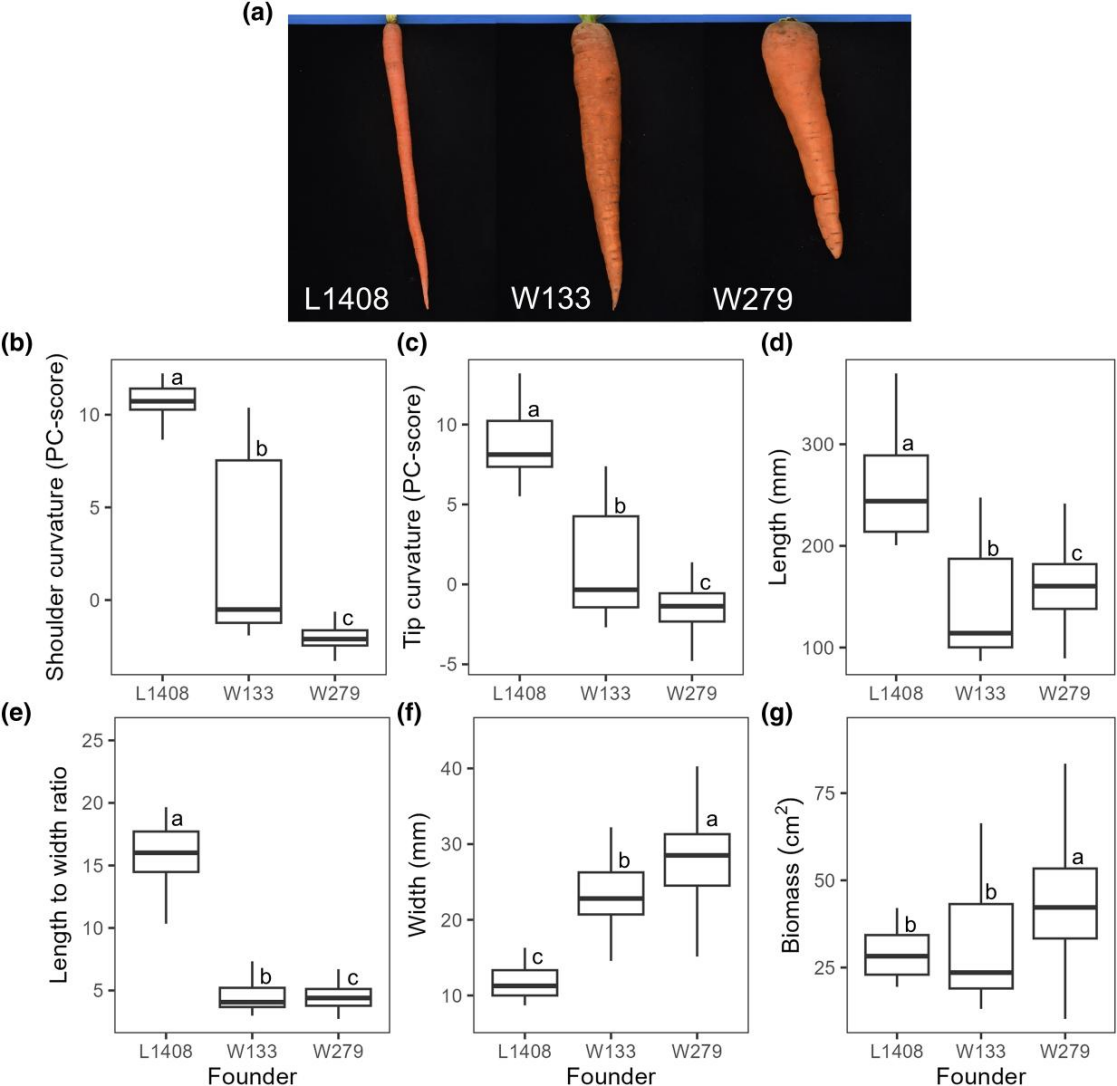
Photograph of carrot (*D. carota* var *sativus*) founders “L1408”, “W133”, and “W279”. a) Boxplots displaying differences in carrot root shape traits among founders: b) shoulder curvature, c) tip curvature, d) length, e) length-to-width ratio, f) width, and g) biomass. Significance of mean differences is indicated by distinct lowercase letters within each boxplot (α = 0.05). Multiple comparisons were corrected using the Šidák correction.

Two F_2:3_ carrot (*D. carota* var. *sativus*) mapping populations, L1408 × W133 and L1408 × W279 were derived from multiple plants of male-fertile founders “L1408”, “W133”, and “W279” (Fig. 1a). Founder “L1408” is a long Imperator type developed by the USDA Vegetable Crops Research Unit. “W133” is a medium-length Danvers type, with a tapering root and acuminate tip and “W279” is a bulkier wedge-shaped Chantenay type, both developed by the University of Wisconsin-Madison (Goldman 1996). Seed of each founder was grown in the field in 2017, harvested 100–120 days after planting and vernalized for at least 6 weeks at 5°C. Flowering-competent roots of each founder were planted in pots at the University of Wisconsin-Madison Walnut St. Greenhouse and kept at 22 ± 2°C, 42 ± 8% relative humidity, and 16 h photoperiod. All crosses were performed starting 5–8 weeks after potting using pollination cages for isolation (Rubatzky *et al*. 1999) and blue bottle fly pollinators (*Calliphora vomitoria,* sourced from Forked Tree Ranch, Port Hill, Idaho).

In the L1408 × W133 population, two F_1_ plants (Supplementary Fig. 1) derived from the cross “L1408” (♀) × “W133” (♂) were grown in the field (2018), vernalized and self-pollinated in the greenhouse to obtain F_2_ progeny. Individual F_2_ plants were field grown from seed the following year (2019), vernalized and self-pollinated, resulting in 119 F_2:3_ families. Similarly, in the L1408 × W279 population two plants of each “L1408” and “W279” founder were cross pollinated in pairs resulting in two reciprocal crosses. Two F_1_ plants were obtained from the L1408 (♀) × W279 (♂) cross and one F_1_ plant from the W279 (♀) × L1408 (♂) cross (Supplementary Fig. 1). All three F_1_ plants were grown in the field in 2018, and individually self-pollinated in the greenhouse to obtain F_2_ progeny as described previously. Each F_2_ plant was grown in the field from seed the following year (2019). Roots were vernalized as described previously and individually self-pollinated resulting in 128 distinct F_2:3_ families.

The root shape in the F_1_ generation of both populations displayed consistent uniformity (Supplementary Fig. 1), despite both mapping populations being derived from multiple founders and F_1_ plants.

### Field-based experimental design

All F_2:3_ progenies from both populations were grown in field experiments at Jack's Pride Farms, Randolph, Wisconsin, United States in the years 2020 and 2021. The type of soil at the experimental site is classified as a Houghton Muck (Typic Haplosaprists) with weak, medium granular structure and with an organic matter content of 20% (Colquhoun *et al*. 2019; USDA, National Cooperative Soil Survey 2021). This type of soil is commonly used in commercial carrot production in Wisconsin. All experiments were planted in a randomized complete block design with one genotype replication per block in each of two blocks. Experimental units of 1-m rows were randomized within each block. Carrot seed was hand planted in raised beds 1.8-m-wide (center to center) and 0.40-m-high at 5 cm spacing between plants and 37.5 cm between rows. Experiments were planted on 2020 April 26 and 2021 May 10 and harvested 2020 August 19 and 2021 August 24. A subsample of 10, or all if less than 10 roots were available, were harvested from the center of the row and stored at 5°C until phenotyping.

### Phenotyping

Phenotyping was conducted following the digital imaging procedure established by Brainard *et al*. (2021). The phenotyping process is delineated and visualized in Supplementary Fig. 2. Every F_2:3_ family within each of two mapping populations was cultivated in the years 2020 and 2021. Each F_2:3_ family had one experimental row, replicated in two blocks each year. We sampled 10 roots per row, or all available if less than 10. In total, the number of roots phenotyped amounted to 8,841.

All measured traits were estimated from root straight masks derived from digital images (Supplementary Fig. 2). Root length (mm) was defined as the distance between the center of the root crown and the root tip. Maximum width (mm) was measured as the widest diameter along the root and was only used to estimate length-to-width ratio. Width (mm) was defined as the diameter of the root at the 50th percentile of its length (mm). Length-to-width ratio was calculated as the ratio of root length to maximum width. Biomass was estimated as the 2D area of the straight mask (mm^2^), also referred to as digital biomass in the digital phenotyping literature. Two additional traits, namely shoulder curvature and tip curvature, were derived by performing principal component analysis (PCA) on contour values at the first 50 pixels and last 50 pixels of each root's straight mask, respectively (Brainard *et al*. 2021). To capture size-independent variation in the PCA-derived traits, straight masks were normalized. To visualize the phenotypic variables of length-to-width ratio, shoulder curvature, and tip curvature, Supplementary Fig. 3 presents roots at the 1st and 99th percentiles of their respective distributions. This illustration is informative given that length-to-width ratio is a proportion of two linear measurements (length and maximum width), while shoulder and tip curvature derive from principal component scores.

### Genotyping

To infer F_2:3_ genotypes, ∼10 F_2:3_ seeds were planted in conical tubes filled with Pro-Mix High Porosity media (Premier Tech, Quakertown, PA, USA) at the University of Wisconsin-Madison Walnut St. Greenhouses in December of 2021. Plants were maintained at 22 ± 2°C and 42 ± 8 relative humidity with a 16 h photoperiod. At 4–5 weeks after planting, 1 cm^2^ of leaf tissue was sampled for each of the ∼10 plants per F_2:3_ family and bulked. Leaf tissue was stored at −80°C for at least 72 h and lyophilized. Lyophilized tissue was macerated, mixed, and 10–50 mg of each bulk sample was transferred to Collection Microtube plates (Giagen, Germantown, MD, USA). Heterozygous genotypes in the F_2_ generation are expected to segregate in a 1:2:1 ratio after one round of inbreeding (F_2:3_ generation). Because only two genotypes are present in each biparental population, the expected allele frequency for heterozygote genotypes in the F_2:3_ generation is 1:1, which allows for accurate identification of heterozygous F_2:3_ genotypes given sufficient read depth.

Plates were submitted for DNA extraction and genotyping by sequencing (GBS) to the University of Wisconsin-Madison Biotechnology Center. Genomic DNA extraction was completed using the QIAGEN DNeasy mericon 96 HT kit and the automated extraction robot QIACube HT (Qiagen, Germantown, MD, USA). Quantification of DNA was performed using the Quant-iT PicoGreen dsDNA kit (Life Technologies, Grand Islan, NY, USA). GBS libraries were prepared following Elshire *et al*. (2011). Restriction enzyme *ApeKI* was used to digest DNA followed by annealing of sample-specific barcodes and Illumina adapters. Multiplexed samples were sequenced using an Illumina NovaSeq 6000 sequencer. On average, 8.5 million reads were obtained per sample. Discovery of single nucleotide polymorphisms (SNPs) was performed by the Bioinformatics Resource Core (https://bioinformatics.biotech.wisc.edu/) using Tassel GBS Version 2 (Glaubitz *et al*. 2014) and version 3 of the carrot reference genome (Coe *et al*. 2023). In populations L1408 × W133 and L1408 × W279, approximately 280,000 unfiltered variants were detected. Initial marker filtering was performed using bcftools (Li, 2011). Insertion deletion markers (indels) were removed and only biallelic SNPs with a 95th percentile of read depths and genotype quality scores ≥ 20 and minor allele frequencies > 0.05 were retained, resulting in 15,078 and 7,275 markers for populations L1408 × W133 and L1408 × W279, respectively.

### Linkage map construction

Additional marker filtering and linkage map construction were performed for each population individually using custom R scripts (R Core Team 2022) and the R package MapRtools (v. 0.30; Endelman 2023). R code and data are available in the Zenodo repository (10.5281/zenodo.10023295). Because a high proportion of heterozygous SNP markers were identified and coincided with highly repetitive regions, markers and F_2:3_ individuals with heterozygote genotype frequencies outside the range of 0.10–0.90 and ≥ 10% missing data were removed. Population L1408 × W279, was derived from two plants of each founder (“L1408” and “W279”). Both founder genotypes showed unexpected high genetic heterogeneity (Supplementary Table 1). As a result, only markers shared by all L1408 × W279 founders were kept, leading to a reduced number of markers available for constructing the L1408 × W279 linkage map.

Markers for all progeny in each population were recoded (phased) according to the founder genotypes for each population. The “L1408” allele was designated as the reference “A” allele, while the alternative “B” allele originated from either the founder “W133” or “W279” accordingly. “A” and “B” denote the two homozygous states and “H” the heterozygote. In the founders, only homozygous markers (A × B and B × A types; Braun *et al*. 2017) were retained for initial genetic map construction resulting in 4,734 and 543 markers for populations L1408 × W133 and L1408 × W279, respectively. Additional filtering and marker binning at a threshold of *r*^2^ = 0.99 using the LDbin function from MapRtools resulted in 2,367 and 361 marker bins for populations L1408 × W133 and L1408 × W279, respectively. Nine linkage groups corresponding to the nine carrot chromosomes were formed at a logarithm of odds (LOD) threshold of 17 for L1408 × W133 and 25 for L1408 × W279. Linkage groups were each trimmed individually and for each population using MapRtools functions LG and plot_genofreq. Resulting markers after linkage group trimming were ordered according to version 3 of the carrot reference genome (Coe *et al*. 2023). Map distances were estimated using the Kosambi mapping function (Kosambi 1943) and 19-point multiple regression using the function MapRtools::genetic_map. Composite interval mapping (CIM) was conducted using Haley-Knott regression, a 10 cM window size, and one to three marker covariates under a single QTL model using the R/qtl cim function. A 1.5-LOD support interval was estimated for identified QTLs using the functions stepwise and lodint (Broman 2023). Genome-wide linkage disequilibrium (LD) decay was estimated and plotted using the MapRTools function plot_LD. Only markers with homozygous states in the founders (A × B and B × A types) were used to construct the genetic maps. However, to fill gaps in the linkage map for chromosomes 3, 8, and 9 of the L1408 × W279 population, we incorporated an additional 18 heterozygous markers (Supplementary Table 2).

### Statistical analysis

Phenotypic data were analyzed using custom R scripts and the function lmer from the R package lme4 (Bates *et al*. 2015) in a two-stage analysis approach (Piepho *et al* 2012). In Stage 1, each genotype was represented by the best linear unbiased estimate (BLUE) computed across years using the following model:


$$Y_{ijk}=\;g_{i}+y_{j}+b_{k(j)}+gy_{ij}+\varepsilon_{ijk},$$

where, $Y_{ijk}$ represents the phenotypic response associated with root shape, $g_{i}$ is the *i*th genotype, $y_{j}$ is the *j*th year, $b_{k(j)}$ is the *k*th block nested within the *j*th year, $gy_{ij}$ is the interaction of the *i*th genotype and the *j*th year, and $\varepsilon_{ijk}$ are the residuals, with $\varepsilon_{ijk}∼\;iid,\;N(0,\sigma_{\varepsilon }^{2})$. The same model with all terms fit as random effects, was used to estimate broad-sense heritability (*H*^2^) on an entry-mean basis from Stage 1 variance components:


(1) $$H^{2}=\frac{\sigma_{G}^{2}}{\sigma_{G}^{2}+\mathrm{PEV}}.\;$$

In equation 1, $\sigma_{G}^{2}$ is the variance associated with genotypes and PEV is the prediction error variance. PEV is given by:


(2) $$\mathrm{PEV}=\frac{\sigma_{Gy}^{2}}{y}+\frac{\sigma_{\varepsilon }^{2}}{ry}.$$

In equation 2, $\sigma_{Gy}^{2}$ is the variance component associated with genotype by year interactions, $\sigma_{\varepsilon }^{2}$ is the residual variance, *y* is the number of years (y=2), and *r* is the number of replicates (r=2). The Stage 1 BLUEs were used as the response variable for linkage mapping in Stage 2.

Each phenotypic trait was fit independently. Across experiments, any phenotypic BLUE outside 3 times the standard deviation above or below the mean was removed as an outlier. Multiple means comparison between the founder phenotypes and effects of allele substitution were performed using the functions emmeans and cld from the emmeans and multicomp R packages (Lenth 2020).

## Candidate genes

Candidate genes in the QTL intervals were identified using BLAST search. To address challenges associated with slow LD decay and large LD blocks impacting QTL size estimation, we targeted homolog genes in the *OFP-TRM* and *IQD* regulon, recognized for their role in shaping plant organs (Li *et al*. 2023). Amino acid sequences of 34 *IQDs*, 27 *OFPs*, and 26 *TRMs* genes involved in the control of fruit shape in tomato (*Solanum lycopersicum*) were obtained from the Solgenomics database (https://solgenomics.net/search/locus). To identify homologs of *IQDs*, *OFPs*, and *TRMs* in carrot, a protein BLAST search was conducted using the NCBI database. Carrot candidate gene information was obtained from the NCBI gene database (https://www.ncbi.nlm.nih.gov/gene). To better characterize predicted genes and infer homology between specific sequences in chromosomal regions encompassing QTL intervals, multiple sequence alignment was performed using Clustal Omega (https://wwwdev.ebi.ac.uk/Tools/jdispatcher/msa). This alignment included known regulators of shape from tomato and carrot candidate genes within a 2 Mb interval of QTL peaks. Motif alignment was conducted using MAST (Bailey and Gribskov 1998) from the MEME suite (https://meme-suite.org/meme/doc/mast.html) to describe four previously uncharacterized predicted carrot genes, DCAR_027681, DCAR_017186, DCAR_21448, and DCAR_008585 (Supplementary File 1). Amino acid sequences of carrot and tomato *IQDs*, *OFPs*, and *TRMs* are available (Supplementary File 2).

## Results

### Phenotypic description

To generate two carrot populations segregating for root shape, the long Imperator type “L1408” was crossed with the bulkier Danvers type “W133” and the Chantenay type “W279”. Root shape trait distributions in both populations showed that the founders were primarily located at the distribution extremes (Supplementary Figs. 4 and 5). Significant differences among all founders were observed in root width, shoulder and tip curvature as well as length. However, no significant differences were found between “W133” and “W279” in length-to-width ratio and biomass (Fig. 1). Generally, “L1408” and “W279” exhibited pronounced phenotypic differences, while “W133” showed intermediate phenotypes across all measured traits, except for length.

The root shape in the F_1_ generation of both populations displayed phenotypic uniformity (Supplementary Fig. 1). This supports our assumption that QTLs for shape traits in these populations carries just two alleles per loci, one from each founder, despite both mapping populations were derived from multiple founders and F_1_ plants, and unexpected genetic heterogeneity was observed for all founders (Supplementary Fig. 1 and Supplementary Table 1).

#### Phenotypic correlations

**Fig. 2.**
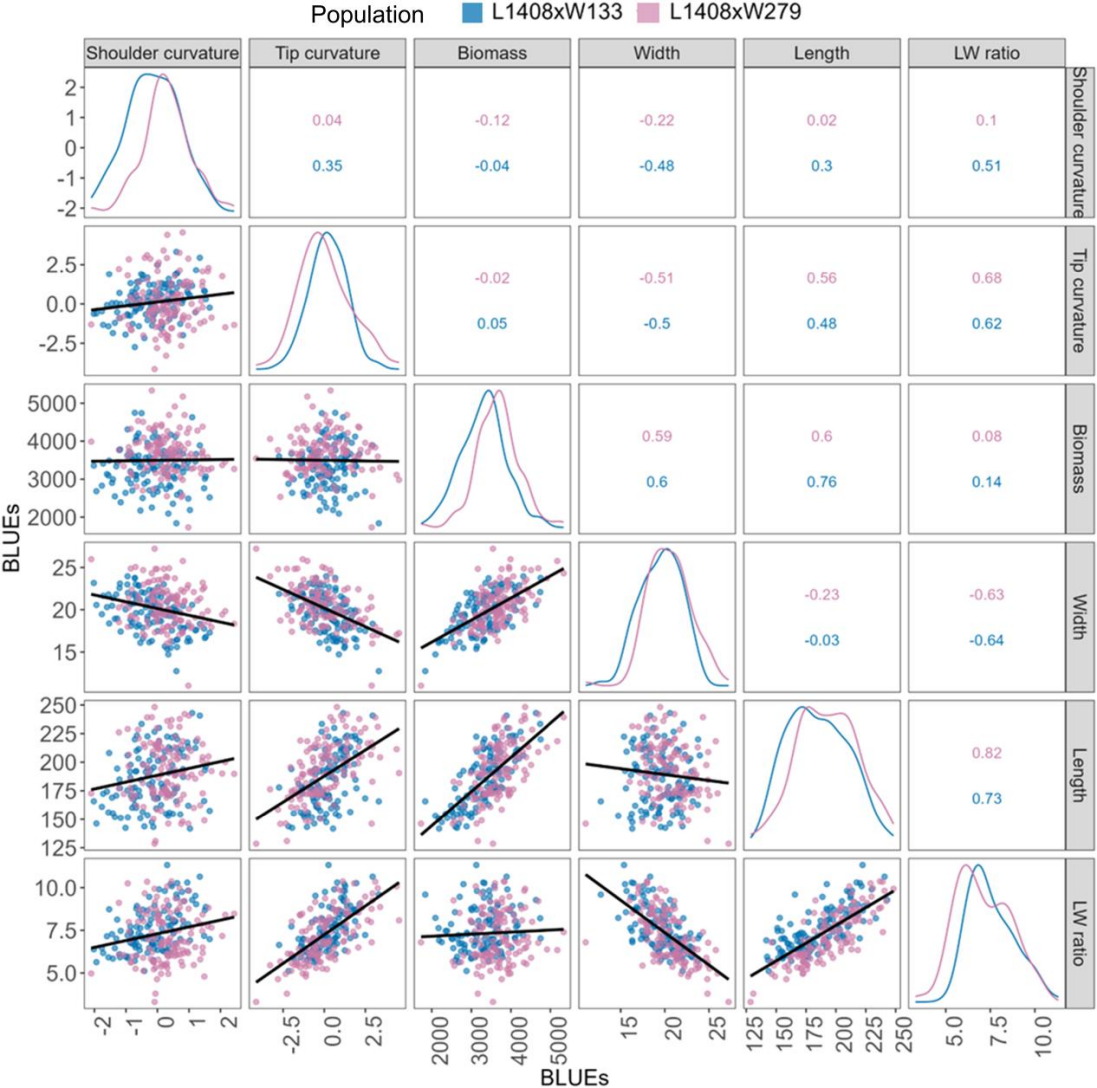
Phenotypic correlations between populations for root shape traits. Scatterplots of best linear unbiased estimates (BLUEs) below the diagonal show the relationship between traits for both populations. Pearson correlations (*r*) are shown above the diagonal for each population. The diagonal is the distribution of the trait for each population. All correlations were found to be significant at α = 0.05, except for the correlations between biomass and length-to-width ratio, biomass and tip curvature, biomass, and shoulder curvature, as well as length and width. LW, length-to-width.

Root length and length-to-width ratio showed positive correlations, ranging from 0.73 to 0.82, while width showed a negative correlation of −0.64 with length-to-width ratio across populations (Fig. 2). Consistent with previous studies, positive correlations were found between length and biomass (Vega and Goldman 2023; Fig. 2). No significant correlations were observed between length and width, as well as between biomass and length-to-width ratio. Biomass has been identified as a trait related to root size, while length-to-width ratio has been associated with root shape. Moreover, no significant correlations were detected between biomass and either shoulder curvature or tip curvature (Fig. 2).

#### Variance partitioning and heritability

The broad-sense heritability on an entry-mean basis was 0.70 for width and 0.80 for length-to-width ratio, indicating high precision in the measurement of these traits for both mapping populations (Table 1). For shoulder curvature, tip curvature, biomass, and length the broad-sense heritability ranged from 0.46 to 0.75 across years and populations (Table 1). These heritability estimates align with previous studies that have reported similar estimates for length-to-width ratio and length, which are traits related to root shape and comprise market class in carrots (Turner *et al*. 2018; Brainard *et al*. 2021).

**Table 1.** Variance partitioning and broad-sense heritability (H^2^) on an entry-mean basis for root shape traits in the carrot mapping populations L1408 × W133 and L1408 × W279 in a 2-year field trial.

	Variance components					
Source	Shoulder curvature (PC-score)	Tip curvature (PC-score)	Biomass (m^2^)	Width (mm)	Length (mm)	Length-to-width ratio
	**Population L1408×W133**					
Year (Y)	0.18	1.82	0.003	5.66	281	1.58
Genotype (G)	0.51	0.56	0.225	3.75	498	1.43
G × Y	0.08	0.36	0.059	1.10	0	0.28
Block/Y	0	0	0.011	0.07	15	0.03
Residual	0.59	1.84	0.401	4.41	639	0.97
	**Broad-sense heritability**					
	0.73	0.46	0.63	0.69	0.75	0.79
	**Population L1408×W279**					
Year	0.87	1.87	0.199	11.36	78	1.67
Genotype	0.31	1.25	0.183	5.20	516	2.21
G × Y	0.02	0.13	0.032	1.55	8	0.61
Block/Y	0.01	0.01	0.000	0.08	0	0.01
Residual	0.94	4.07	0.531	5.65	781	0.96
	**Broad-sense heritability**					
	0.56	0.54	0.55	0.70	0.72	0.80

Phenotypic data across years were combined for QTL mapping as the genotype variance exceeded the genotype × year interaction by a factor of three for all traits, except for tip curvature where the ratio was approximately 1.5 (Table 1). Tip curvature is a trait influenced by environmental factors (Vega and Goldman 2023) and with moderate to low estimates of heritability (Table 1; Brainard *et al*. 2021).

### Linkage map quality

Hierarchical clustering confirmed that each mapping population behaved as a single F_2:3_ population (Supplementary Fig. 6), despite both mapping populations were derived from multiple founders and F_1_ plants, and unexpected genetic heterogeneity was observed in all founders (Supplementary Table 1).

A separate linkage map was constructed for each mapping population, using 2,150 GBS-derived SNP markers for L1408 × W133 and 341 markers for L1408 × W279 (Table 2). The length of the linkage map was 690 cM for L1408 × W133 and 406 cM for L1408 × W279. Both map lengths fell within the range of carrot mapping populations (Ellison *et al*. 2017; Turner *et al*. 2018; Bannoud *et al*. 2019, Coe *et al*. 2023). Markers were ordered according to the physical position of version 3 of the carrot genome (Supplementary Figs. 7 and 8). Linkage map resolution ranged from 0.3 to 1.2 markers/cM across populations (Table 2), consistent with the linkage maps reported by Parsons *et al*. (2015) and Bannoud *et al*. (2019).

**Table 2.** Summary statistics of the linkage maps for carrot F_2:3_ mapping populations L1408 × W133 (*n* = 119) and L1408 × W279 (*n* = 128).

Chromosome	Number of markers	Map statistics (cM)		
		Length	Average spacing	Maximum spacing
Population L1408 × W133				
1	548	135.9	0.2	6.5
2	267	97.5	0.4	7.3
3	275	96.4	0.4	8.2
4	140	62.4	0.4	6.0
5	219	89.6	0.4	10.1
6	296	52.4	0.2	5.7
7	121	72.7	0.6	8.3
8	186	37.5	0.2	5.0
9	98	46.0	0.5	5.5
Overall	2150	690.4	0.3	10.1
Population L1408 × W279				
1	31	53.0	1.8	11.7
2	30	42.0	1.4	7.4
3	62	74.5	1.2	13.5
4	70	31.7	0.5	6.2
5	37	32.3	0.9	4.7
6	26	45.1	1.8	11.9
7	45	20.5	0.5	5.8
8	24	69.6	3.0	22.5
9	16	37.5	2.5	7.8
Overall	341	406.2	1.2	22.5

The maximum marker spacing was 22.5 cM on chromosome 8 for the L1408 × W279 population. Genetic heterogeneity in the founders (Supplementary Table 1) resulted in reduced marker coverage on the proximal arms of chromosomes 2, 4, and 6 in both populations, as well as on chromosomes 7 and 8 in populations L1408 × W133 and L1408 × W279, respectively (Supplementary Figs. 7 and 8). This reduced coverage was attributed to a higher proportion of heterozygous to homozygous markers in the centromeres and at the proximal and distal ends of the chromosomes in both populations (Supplementary Figs. 9 and 10). Heterozygosity hotspots in our populations closely coincided with the reported positions of chromosomal centromeres and repetitive regions according to the telomere-to-telomere carrot genome assembly (Supplementary Figs. 9 and 10; Wang *et al*. 2023). An explanation for the observed high heterozygosity is that instead of these regions being heterozygous variations in the DNA sequence (e.g. each founder contributing a different allele at this locus), these heterozygous SNPs could be artifacts that resulted from the mapping of reads of a given repetitive DNA sequence. In this scenario, the two copies of repetitive DNA may only differ by a single SNP and during read alignment, the software might interpret them as heterozygous SNP calls across the entire mapping population when they are not truly heterozygotes. True heterozygous calls are not expected at high frequency on an F_2:3_ mapping population, thus we filtered for heterozygote marker frequencies outside the range of 0.1< or >0.90 to avoid this issue. In addition, heterozygous markers in the founders were excluded from the initial map construction, but 18 heterozygous markers were added to the linkage map of the L1408 × W279 population to improve coverage in chromosomes 3, 8, and 9 (Supplementary Table 2). The higher proportion of heterozygous to homozygous markers in the centromeres and chromosome ends of both populations may be characteristic of the outcrossing mating system of carrots which maintain high levels of heterozygosity due to severe inbreeding depression (Glémin et al. 2006; Rong *et al*. 2010, Iorizzo *et al*. 2013; Iorizzo *et al*. 2016).

As expected, genome-wide LD was slow decaying for both populations. In population L1408 × W133, a value of *r*^2^ = 0.15 intersected physical distance at 28 Mb and genetic distance at 58 cM. In population L1408 × W279, a value of *r*^2^ = 0.15 intersected physical distance at 26 Mb and genetic distance at 29 cM (Supplementary Fig. 11).

### QTL analysis of root shape traits in two carrot mapping populations

**Fig. 3.**
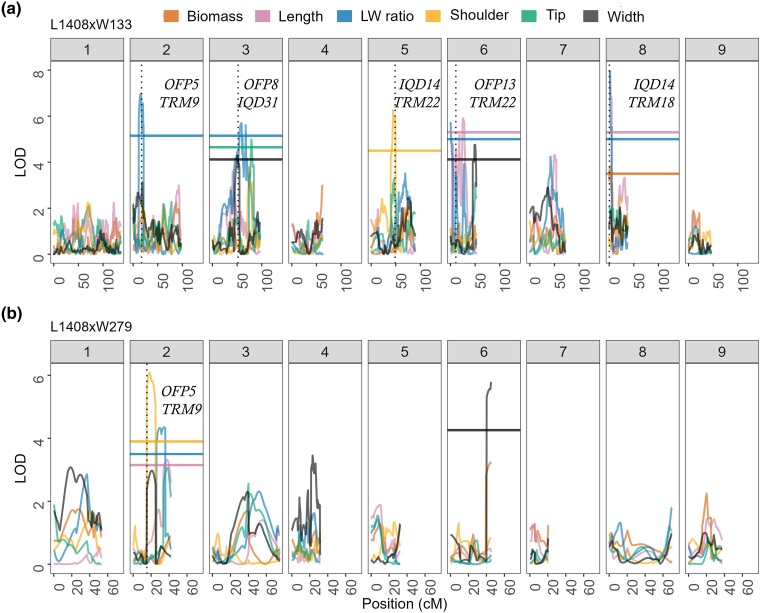
Composite interval mapping (CIM) of root shape traits in the F_2:3_ L1408 × W133 (a) and L1408 × W279 mapping populations (b). The y-axis represents the logarithm of odds (LOD). The LOD significance threshold (α = 0.05, 1,000 permutations) for each variable is indicated by the horizontal lines of the same color. The annotated known position of predicted carrot proteins associated with biological shape within the 1.5-LOD support interval, and less than 2 Mb of significant QTLs are represented by black vertical dotted lines in each chromosome and annotated. *OFPs* are *OVATE Family Proteins, TRMs* are *TONNEAU1 Recruiting Motif*, and *IQDs* are IQ67 domain. The x-axis represents the genetic position (cM) across nine chromosomes.

Significant QTL regions associated with root shape traits were identified on chromosomes 2, 3, 5, 6, and 8 in the L1408 × W133 population (Fig. 3a, Supplementary Table 3). Chromosomes 2 and 5 contained single QTLs for length-to-width ratio, and shoulder curvature, respectively (Fig. 2a). Chromosomes 3, 6, and 8 harbored QTLs for width, length-to-width ratio, and tip curvature (Fig. 3, Supplementary Table 3). These QTLs on chromosomes 3, 6, and 8 colocalized because of strong phenotypic correlations (Fig. 2).

The 1.5 LOD support interval for QTLs on chromosomes 2, 5, 6, and 8 encompassed four previously uncharacterized carrot genes: DCAR_027681, DCAR_017186, DCAR_21448, and DCAR_008585 (Supplementary Table 4). These uncharacterized genes were identified as *TRM* homologs through BLAST searches and motif alignment (Supplementary File 1). Further, three of these four predicted carrot genes exhibited the M8 motif, while the fourth gene had the M2 motif (Supplementary File 1). Both M8 and M2 motifs have been described by Wu *et al*. (2018) as conserved *TRM* motifs. Multiple sequence alignment revealed relationships between *TRM* homologs in carrot and tomato (Supplementary Fig. 12). To simplify nomenclature, the uncharacterized carrot genes were renamed after their related tomato homologs (Supplementary Fig. 12) and annotated as such in Fig. 3.

### Reproducible QTLs

**Fig. 4.**
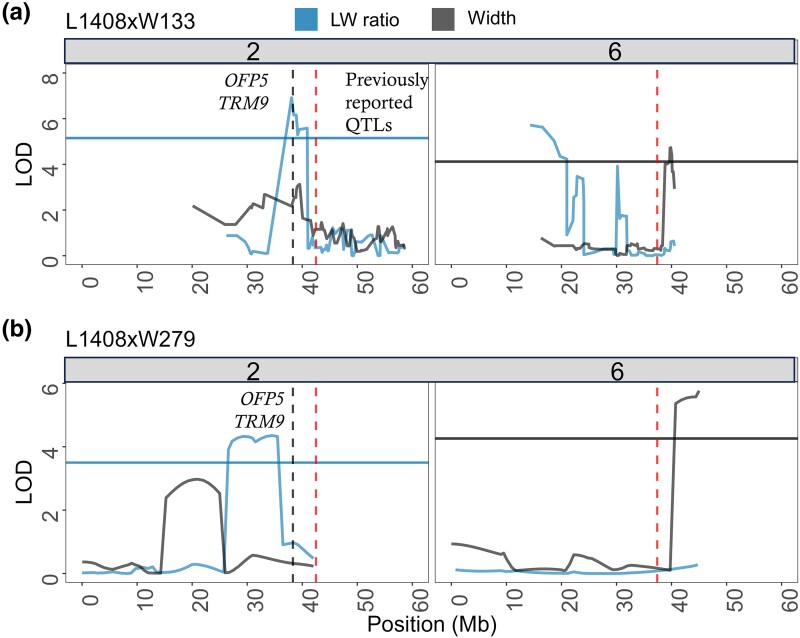
Reproducible QTL peaks identified for length-to-width ratio on chromosome 2 and root width on chromosome 6 in F_2:3_ mapping populations L1408 × W133 (a) and L1408 × W279 (b). Dashed black vertical lines indicate annotated positions of predicted regulators of shape in carrots, namely OVATE Family Proteins 5 (*OFP5*) and TONNEAU1 Recruiting Motif 9 (*TRM9*). Additionally, dashed red vertical lines mark the approximate locations of previously reported chromosomal regions (QTLs) associated with carrot root shape traits (Macko-Podgórni *et al*. 2017; Turner *et al*. 2018; Brainard *et al*. 2022). The x-axis represents the physical position in mega bases (Mb) across chromosomes 2 and 6.

Reproducible QTLs for width on chromosome 6 and for length-to-width ratio on chromosome 2 were identified in both populations (Fig. 4). The 1.5-LOD support intervals overlapped across the two mapping populations, confirming the reproducibility of QTLs for length-to-width ratio on chromosome 2 and width on chromosome 6 (Fig. 4 and Supplementary Table 3). These two QTLs explained 16–17% of width and 14–20% of the length-to-width ratio phenotypic variation across both mapping populations (Supplementary Table 3).

**Fig. 5.**
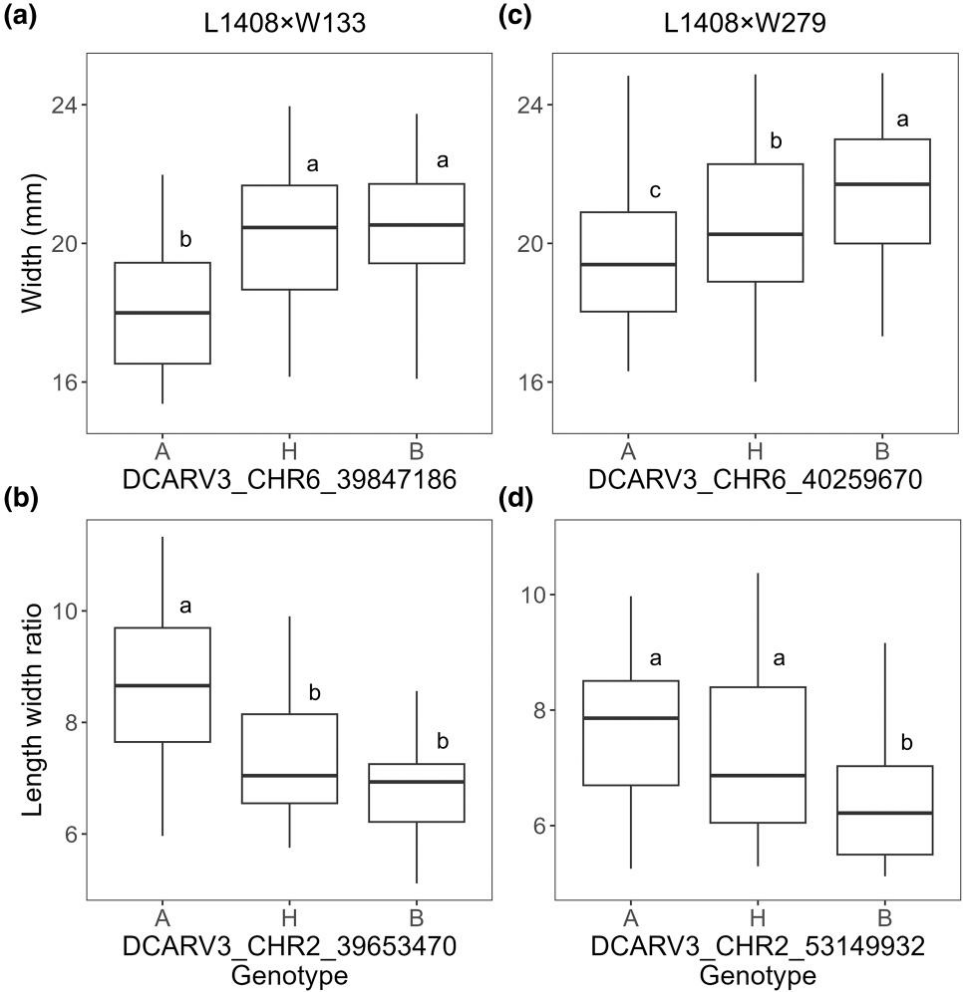
Relationship between genotype at SNP loci of reproducible QTLs for width (mm) and length-to-width ratio in carrot F_2:3_ populations L1408 × W133 (*n* = 119) and L1408 × W279 (*n* = 128). Effect plots for width (a) and length-to-width ratio (b) in population L1408 × W133. Effect plots for width (c) and length-to-width ratio (d) in validation population L1408 × W279. Genotypes “A” (L1408) and “B” (W133 or W279) represent the two homozygous states, while “H” denotes the heterozygote. Boxplots with different uppercase letters indicate significant differences at α = 0.05. Multiple comparisons were corrected using the Šidák correction.

In the L1408 × W133 population, individuals that inherited either one or two copies of the “W133” (B) allele at the QTL for width on chromosome 6 resulted in a width increase of 3.0 mm or 1.0 standard deviation units (Fig. 5a). No significant difference in width was detected between individuals that inherited either one or two copies of the “W133” allele (Fig. 5a), and the dominance degree was 0.7 (Supplementary Table 3). These two findings suggest partial dominance to the “W133” phenotype in width (Supplementary Table 3, Fig. 5a).

In the L1408 × W279 population, individuals carrying two copies of the “W279” (B) allele at the root with QTL on chromosome 6 showed an increase of about 3.2 mm or 1.2 standard deviation units in root width while individuals inheriting a single copy of the “W279” allele showed an increase of 1.8 mm or 0.73 standard deviation units (Fig. 5c). Significant differences in width were detected between individuals that inherited 0, 1, or 2 copies of the “W279” allele, illustrating the additive relation between the trait and the underlying QTL.

Taken together, the data suggest that inheritance of either “W133” or “W279” alleles at this root width QTL on chromosome 6 resulted in increased root width. In population L1408 × W133, the relation was partially dominant to the W133 phenotype while in population L1408 × W279 the relationship was additive (Fig. 5a and c).

For the length-to-width ratio QTL on chromosome 2, two copies of the “W133” allele at the QTL reduced the score by 2.0 units or approximately 1.2 standard deviation units in the L1408 × W133 population (Fig. 5b). No significant difference was detected between individuals that inherited either one or two copies of the “W133” allele (Fig. 5b). However, individuals that inherited 1 or 2 copies of the “W133” allele were significantly different from those that inherited 0 copies. Similar trends were observed in the L1408 × W279 population. Two copies of the “W279” allele reduced the length-to-width ratio by 2.0 units or approximately 1.3 standard deviation units (Fig. 5d). However, no statistical difference was found in length to width ratio in individuals that inherited either 0 or 1 copies of the alternative “W279” allele.

In sum, individuals that inherited two copies of the “W133” allele showed no statistical difference from heterozygote individuals in length-to-width ratio, indicating partial dominance to the “W133” parent phenotype (Fig. 5b). In contrast, in population L1408 × W279 only individuals that inherited two copies of “W279” showed significant differences in length-to-width ratio compared with individuals that inherited one or zero copies (Fig. 5d), suggesting partial dominance to the “L1408” parent phenotype. Across both populations the effects for width and length-to-width ratio were maintained, but the gene action was population specific. Dominance degree values ranged between 0.3 and 0.7 for both traits which suggest partial dominance, and intermediate phenotypes for the heterozygote individuals (Supplementary Table 3, Fig. 5d).

### Population-specific QTLs

In the L1408 × W133 population, QTLs for length-to-width ratio were found on chromosomes 3, 6, and 8, along with an additional QTL for root width on chromosome 3. These population-specific QTLs collectively explained an additional 53% of the phenotypic variation in length-to-width ratio and an additional 16% in root width, resulting in a total of 73 and 33% variance explained for length-to-width ratio and root width, respectively (Supplementary Table 3).

QTLs for tip curvature on chromosome 3 (16% variance explained), shoulder curvature on chromosome 5 (22% variance explained), and root length on chromosomes 6 and 8 (40% variance explained) were also identified (Supplementary Table 3). A QTL peak for biomass explained 14% of variance and mapped to the same region of chromosome 8 (Fig. 3, Supplementary Table 3).

In the L1408 × W279 population, one additional QTL for shoulder curvature and one QTL for length were identified in the reproducible region on chromosome 2, explaining 20 and 11% of the phenotypic variation, respectively (Fig. 3, Supplementary Table 3). Predicted gene members of the *TRM*-*OFP* and *IQD* plant organ shape regulon fell within the 1.5 LOD QTL support interval of population-specific root shape QTL peaks in chromosomes 3, 5, and 8 (Fig. 3, Supplementary Table 4). In chromosomes 8 and 3, QTL peaks for length and length to width ratio were also colocalized suggesting tight genetic linkage and correlations among traits. In chromosome 5, however, a single QTL for shoulder curvature was identified and was in very close proximity to predicted gene members of the *TRM-OFP* and *IQD* regulon (Fig. 3). All chromosomal regions harboring significant QTLs identified in the L1408 × W279 population were also identified in the L1408 × W133 population.

## Discussion

### Linkage maps

Our linkage mapping approach identified reproducible QTLs on chromosomes 2 and 6 using two independent populations, despite coverage gaps in proximal ends of several chromosomes (Supplementary Figs. 7 and 8). Because centromeres are composed of highly repetitive and methylated sequences, the GBS *ApeKI* enzyme is less efficient and therefore large DNA fragments are produced during the reduced representation step of GBS, which are later discarded at the read size selection step, resulting in reduced marker coverage in centromeres and telomeres (Aballay *et al*. 2021). In addition, unexpected genetic heterogeneity of the W279 founders contributed to a reduced linkage map in the L1408 × W279 map.

A 22.5 cM gap was found in chromosome 8 of population L1408 × W279. However, Turner *et al*. (2018) reported a linkage map with an 18 cM gap in chromosome 6, which is only a ∼5 cM difference from largest gap reported here, despite Turner *et al*. (2018) used a larger population size (*n* = 461) and a comparable number of markers (640). These limitations likely arise from phenomena like segregation distortion, reference genome bias, inbreeding depression, residual heterozygosity, and genetic heterogeneity in inbred lines, rather than being population specific.

### QTLs associated with root shape traits composing market class

Four QTLs controlling length-to-width ratio were identified, collectively explaining 73% of the phenotypic variation (Supplementary Table 3). The QTL on chromosome 2 for length-to-width ratio was reproducible (Fig. 4), and QTL intervals for the same trait on chromosomes 2, 3, and 8 coincided with chromosomal regions containing predicted gene members of the *OFP-TRM* and *IQD* regulon (Fig. 2, Supplementary Table 3). A second reproducible region on chromosome 6 harbored a QTL for root width in both populations (Fig. 4).

In the L1408 × W133 population, genomic regions on chromosomes 3, 5, and 8 showed significant QTLs with 1.5 LOD intervals that encompassed carrot *OFP-TRM* and *IQD* predicted genes. However, these same regions did not exhibit such QTLs in the L1408 × W279 population (Fig. 3). This observation suggests variations in QTLs across different mapping populations, a concept discussed in linkage mapping literature (Holland 2007; Myles *et al*. 2009). Biparental populations represent the genetic diversity existing in only two parents, which could limit the scope of identified QTLs to the studied genetic backgrounds (Michel *et al*. 2022).

Gene action may also be subject to population-dependent variation. For example, the length-to-width ratio QTL on chromosome 2 showed partial dominance to the “W133” phenotype in the L1408 × W133 population (Fig. 5b) but switched to the “L1408” parent allele in the L1408 × W279 population (Fig. 5d).

### Colocalization of QTLs

Consistent with our results, previous studies evidenced colocalization of QTLs in chromosome 2 for correlated carrot root traits, including length, digital biomass, and tip fill (Turner *et al*. 2018). Tip fill is a related measure of the tip curvature phenotype presented here. The colocalization of QTLs may suggest shared genetic mechanisms that impact root morphology. The presence of colocalized QTLs in independent populations has been documented in maize multiparental MAGIC populations, where specific chromosomal regions were associated with both plant height and flowering time (Michel *et al*. 2022).

The proximity of QTLs reported in this study, ranging from 1.2 to 3.4 Mb to those previously identified through linkage mapping and association studies on chromosome 2 (Fig. 4) supports the involvement of this genomic region of chromosome 2 in shaping carrot root traits (Macko-Podgórni *et al*. 2017; Turner *et al*. 2018; Brainard *et al*. 2022). The presence of a shoulder curvature QTL in the L1408 × W279 population, 1.7 Mb away to the estimated location of a similar shoulder trait QTL reported by Macko-Podgórni *et al*. (2017), adds evidence to the genetic significance of loci in chromosome 2 in root shape control (Supplementary Table 4).

Brainard *et al*. (2022) results indicate the presence of an *OFP8-like* transcription factor less than 40 kb of a QTL identified for carrot maximum root width on chromosome 3. The QTL identified by these authors is within the 1.5 LOD interval of colocalized QTLs for tip curvature, width, and length-to-width ratio identified in chromosome 3 in this study. Independent identification of genetic regions controlling root shape highlights the importance of these genomic regions in carrot root shape control.

### Genetic linkage and candidate genes

Genome-wide LD in carrot diversity panels is fast decaying (Ellison *et al*. 2018; Brainard *et al*. 2022) and exhibits a nonmonotonic nature (Schaid *et al*. 2018), but as expected for biparental populations, our findings suggest very slow monotonic genome-wide LD decay (Supplementary Fig. 11). In response to large blocks of LD, affecting QTL size precision, the candidate gene search was focused on predicted carrot genes in the *OFP-TRM* and *IQD* regulon. BLAST searches identified 41 *TRMs*, 22 *OFPs*, and 45 *IQDs* predicted genes in carrot.

Members of this plant regulon including *OFP5* and *TRM9* fell within the 1.5 LOD support interval for the length-to-width ratio QTL on chromosome 2 (Figs. 2 and 3, Supplementary Tables 3 and 4). The locus DCAR_007928 is a predicted repressor of elongation OFP5 and was proposed as one candidate gene for the length-to-width ratio QTL on chromosome 2 for population L1408 × W133. We excluded the candidate gene DCAR_007928 from consideration in L1408 × W279 as it lies outside the QTL support interval for this population. However, it is just 1.4 Mb away from the QTL region. Despite differing candidate genes, reproducibility of QTLs for both populations on chromosome 2 is supported by an overlap in the 1.5 LOD interval (15 Mb). Variation is likely due to fewer SNP markers in Population L1408 × W279, precluding the identification of an SNP marker in LD at the exact physical position as in Population L1408 × W133. Evidence for reproducibility of the QTL stems from overlap in physical position and identical gene action in two populations.

Predicted *TRM22*, *TRM18*, and *IQD14* were also found in the support interval of significant QTLs in chromosomes 5 and 8 (Fig. 3). Genes in the *TRM* and *OFP* families interact and function as transcription factors, influencing gene expression and plant organ shape in tomato and Arabidopsis (Snouffer *et al*. 2020; Li *et al*. 2023). The *IQD* pathway also encodes proteins that regulate cell proliferation and expansion, contributing to fruit shape determination (Wendrich *et al*. 2018; Yang *et al*. 2020).

*OFP5* and *OFP8* homologs found in carrots are predicted repressors of elongation (Supplementary Table 4). In addition, Arabidopsis orthologs *AtOFP5*, *AtOFP8*, and *AtOFP13* have been confirmed as repressors of organ elongation (Wang *et al*. 2007, Wang *et al*. 2011; Zhang *et al*. 2020). In tomatoes, *TRM9*, *TRM18*, and *TRM22* have been associated with cellular organization and shoot outgrowth which may suggest their role in modulating phytohormones (Namphengsone 2019).

Although the *OFP-TRM* and *IQD* regulon has been recognized as a master regulator of shape in fruit, grains, and potato tubers (Wu *et al*. 2018; Guo *et al*. 2021; Li *et al*. 2023), its involvement in roots has been limited to one study in radish (*Raphanus sativus*, Wang *et al*. 2020). The present study is the first linkage mapping report connecting the *OFP-TRM* and *IQD* regulon to carrot root traits that constitute market class.

The influence of the established mechanisms of the *OFP-TRM* and *IQD* plant regulon on carrot root shape remains uncertain. This ambiguity arises from the composition of carrot roots, comprising both root and hypocotyl tissue originating from secondary growth of cambium with parenchyma tissues. Carrot roots have swollen and expanded xylem and phloem tissues beyond the primary vascular tissues (Goldman 2020) which contrasts with the division patterns and cell arrangement of fruit tissue. Nevertheless, the extension of the *OFP-TRM* and *IQD* regulon's influence beyond fruit shape has been well demonstrated in potato (*Solanum tuberosum* L.) tubers, which are a modified stem. Back in 1994, van Eck *et al*. identified the *Ro* locus, responsible for round tuber shape, on chromosome 10. Studies subsequently found QTLs for tuber shape mapping to the same locus in diploid potato F_2_ populations (Endelman and Jansky 2016) and molecular markers were developed for the locus (Chen *et al*. 2019). In 2018, Wu et al. conducted fine mapping of the *Ro* locus and confirmed that the potato *Ro* locus is controlled by *StOFP20,* an ortholog of tomato *SlOFP20*. The function of *StOFP20* and its interaction with *TRM* members was later experimentally confirmed by Ju *et al*. (2023).

Although we limited our search for candidate genes to the *OFP-TRM* and *IQD* plant shape regulon, other families of genes may also contribute to root shape control. Our large linkage blocks include the possibility of harboring genes in other pathways previously reported to be involved in carrot root formation such as the *AT-hook containing nuclear localized* (*AHL*) gene family or other undiscovered gene families. *DcAHLc1*, for example is a member of the *AHL* gene family and was proposed as a candidate for carrot root formation (Macko-Podgórni *et al*. 2017). Members of the *AHL* gene family also fell within QTL confidence intervals controlling root shape in mapping studies (Turner *et al*. 2018). Further characterization of the *AHL* family in carrots has demonstrated that their role is mainly in plant growth and storage root development (Machaj and Grzebelus 2020), which opens up the possibility that multiple mechanisms may be responsible for root development and shape patterning of carrots.

### Genetic mapping in carrots

Although inbred lines with 99.6% homozygosity have been reported (Wang *et al*. 2023), the outcrossing mating system of carrots presents challenges in obtaining homozygous inbred lines. Inbreeding depression also tends to result in lines with genetic heterogeneity that show uniform and stable phenotypes in self- or sib-mated lines (Rubatzky *et al*. 1999; Simon 2021). This is compounded by patterns of segregation distortion observed in chromosomes 1, 4, 8, and 9 (Grzebelus *et al*. 2014; Bannoud *et al*. 2019; Iorizzo *et al*. 2019). Limited availability of homozygous inbred lines complicates the linkage mapping problem. To overcome these limitations, doubled haploid research has gained attention (Andersen *et al*. 1990; Meyer *et al*. 2022), offering potential avenues for carrot mapping and breeding. Searching alleles that provide inbreeding resistance in carrots, along with reproducible genotyping technologies and high-quality telomere-to-telomere genome assemblies, has the potential to advance breeding and mapping efforts for of agronomically and horticulturally important traits in carrot.

This mapping study provides insights into the genetic basis of root shape traits associated with carrot market class, indicating a potential link with the *OPF-TRM* and *IQD* regulon, which has been well established in tuber and aerial plant organ shape (Wu *et al*. 2018) but with very limited focus on root traits. A better understanding of genetic shape control in carrot roots may enhance the development of improved varieties, expanding current carrot market classes.

## Supplementary Material

jkae041_Supplementary_Data

## Data Availability

SNP markers filtered for MAF ≥ 0.05, depth of 20 for 95% of the population and only biallelic sites are provided as VCF files along with linkage maps that include phenotypes. Excel files with phenotypes computed from digital pipelines and annotated R scripts used to create linkage maps and to summarize phenotypic datasets are available in https://zenodo.org/records/10023296 (DOI: 10.5281/zenodo.10023295). Supplementary material is available locally at https://zenodo.org/records/10626038 (DOI: 10.5281/zenodo.10257998). Supplementary figures includes Supplementary Figs. 1–12. Supplementary tables includes Supplementary Tables 1–4. Supplementary File 1 includes multiple sequence alignment to conserved Tonneu Recruiting Motifs (TRM) for four previously uncharacterized predicted carrot genes DCAR_008585 (LOC108208046), DCAR_017186 (LOC108220104), DCAR_021448 (LOC108228003), and DCAR_027681 (LOC108200088). Supplementary File 2 includes amino acid sequences of know regulators of shape in tomato (*Solanum lycopersicum*) and predicted genes in carrot *(D. carota* var. *sativus*) with sequence homology to the shape regulon *OFP*-*TRM* and IQD for assembly, GCA_001625215.1, bioproject PRJNA268187. Visual representation of the relationships among gene sequences of carrot and tomato *TRMs* homologs are presented in Supplementary Fig. 12. Supplemental material available at G3 online.
