# Correction to: Linkage mapping of root shape traits in two carrot populations

**DOI:** 10.1093/g3journal/jkae227

**Published:** 2024-09-27

This is a correction to: Andrey Vega, Scott H Brainard, Irwin L Goldman, Linkage mapping of root shape traits in two carrot populations, *G3 Genes|Genomes|Genetics*, Volume 14, Issue 4, April 2024, https://doi.org/10.1093/g3journal/jkae041

In the originally published version of the manuscript, the email contacts of the corresponding authors were transposed. These have now been emended in the article.

