# Supplementary material for: Linkage mapping of root shape traits in two carrot populations
Source: G3 (Bethesda). 2024 Feb 27;14(4):jkae041. doi: 10.1093/g3journal/jkae041 (PMC10989876; doi:10.1093/g3journal/jkae041)
Supplement: jkae041_Supplementary_Data [file jkae041_supplementary_data.zip › File_S1_G3-2023-404760.pdf]

File S1

Conserved Tonneu Recruiting Motifs (TRM) motif alignment to four carrot gene amino acid sequences

Andrey Vega, Scott H. Brainard, Irwin L. Goldman

Department of Plant and Agroecosystem Sciences. University of Wisconsin-Madison, Madison, Wisconsin, 53706, United States of America.

Two TRM conserved motifs were aligned to four previously uncharacterized predicted carrot genes DCAR_008585 (LOC108208046), DCAR_017186 (LOC108220104), DCAR_021448 (LOC108228003) and DCAR_027681 (LOC108200088). All genes are within the 1.5 LOD support interval of QTL peaks controlling length, width and length-to-width ratio in chromosomes 2,5,6 and 8.

We identified the M2 and M8 conserved TRM motifs of tomato (*Solanum lycopersicum*) reported in Wu et al. (2018) in the previously uncharacterized carrot gene amino acid sequences.

Alignment of tomato TRMs conserved motifs to the carrot amino acid sequences was performed using MAST (Timothy et al. 1998) (https://meme-suite.org/meme/doc/mast.html).

```
MOTIF ID   Size Sequence
----- -- ------ ----- -------------------
   1 M8 -      18 GQKPPSVVARLMGLDELP
   2 M2 -      20 LEVEEVGLEIEKELNDLIDE
```

PAIRWISE MOTIF CORRELATIONS:

```
MOTIF     1
----- -----
  2   0.13
No overly similar pairs (correlation > 0.60) found.
```

Columns whose match to the motif have a positive score are indicated by a plus sign. Loci where the sequence matches a motif are labeled in red.

DCAR_021448 (DCARv2_Chr6:22864332..22868406)
LENGTH = 940 COMBINED P-VALUE = 3.48e-09 E-VALUE = 1.4e-08

```
                 [M8]
                 GQKPPSVVARLMGLDELP
                 + + +  +++++++++ ++
76
FSNGKKNGARNVDSGQKQGMKTPTLVARLMGLDSLPAVQRNKSKKGYELGVDRGEEIATDSCALARQQ
IEVEKAG
```

[M8]
GQKPPSVVARLMGLDELP
++ ++   + + +

526
NGDVSFTFSSSKKNKERILAKPDDREYQSECTCTHSSQRSSAFDTINRKRQTCFQKLPSPGDTLSVLLEQ
KLKEL

DCAR_017186 (DCARv2_Chr5:12549833..12553892)
LENGTH = 942 COMBINED P-VALUE = 2.36e-06 E-VALUE = 9.4e-06

[M8]
9.6e-10
GQKPPSVVARLMGLDELP
+ +++++++++ ++

76
GTRNVDTGQKYAMRTPTLVARLMGLDSLPAVQRGKIKKISSDRIEVDTGGKFASDCCQFGGQHKKFKPE
GSKHEL

DCAR_027681(DACRv2_Chr8:20635645..20639639)
LENGTH = 780 COMBINED P-VALUE = 2.57e-03 E-VALUE =   0.01

[M8]
3.7e-07
GQKPPSVVARLMGLDELP
+++ +++++ +

1
METKEQTPSVIARLMGFYETRHQRPIHKKYRVLSEDYLRKSASIDLLLKKSSCNGRSFRMSRVKMPEFKD
SFEGQ

DCAR_008585 (DCARv2_Chr2:43316019..43317136)
LENGTH = 340 COMBINED P-VALUE = 3.05e-02 E-VALUE =   0.12

[M2]
2.1e-05
LEVEEVGLEIEKELNDLIDE
+ +++++++ +

301 MERGVKWNKFDEDEQELGLEIEKQLLNCLVDEVLFDFLDL

References

Wu S, Zhang B, Keyhaninejad N, Rodríguez GR, Kim HJ, Chakrabarti M, Illa-Berenguer E, Taitano NK, Gonzalo MJ, Díaz A, Pan Y, Leisner CP, Halterman D, Buell CR, Weng Y, Jansky SH, van Eck H, Willemsen J, Monforte AJ, Meulia T, van der Knaap E (2018) A common genetic mechanism underlies morphological diversity in fruits and other plant organs. Nature Communications. doi: 10.1038/s41467-018-07216-8

Timothy L. Bailey and Michael Gribskov (1998). "Combining evidence using p-values: application to sequence homology searches", Bioinformatics, 14(1):48-54.
