# Supplementary material for: Linkage mapping of root shape traits in two carrot populations
Source: G3 (Bethesda). 2024 Feb 27;14(4):jkae041. doi: 10.1093/g3journal/jkae041 (PMC10989876; doi:10.1093/g3journal/jkae041)
Supplement: jkae041_Supplementary_Data [file jkae041_supplementary_data.zip › File_S2_G3-2023-404760.pdf]

File S2

Amino acid sequences of know regulators of shape in tomato (*Solanum lycopersicum*), and predicted genes in carrot *(Daucus carota* var. *sativus*) with sequence homology to the shape regulon *OFP-TRM* and IQD. Assenbly, GCA_001625215.1, bioproject PRJNA268187

Andrey Vega, Scott H. Brainard, Irwin L. Goldman

Department of Plant and Agroecosystem Sciences, University of Wisconsin-Madison, Madison, Wisconsin, 53706, United States of America.

Carrot Tonneu Recruiting motif (TRM) amino acid sequences with homology to tomato TRMs.

>DCAR_002502 (DCARv2_Chr1:29924732..29928697)
MGVERERSKSGGGYVGGFLHLFDWNTKSRKNLFSNKSNAQDILFISVISVEQLKQKTINDHNHP
TTQLYLMDQDDSLARSSFKGSSEYS
CASSVTDDDVGGSKAPSVVAKLMGLESLPTSNFSEAYSTPFLDSRSLKDAYYYRKSEEFHEDQ
QIMRSGNVFDRSQNTLRNNLDSKIHKSISKPFEKFQS
EVLPPRSAKSIPLTHHKLLSPIKSGGFLPSKNAVHIMEAAARIFDSGSQVTTKAKLPSVGSSVPLK
VRDLKERVEAAKKPSNLVESSQKPLESIAAKNIK
GQSMTKSWNGSLDSKTFRASSNSEEGSVGSKNKGKSVSLAVQAKANVQKRGLSPNHSRTSA
GQKEQGEMTTNQIFRSQPSLQRSSHKKSSTNNSSSVLRQ
NNQKQNCIAEREKVAVKSLNSNNSQVKKVTSGDSSLGRQRNSSKNSGSNKNGSRKIEREGND
DGREVPYSSTSVTRKKRCIDGDFNFQKDQAVKSEKKGK
ADQHNGVRESKFSWAEDSRSNGMDVVSFTFTAPMGRSIPVPETSREKLEKNNAFSAEFQGKE
VFFNSSGTNRLRPSSVGHNMIEGDALSNLLEQKLREYT
LGVDSFSRKAEEVGSTTSSQDQTPLKAVVKSSKLHIEGNQMGSQTECLDGRWNPVFSSNTYT
EGHMSKHKLQEVEDMFDCGSNSSEVKNLLGCRHPSPVS
ILEHSIFAESSNSSDTGDSTSTEGLVISKQCSTSVQGQDVSNMRGSYKFRVVESDAELSDSASS
SSTRFVTTKHVTLAMTDPVGSAKWELEYVKKILCNS
EMMFKDVSTGQASQIVDPRLFDQLESCEDELKLQRKVLFDCVGECMDLRFRHYAGGGYKAW
EKGLSMARRTKWLAGEVHREISSWEAMGDCMVDELVDKD
MSSQCGSWLNFGVETFELGVEMERKILNSLLNELISDILVR

>DCAR_004631 (DCARv2_Chr1:49824171..49829769)
MASSSRAKATRKMDVRARNKEFDPNGLGKEDRFVFNLRCADSHITYNFIVFIIELRCRAAFGGS
KFTGEELDNKLCLGDEHSYEKPFMI
VTLYSDAKPQGGPDHVTFEEKPVKVDLKLNICSCIRTVLQASELNCKELSENLRSSGQLLNLFS
VHRRGLFADFLNDVLQEVSQQNAECIRWSSFIRPDT
QAFGVLGKDERSSLGQSREVFLQEKDRVFLNQGKRTTITEKTQLNCKWKLIGICNNPQALAPTH
KLLSNVKHENKQVTGSRRGSTDTPNLSSRPTVDDEL
HKSRKLLDAFDILNSNSDLFLKLLQDPNSLLMKHIESQKDMQEKTARTTTCQENNLPESGSGSA
LHKYQKPQHTVRKSLFDIMNEQECASKDDEIISTSN
SAIAPKASQKDHRRRELGKHREEGPSSAAQMATSSECATVSRSNQRDSEAKRRLSRRLKNVG
KSESVSGKETPRLTLKRILSSPKHGFVASSCPKMETED
NSLQPKNEDDSTKLAKIHETGFLTKDLSSNDCQKNLKVNDTIQSVSANLSEMPSKDSNVHSTDA
EESTSTTRQRKQYQYLKCSTQDINLENQILTSSAEV
PSSNHISKYRIELEGSFEETEEHLSPVSVLDSLFSEDVTSPSSKMNHPAKPQGEPDHATFEEKP

VKVDQVDPKLNICSCIRTVLQASELNWKELSENSRS
SGQLLNLFSVHRRDLFADFLNDVLQEVSQQNVECIRWSSFIRPDTQAFGVLGKDVVEEVMKEV
HWYLVPSILPRKLEHIVRKDMEKPQCWSGRRHDTEEI
VIQIVDDVLDESVMETISAIGEYDYFL

>DCAR_002692(DCARv2_Chr1:31899136..31902433)
MSTAVAKDRNAENQIVKQTGCMAGFFKLFDRHQFLSAKRLPAPKDVDDSDSEFVASASMKKP
EKTRTLTFPSAEVYRLIEPRSPVVVSS
RPVEIPPKSPIPVQIFEMNEGGKNSWKFSPRLSLDSRAAFDAAKGSLYRKEAERNSSDDNSRR
STSVIAKLMGIEPMPNSSSYEPITKHVELQRSASESR
ASREIFQSRLIESNSSDELKVKQRNYVSNDDNNAVNTEKFVTRSNQKRNSKSPQQRKSFYNAA
DIFPEPNQKLSIYGEIERRLKMKGIDEPMKDLETLKQ
ILEAMQLKGLLHTKPVAPVRNKDEVRGWNYSSSSEPKSPIVLMKPNRRVGQESPPRIRSKSDN
RQGVSSVSPRPQRQSIDRNATSPIRGRSNSLVKTKPL
SIETPRRVNESVNSRRVSPVSSPRRNVADQTVSNRSPRNRRPVAQSHPKDKNKNSVTVEDES
SSSISESTVSTPSHTDTERCKREYTEGRRLLNRCDKLL
NSIAEMNASESQLSPVLKSIADMNASESQPSPVSVLDSSFYKDDPCSPSPSPVMKRSISFPVGL
EEETGSPDFSSVQSKCEDEFDYSDLIYVSRILKAWT
YASEEASNHIFVHLEKQHYLKCKDSTKLSNLQRRLIFDTVTEIMKRYRDFPPWKSFSTKTWQTS
QPSLQQISLEIRKLKEHQSSDDLFEVICGVLKKDLA
GDASNGWGDCAIEMSDAVLDIERLIFKDLVGESIRDLAELSAKSTYLAPRRKLVF

>DCAR_000547 (DCARv2_Chr1:397568..400578)
MLIAQEMSKEVDSKQKPPSVVARLMGLDTLPLQQSYTASQQNHSRGSSRSQSFASFGSWKEE
QEIHQYQEQNGYKDVYEIQQSHNKFVR
HNSPHKERFDGRSVEKKMALVRQNFIELKRLSTDEKLRQSKQFRDALEVLSSNKDSFLKFLQE
PNSMFSQQLSDLHSNPLPSETKRITILKPAKMVDSYK
VTDLGKKDEEQINESSEFSQINRLSIPGFSPPPTCKLEDSRVQATRIVVLKPSPGKSHDITAVISP
LSPSTKALHVEDQYVEFEENDAKESREMAKEITR
QMCENLSHQRRDETLLPSVFCNGYTGDESSFDKSEIEFAAENLSDSEAMSPTSRHSWDYINRL
GSPYSFSTFSRASYSPESSVCREAKKRLSERWAMMAS
NRSQEQKHVKRSSSTLGEMLALSDIKRSVISEQDSIRSEQEFRGSRSCCTSDLNDEDKCDPPT
RNILRSKSVPASSTAYGGRISLEVSDPKMEKADAAKD
LTKTRSMKSLLKGRVSNLFFPRTNKSSKQKSSNSRDEIESAELPSQPSGYEVDLAIKQGELPDP
LGSWNKASPSHSFSVENEEWLLHTKAEISATKPCPS
RNPIEYQDTPSSFSVLELPFQEDDRRDSEFSDNFSLLRNGSDSPVYSSKANLIDKSPPVRSIAR
TLSQKDSCTETASLYPAKTSVVMIDAEEERQELSLL
VQTLLAAAKFGNELQFDTFLARCHSLESPLDPSLRDNYLGLIDKETLPEAKRGQRILMQKLVFD
CVNAALVELAVHELDTWKSRPSNRVHDQMLIFDSVW
TQMEWFCGEVSCVLGEIWENNSLVVERLVRKDVIEKGWNDQAMMEIDRFHKEIEVKLLEDLVQ
EAVEEFTDNLR

>DCAR_004699 (DCARv2_Chr1:50515207..50520733)
MNGFQNPQAHNIEKPFLGCLGRVVNLFDQSAGIPENRLLADKPHGAGSLLSRSRSDVSGMNP
VDSQIEDKATKSELQTTHGTPIKMLMA
QEMLKELDFKQKPPSLVAKLMGLDSLPLQKPSTASQRSHSRGSSRSKSCVSFGSWQEEQEVC
QSQKHDDYKDVYEVWQPHNKYVSDKSPKQERFDGSSIE
KRMSLVRQNFIELKRLSSDEKLRHSQKFQDALEVLSSNKDSFLQFLQEPNSMFSQQLSDMQSI

SPPSEAKRITILRPAKVVEGNKITGPVKKNGTKINEI
SRLGRVSRLDNSPGFSSPTACKLEDSQVQATRIVVLKPSPGKSHDIKAVVSPLSTSPKALHVKD
PYTGFDDDDTQESRELAEEITQPMHENMSRHRRDET
LHSSVFSNGYTGDESSFDKSEIEFAAENLSDSEAISPTSRHSWDYINRPGSSCSFSSFSRASYS
PESSVCREAKKRLSERWAMMASKGNQEQKQLRRSSS
TLGEMLALSDMNKSVICTGNLTNSEQEIRTSTSCVTGDFSNEDKCDAPARNILRSKSIPASSNAY
VGGLHSEVSDSKMERPDINKELTKTKSMTSLLKGR
VSSLFFSRNNRTGKQKASNSRDETESSELPIPSSEKDVDIRFQAAGELPDPLESSNKASPLHSLI
DEPEEDIVHTKAGVSVTEPCPLQFPVENQEQPSPI
SVLEPPFQEDDFGEPELSDNSSEVRNGLDLHVHRNSNFLDKSPPIGSVARTLSWNDSCTDDAS
PCPIKSSSVPVGPEEERQELVFLVQTLLTAAKLGNEM
QSETFFASWHSLESPLDPSLRDNYVGLIDETMHETKLRHRKSVQKLVFDCVNAALVELAVCGS
DPSKSRIHYNLQDNKSILDCLWTQMELFPDEMSFIVG
EGGDRDSLVAEELVRKQVAGKGWVDYSSLQRDDFSKEIEGKLLQELVQEAVEDFTGSL
>DCAR_002956 (DCARv2_Chr1:34333913..34337793)
MYLCFDFNLLSSLFNSLSIFCFSCQCTAGMEAPRNSLEFPVEIFQGYGQAGGDHLMYAHQEAQ
VWSEKNCFPSEAPMKKLINEDITKMT
KSRVSALSVVARLMGVDMLPLDGNPVGQQVDKMKDNPKINYLKQKLKKSGSSSGEELFPSET
SLHTAYDSCQYMSTRDDDEWNDNIKLNENRRREHPQEE
ELQKFKRDFSAWQSTRFKECAKVVETGSKTSQLMVPVNLTSEKVVPFTNSEGMIKASKNLVEL
ENCGKVRQLKSGDFQNHSVKKSPSEKWDPLSSNKRRP
SGACEWSSNPKLDNSSVPMNIVILRPGVDSIYDCEEYSDRSSRNTEERDNMEDFLEEVKERLK
HELQGKANNRTTDSGGLIETSNSEKPSEPKKIAQRIA
KQVRESVAKDLGVNLIRSESTRSYRSEVQYNRIGSPEFISRNARNFLTERLRNVLKGETRPNVS
MVDHNSSSLSMLTYDRDGLQNSQETLNSENQMTYWG
RVKIEQDKQSRSFRHEPVDDIVIHKDLSPRNLIRSLSAPVSGTSFGKLLLEDRHILTGAQIRRKHE
VIERSTVKVKKQKKEKFNLKDKVSSFKHGFSMRG
RLFSRKIQSPEKFTNAPSFLNDITSGPTVMMNFCDRHENSTEVPPSPASICSSGFEESWRPAEE
YCSPTPSDLHPSEENMMPLAFREISSNLIELRRKLN
QLDASDSEQMMTEEPQSEDVMAELDDQSEAFVRDLLVASGLYDGSTGKSLSRWDPFAKPISD
SIYEEVEDAYLKRNKNNEEAMHQLGKEVNHRLLLDLLN
ETLSTIVEPPITMTSFRKNAIGSNLQSPRGRKLLGRVWDIMCVHIYPPADTSFYSLEGMVARDLQ
LTPWSVLVNDNVDFVGKAIECWVVGDLIDEIVKDM
GLTV

>DCAR_005887 (DCARv2_Chr2:19337482..19338982)
MKFSYNSSPSSASYTTTNYDESGCQSKPATAGCIHGIIRRFLCFNTSIPTHPSDHFNAEAATAC
RSLDDASSGIGEASAATPGLVARLM
GLDSMPMSDHRDFNSVRRTKSMDSLRDVELVQSKHRHVKSTLSFRDGPSFLELEDEEFFILSF
ESGGKNKDLRSNSRKSDLGFSEMRTKRSEIKHGDKNR
SRQRAENCDKENQGTNQVSDKNSRHPRSVLCAIKNCHENLRSPETVKQQIKKAINNEEPKGG
RLRKKKKDQKCSQVKNVETECDSENSSPVSVLEFSELP
IEFGVPSAVHKAKLTSSNSRRILFEELKNYKSNPSVIIDDQEAKKQRDTCPEIWKRLIKHSHNYAE
IWEEERKFAETDMKQSKWMYRGVLDNEESDEITA
TFEFEILDQLLQELVDQVFSL

>DCAR_005973 (DCARv2_Chr2:20475779..20479186)
MGKHFPAYRNNDQSEGIFPSCMWGIFYALDYHQWHNNVRKMLPHRRHNKRKNVKCNKNPKT
ISSVNGAAQKLMDAKASHFLVNPATTKI
SSTYKRSLKARIKALVAEKIRKADDNKKRGPNFPALPQLERTLSVKHVESPKNSFGKASNEWKS
PIIFFPRKTAPGATKLQHPARMTASDWKKFNMCTKE

NLMDYIEHQKLSQNYTFTTTRETSADQARANLLMQNASRRHLKKYVDVLELFQVNEDLFQEFQ
QQRDVGKKDRLHMPNDSTAKARLTKSGTFPVANVSYM
RRRNFKPTTLKDKQTEHWSFPGGVKLGNGHQDPEVDSKVVAHSKDERQRLDPQNRVDKHEL
SCTSNSLIDQVTDGNEPLRTGKQRSRIYDTVIVDDDTKD
LIKSDLTIDGSGYNRRNSNSGHRRTSSLNDSMHRYVRLCDYSFHIKSKSDLPKSVTWTNEYDLL
ATRSTPISFRRNHSLPHCTISWPLQDEEFREILNPS
VPSMTAMVVTTATEDDGLSESKLVGLPGKEYPENFPLSDAMEENLHGETIVENSKSSPEVEILD
SLTMGVGDCGSAKVDRHHEESDELTDEKSSSNDDLQ
VSCLKIDDKKVAETSQAVLIRGRCLQQESLTPANSAFEGCESAYNYSHERESPLHSFNSLDADY
VPQSCSSKMYTGKDLKSFRTKNHIHSRLHSDDDADL
SYAREILKVAGFNENGFHGEWYSSEQPLSPLIFNEVEESWWPHESECSQANLILLYHHQLLFDL
INEVILQIYETSFTYYPRALSTSCQVHPLHSTSHDD
EVLKSLTKWIVLKPELDQQLDDPVPRDLAKADGWMNLQMYSECVVLELEDFIFDELLEELIYA

>DCAR_005334 (DCARv2_Chr2:11916131..11919541)
MGKQYPAYRNNDQSRGILWRIICALDYHHWQNNVRKMLPHKRHNKQNNAKNSKIPKTTPGAD
CAGEKLVGAETSHSLVNPRTEASPTHK
RSLKARVKALVAKMIRKEDDNKRTGLNFPALLQLERTLSVKHLDTSNDSFRKTGKDWRSPIIFFP
RKDGATKLQRPAMMMASDWNKFDLYTKESLMDYIE
HQRLSQNYSFSGAREISVDQAHATQFIQNASQQQLKEHADVLKLLQINEDLFQEYQQEVDLCK
ENDFWMPKNSNAKARLTKSGTFPAANLSYLRRKNYKP
ATLEDKQTETWSSSRGKNLHNETPQTVGSEVFAHMEDQRQKLKLQNNTDKDEVPCDKADRN
KLPCSSASLLDQVSNEYLPSMREKQLTRICDTRTVRDDD
FKDLNSCNITFGPINNLRSTLRHRRTSSLNDSLHRYARLFDYSFSMTNKLDISKSVRFTNEYDTS
STGNAPISFKRNQSLPHGKSPWPNHDEDSLETLYP
YVSSMHSLVCTTATEDNSLSESKPVEQSVGKNSEKCLRLVKNEGNLHSETSVEDVKSNQEEKY
LESSTRVIDVCGTTALMNGHCEEIVGPAFEKSSCYKD
LQVNSMEIDDDKDTRTSLMDSTLISCLQEDVASPTELSVSEGLEHTCDHSYERESLLTLLNNSD
KDSVSMTSTACPENLNNTRKNFKSHMDTKLDKDEES
DLSYVRDILTVANFNDKGFHGEWYSSEQPISPLIFDEVEESWWPHESECSQENLILLYHHQLLF
DLINESIIQIYETAFTYYPRQLSTSCQVHSLREPSN
EEEVLKNLLKYIGFKSELDQPPDDVVERDLSKADGWMNLQTDSECVALELEDLIFDELLEELICT

>DCAR_008398 (DCARv2_Chr2:41829923..41832499)
MQKRREHVVVTEMVQVGCKWKLKGFFAHRHCRPKKNLLRKNQNQEVTGAEHQRGKSSALG
SNLQGIKSAPKNKGEGIDDEINVPKLIQE
SDDMHKFALEILDSNNELFLKVLKDPNSLLVKRIRNARNLQQAKKETVKLLYENKLVECGNNAISI
KSQKPTHHTNKSLLERIKLQYGCPSTDAEVPSTS
NSIVLLKPRQTKHERDIFRKHRNLESSNRATGGKKLHLPTVSSFNQRDIEAKKHLSEMLRNAEN
VEHYTDKQGPRTLKSILSSPLSSPVHEYSTASNQEK
GMDASVCPQRIQFSSCSNYEFARDDNSSPLQNSEVAASSTLMKLTGDSPIVRFTKEITEPVYSS
DGLSYEAYANDMEMNDTIISEGLDISEAACAEQSST
FFECPIQDFWLENKPLNSSSVVFSSNPWTEREEHPSPNSVLEALFTKDFTGLSSNTNCSAISHT
RPHHTDGKEASLVKDHIRNLLQTTELHWEELSKKSH
PSIQLLNISSLVEHTLFLDFIKDILLELHQCHFDFYPRVPSIRQNTLASLVLAKNMMEEFMEEIQW
YLVPPAMPRTLEDLAIKDTKKDEIWSGRKLETEE
VVTQLTDCMLEELIMQTTYVGEK

>DCAR_006930 (DCARv2_Chr2:30045024..30048273)
MPANILTDPEENGLEKIGPPNPKTSVKELMEEAMSGENTLKIKSGSSDVTLDQSNPEHGGHPR

GNSKRVKNHRSKSCDVECIKDWGAAE
NSDQVLDQETLKNLDMGIILEEFCRLNPKGSYLKKDSQDAVSMKSDQVRTQAEEKLAAAIKVFT
SQRFGNDEHLTKDKRTHYSKELMDALQTLSLNKELL
FELLQDPNSLLVKHIQSMEDGQYDKDQKTSSFSNQLEEELSRSTPGHKNRRIFRRRSKSQDSN
FLKDYDKSQPSSRIVILKPGPTTTRNSGTDRTVSTSL
QSIDDRSHSERNYYHFSLNEIKRKLKHAMGKDRQGLSFNDTLPRHFPNLRNNEKGIGEGHGG
WSSPNRDHFYTEKFAKSPLGTRRGDKIGNLKESQRAPE
NETIEITSQGVSSIYFEAKKHLLEMLSDGDEKEELVERQLPKSLGRILSLPEYSSTPIGSPGKERE
RSIVIPQKKSSPSSNYHTVDENMWRVIQENHLIQ
LSSPKKNLGKPSIIVNDEADNKLQFPELNSDGQHDADRDTLAQEPMSTMKEETICEETNCKAVD
QAISEIYAETLESVRFTNPDAQECVQISAVSRESSS
SLKREVRSCDAAKVCDDEKHSASDLSKGTDALSSPPRSPPSPSITQKVGTPGMCIDREERPSPI
SVLEPLFSEDDISPSRTKVKPAVQPLQIQYEELVSS
DPDHTMCTRTCMNDEESAFEYVEAVLFGSDLNWDEYLSRWLSSDQVLDPALFDDVELFSSRS
SHDQKLLFDCTNDVLKELCDRYFAHSCVKQNVRIIPRG
MKLINEVWQGVERHINQPPAPRSLDQLVRTDMSKPGTWMDLHVETDNIVIELENFIIEELMEDTI
LSLANNRIKDVRCETIEDEEAVYT

>DCAR_005854 (DCARv2_Chr2:18964967..18967777)
MTFPIPGAEMPPKSPIAMPLFDVSEGVRNSWKFSPRLSLDSRATFDASKRSLYEKQERMNSSN
ISVIRRDQSDCGAENGGRRSTSVIAK
LMGIEPLPNLPSQPVARKAELRRSVSECRVSCDMFQSRMVEKKCKDDAKVKQQNHLITSNVIR
EKEVDQSRRGLKSPQQCKSFFNSADFFPEPTQNVSIY
GEIERRLKMKGIDEPSKDLETLKQILEAMQLKGLLHSNQTPQIKNKNIVYDRNYSQCSETQSPIV
LMKPMNGRVSDDSGSNSGNRVSSISPRRQRQSIDQ
NVSSPVRARNLSSPPRTETNVRSSNSRSNSLVKTKQLSIETQRRGNESIDSTKVSPRISPRRNA
ADQVVINQSPINRKPIENEFSSILSESGVSKPFQTD
TERFKESEYKEGRRLLNRCDKLLNSIAEMNATESQPSPVSVLDSSYYQDDSSLSPSPVIKRSISF
PVELEELGSPRTSSVLLDCGDEINDSDFIYISKVI
EAWNSAPEETKDLIFLQLEKQHYMQTKDTSKVSKLQRRLIFDTITEILEQKRNFPPWKALSIKNR
NTSRPLVQQIWSECQKIREQQLSDDLFEVICGILE
KDLAGDSVVGWGDFPSEMSEAVLDIERLIFKDLVVESIRDLADSATKSSYLAPRRKLVF

>DCAR_008585 (DCARv2_Chr2:43316019..43317136)
MTSQLVPIGQKPRMLKDFLADNSHSKRVSFFQDNLNMTFSNSGFSKFLIKYLPFTTNIKPPSILP
RSITKRLSKSRSVSRNVNMKELTA
KVKVKDILRWRSFRDLAEVESQPLNFSSSPHHCTTVTTTTGSTSMTSNSGRSSWCDSDFTAED
LPSWCSNSVTPKGKWLCDEEKEQNSPVSVLDCFSEED
EESFSYFHQTPRRKQAGTTELFVNKTHKHEEPENRAEEKAMQLLNQVKTNISGYLEVEEEFLLL
DFFSEQLTENKKGDYELTKVANEWIKGEDEGCLEWK
LVGKKEFSIRDMERGVKWNKFDEDEQELGLEIEKQLLNCLVDEVLFDFLDL

>DCAR_006904 (DCARv2_Chr2:29871621..29872631)
MASAQLVPMGQKPKMLKDFLADNSHSKRVSFFEDNLNMTFSNKAFSKFLIKYLPFTSNIKPPSIL
PRSITRRLSKSRAVQPKLNAKDLA
VKVKVKDILRWRSFRDLAAEQSTPLDFSSSPQHCTTVTTTTGSTSTRSSSGRSSWCDSDFTAE
DLPSWCSNSIEQLGENVCDEDKEQNSPVCVLDYASQE
DKDSFSYFHQTPARSQTRTELSANKIITDEEEAMQLLNVVKTSMSSSAECLEIDEEFLLLDFFMQ
QLSDDKKGGDELIEVAKEWIRGENDGCLEWSTVGK
KEFSIKDMERGVKWDKFDEDQKELGLELENQMLNQLVDELLSDLLHL

>DCAR_006762 (DCARv2_Chr2:28397460..28401044)
MLQRASTAVYKAVKKLPFASSVKPRRAILPRSISRKLFKRSFWKKSDRKEIQRWNTFHTVVDTK
SEPPIYSPVQTNTVITNSKKVKSYS
SSTVSWTESEFNSSSSCSSGNSGTVNSSENDGVKNSEQGVNIGKRVDVIVGEAMTTSDANCA
VDSPKKPWPSEENKEQFSPVSVLDCPFDNDDAAEEEDE
VSSAFKHKRAPPVEGSKHKLKKTPRRLESLSKLEPVALEKRIALADCENESVGSPLLSIHENLVS
DNDEDYTNSGKKSEELLEQMKARTPSMSLRLNSDD
NLFLDFFIEATCEDNNVSDCELLDMAKDWMNGGPQEVLLGWEVQKNREVYIREMEKGGMWG
KLDQDRQEVALELEAEIFTSLVNDLIVSF

>DCAR_009231 (DCARv2_Chr3:5655351..5658971)
MGRDSRVHLKHETRNCMVSILYAIRAKKKLPRKRLSAGRKQTAASEDDEVSADTGEAREHSDT
GAEKCLISEKPTELAAGRQDKETKKN
LETKSSITSRIKAMIYEERSKKGGSHRRSDSCPSWAQPKGTDFSTHSESTGFNFELDMEDEGK
KSAGENHCGSSTSGPHLTDTTDKSANKNKMCELCAAK
LSRSVWKQSEANQEVRHSIGSNSFLEDDIIDSEKIGEDASVQDLNELLDSLDVYGPKKEYFLKVL
EDPGTPLLQYLHSREAIKSKVVLTKSASFPLPGSV
GRRVLESTNNDSKQRSALDANERKKFQVCHSSQCSADAKLAGTMVERKEVGTSEPILGRNRS
FNGLPPCTPRGLKIEHENRPVINRFKQLKRKISRVIRE
SRKEKQQPVMDSVSQIVPYDRTTSKVMQEETDFTRDSGSSSCEVDHSTSFLQKPSDKPRFRR
ATTFTDSMDRYNRLFECTLKRMARYQLAEKSTLRAENV
PTPPGKSSSKMLERLSSLPNLRDHSTLLMDSYPSPEGEPHKNLEDHSGMTETEILDEQKPLDC
SVDQESQKQLDTAEERAPVNDLEECSESPAQSENLNA
DEITSYCQDNLESTSVPPHSEPSPPDVSDTNSEKITASPPQLSMVEADSLASKDKEIIMVDNAVD
IPEVESPLKQWCRKLLHVQVDTKHVCEFNYVKDIL
ELSGFTADKFLGTWHSTGQPVDPSVIEKVEGFPLTELDCSEDEVRSCNRLLMFDLINEVLIEIHT
RSSSYFPKPLTCRSHISPMPVQYHVLGEVWTNVKW
YLSWRPESYRSLDDAVGRDLDRGDGWMNLQFDAECAGLEIEDSLFDDLIDEMLYDDLLED

>DCAR_012619 (DCARv2_Chr3:49441818..49445636)
MNGIQNGGPHNLEEPLPGCFGKMDNLFDLTAGMPGNQLLTEKPYRDESPTNVVARLMGLDAI
SCQQPHSASQRSHSRNSSYSNVPMEYC
QQVHDSFETQKLHEIHPYPVHNNFKDVYEIWQSHDICVKDKSSQRERFSGSASEKKMALIRQK
FADLKRLASDEKLRQTKQFQDALEVLSSNNDLFLRFL
QEPNSMFSKQLCDLHTIAPSPSTKRITVLRPAKMVDGNKFTGLQKQFQVKKTSQTEQVRSRDK
RTCGFSSPTNCKAEDSHIQTTRIVVLKPSLAKSRDLK
TVVLPHSSFPSALSTKDDQGEHENNDARESSVGKEITRKVCENFGGHSRDGTLRAPMLSNGY
SYMGDGKSFHSSENEYAVENLSDPELMSQASRHSWDYK
NRSDSPYSLSFNRASYSPESSVCREAKKRLSERWTMMAASGNCQEKGHMRKSSSTLGDMLAI
SDMKKLLRPEGDSSHKQEARASTSCLCTNLSDNDSCNA
TARNFLRSKSLPVSSTIDGSRLGLEVSNPKMEKNEIMKEVPKARAVKLKLKQRVSSLFFSRHKK
YGKEESSQPSSVSDHGCNPSTEAPEHSNDIASSSKV
PSLDLSSMEPILETSPTKAVFSVLKSSANENFNENQEQPSPISVLEPSFDENDHGIPEFYNNLKP
VGNGTRECSSHLTKSNLIDKSPPIGSIARTLSWDD
SCTETTTPFSMNMTAFPICADKERQEWFFLVQTLLSAAGFDNVEQPDTHFPRWHSHDSPLDLS
LRDQYLDLNDKEVQHEAKLRQRRSIQKLVFDCVNAAL
KNLAVYESTTGKKGVSCNRSNASLKDHASSTMVEIVWSQIEEWFSDEMKCVPGYFGDNRLVV
EMVARKEVAGKGWLEHLAVDMEDLRKEIENKLLEELVQ
EAVEDLTVLL

>DCAR_010159 (DCARv2_Chr3:15679329..15684310)
MAAKLLHSLTEENPDLSNQIGCMTGIIQLFDRQNLVTGRRITGHPRRLPPGDYSPTETNLNHTS
HRHPVGECQPNKNIHDKRRQSAETS
RPSFSSSSRSSSFSSLDFTRTAQPESLYFDSMISSEVPSKELTMRQLCASPHSERQNVNLRDV
VRDCMYRELREMSVKTTTKEATSEFLVGRKDLPRPLN
SSKVNDAIYRNCRNERQNLSVDLEESLKDFAEARDIRPWYLDEPSILSRSSSYQLRDGSLYSTP
KDAPRYSYDERGRIHSSYGSHDSVKQTLKLKELPRL
SLDSRESSMRSFNSDTQSSLSKSMQKDSGRLNEHLLPGLHQTYKSQQRPPSVVAKLMGLESL
PNSASNTEYMSGYDPVKEFEASSRSSKVTDVYRPIQAF
DSSRNARKEPTSPRWRNPDPGMKPISRASVEPAPWRLLDGTRGSQKVVPRNSKSLQRAPSP
FPSVYSEIENRLKDLEFRQSGKDLRALKQILESMQVKGQ
VNTSKEGHGSKFPNMQDHEHLTKGMNGKQKRNDQVHSHTSQRSNSSRISESPIVIMKPAKSV
KKPANRASDIPLDRLSNESRRHGFDFSDKKNGLARTAK
DQIPKSDRREHIEASNDMKSNFRTPRTQKSTRQQQLPAESTASSVKCSGSISPRLQQKRLELE
KRSRPPISPSDSSKSRRQQSDKQQPELSSPGGRCRPK
SSNLQPCDDQISEISSESKNSNFGDNDNSLVSDEVNSSVRSGEINGNKSPLRQTSKYTQGLVA
KKRILVRNDDESVAELGTASPEYPSPISVLDGAVYSD
SAPSPSNQKLETQDNSVWNSNDKSAKEQRHAYDSNIPTTVESGFKSEVSRKKLQSIDQLVQKL
RRLNSGHDEARTDYIASLCENTNPDDRYISEILLASG
LLLRDLGSNLTTFQFHQSGDPINPELFLVLEQTKASNLHKESYRAGKIVQSKSDNEKCHRKLIFD
AVNEVLNVKLACLGHPTEPWLKPLKVTRKTINAQK
LLRELCFDIEQLQAKKPGCSLEDENDGLKSISWEDVLHRSESFTDFQREISGLVLDIERSLFKDL
VGEIVAGESSNLRQKASRPGRQLFVSR

>DCAR_011036 (DCARv2_Chr3:31601773..31606638)
MSTKLLRSLTDEKADMQKQIGCMNGIFQLFGRANSHNHKRLPPGTCRSSEPEIEQNYTMQKSL
KSVPTQFRKEKKLMTNVELSRTSCSSSCSSSTFSSVDYNKTSQSEPSSSCHSNLPEMTSVVSP
PKQPNSSLQLSRQSVDLRDIVKESMYKQSPGLSVKTNTTEEKAYEAIKQKDSPRPQQQHKHVQ
AEVDRRDVSLQVLSKLRDGPWNANKERFSALPLVPRDSPRYSYDETESRRKLKSTTKLKELPR
LSLDSRERSPRISSSECRKNQMPRKDEESRVHGQIITENQEPGSNKKPSAIVARLMGFEPIPDT
APTEGQAIEVKSCLLEESETISPSLKVLARSRQSTGSGSPKVHCNTSVSPRLKNDSVYMDSISG
SMFSVKKAPRTQIDSNQGSRRQSMNCQETSTKLPLASSSVYSEIKKKLGELEFRNDGKDLRAL
KQIIEAMERKKESLDSKKNEEQNPNSESCTSQSSSNNSSNQAPAQRRRCNSPVSPVKGSTSP
KNRGTQIVKARSAKLIENSVIPDDDKSCLQKIWSGENAENIKNFAERRTAREFSLRNNCRRDSS
GPLPYIEEKKIDEPVNSLQHLKASQQTGRKNLANFRKSSPNLNPRMPQKKHGMEKLSGFATQS
SDLKISRIQSSKLPTEPSSPTKRQCQNSAIQHKRDDQIRRSPTGPRNMNKGDSRGSMQTQNAF
RLDVQIDRKSKSKSTYCTTEIISPYKPKDPNKGDAIAKYAGERLITELPAPLEQPSPISVLDITFYE
DDNPSPVKKISTVFQDNDNVNGDESEWISGHIDCLQSSRASKFDQRTLNDVSHLVQELGLLNT
SQDKAATDHHKSLTNPDHRYVAEILTSTGLLRDLGSSTTMTGIQINQAGLLVNPELYHVLEKDTE
VALEQVDKKSVRSTGREKVQRRLVFDAVSEILLEGSFDLRTVQTKMGERRTSGEKLLEELCTKL
DQQQATPDDSLNDIDDELKSILRADIMNKRKNWTGYSSQLAGLVLDIERMIFKDLIGEIVREG

>DCAR_012508 (DCARv2_Chr3:47684442..47687893)
MGCFTGFRQIFDRHQLLTGKRLYTTKRLPPPPPPPTRDGISPETEKPEKSLKETERPSPRTMAS
PSPDRYKSPELRSPAPVQTPPRSSL
PHPIFELNEATKYSWKFAKETPRLSLDSRATFDSTKGSLRTNTNKSTEDNSRRSTSVIARLMGL
ESTPPEAVTKPAELTRSASESRVSRELNNNFQPNHV
IRSNVIREKVPDPIRKPQHTRTESRAFKSPQQRRSFFDSADIFPEPKQTTVSIYGEIERRMKLKG
LDEQSQDLETLKQILEAMQLKGLLHSKKAQTPVKN

KNVVYERTHSFPCDDNEHQSFSSVVSVNRRGGSNSPTSSHRSKSEYRRSDSIPAVSPRRERV
SVDRSIKSPTRGRNASSPTRSELSARNSNVRRNQLISE
RRAVESPEHRRISPINSPRIIPRRNSPDVANRSPRNKKSTPQICTKEKISTTFVLEDESSYSSTIS
ETDTERCKSEDYKEGKSLLERCDKLLHSIAEMNS
TESQPSPISVLDSSFYKDESLSPSPVMKRTISFTDSSVELTDEELGSPGISSVNSKFEDEIDDND
YIYISRILRASSYLTEDDDIFLFVEKQHYFKGKDY
SKEARLHRKLIFDVAKEIIDQNKQLPPWKTFSPKNSNISKPLPRQIWSELQRIQEPTDQSPDLFGI
VCGVLKKDLAADGWGDLSVKMSDAVLDIERLIFK
DLIGESIRDLAEFAGNSRFLAPRRKLVF

>DCAR_009410 (DCARv2_Chr3:7289358..7291791)
MPDENNTSVPLKGAEALRNSLDIHDGTNYMEAATLSSANLKKEGTLHVETDMQIRTSGHARSS
FRRDDDNSTICSDSPGGTKTPTLVAR
LMGLDLLPDSSSPRVSSSSSTINHSLQNIVLNKKKATRSSFSNASDLISHSVPVTPRTSSSSSAR
RSDIDQRFSLQINKENIDVGSCQELEFSRFLKSKI
NAARMHEDKNMSPGHYARQIVKQFKESVSRRVGKDITNTSNHDYQRRDQNVVLLKPKKPSNL
GNGSETTTKSPRLRLSETKSRPTVTKVHQDFQHSPKLL
SSSQLCADKQSKHETVSLRSKPQKAVLILREEKEKQKSSVKKSKLVVHGDENMKCSSKFKKPP
QTWDLMRNKQEEAFVRSAALTNARASQTSDNKCKKTP
LPDLLSLKKKELKRQKKFCDSEASDPPSAKTNTKLLSCNLSQSCQQDPNDQNSLMAAEQLTDY
ISRILNCTGIFPFTPISIFKFNSLLHPLHPSVFHQLP
KLPNGKLVFELVDQLLAGILQSHFGERFMFGDELKCYTRCTKLEHTGKVRLDREQLLNTLFNKI
KNFRSVNCLVQEDIDELIDKDLPPKLLEFEEEVEGI
VMEIEDYILERLVHETVTVC

>DCAR_008759 (DCARv2_Chr3:994726..999051)
MAMKSDFAQKLLHDLRLKKERMAASQSSGTSSTMARDGQRNSGQTYRGTQKTKGPEPVRPN
TGNSRRRASGNARLPTDSENSSQLVQYR
SGQSTGQRVDLPMAILLALQNSGKHGKGNFSGGNPMLQFLQQIGKKSLDTGKMAGYGSLSKH
HSSTSQFPTFSNLHVNEISKGVQNLNHILRACSNGLNI
DKYSIEVGKELLKGATDLEESLRMLVNLQEASEYMIKPQRKNRITLLEEEDSDEASKGSTIQQKQ
LDLPRFSFDKPSRNSHGFVTKSDSNQRLIELTYHA
RSSSFSNNQASSSNSEPHRRSSSWSSNFKTPTDSEQTSHSNSSRAAAEKGRMSSIIAKLMGLE
EIPEQVPSQIRQKDSGTRNGEGMGLKITYLTTKNTEI
KTKKSDRQENITKKYNVLKQDRHVSETKNNNVPQKAEKHQVLHQLNSVVAIADENLQRKNHER
AKRTIHGSDPENSAIKLDKQQKSINRSIEVFGNQVLE
KEARQNSMKHRQLKTPEKSETKEPIIKDEMHQRLKLKHRSSSILQEKAEHRKVTAQTEGRNSE
RLTARYQPKPLQNNELQQLHMLRKSGIQEEKHQTNKR
EEQNIRSNYQAPTPNGSPLRSATAQDGSPVRSKVPLKTMHNENSELAPQIQTEKDMMLPLERK
RPIHVLATGKKAEITQKFRGQIPRKMNERMNKRSGTL
TNLATPTKHQTSVTQESRERIQETATETKGSEEHSSVACKEPEESTEEAKSEEIVQTLSTDEEK
HFETAATIDPSSSQDDECRSLEVVSPPDANDISKDT
AHQAQPSSEQKSLETLPSSLIGSNEISHPRLQEHKKALISWKQEPLTENEKKLKETLITSPLFLNT
AEALFRLNIPVGILHASDQIYQEKSTKFTIDCAY
EVMNRKGRRQELLQQCFKVSICNTTVSSLDNLIKQLYKDFDRLKYYGGNEHDDNDDAEALLKM
LEMDIHNTHPHLNCMWDFGWNEVMFAFDGTNDIIKDV
ERQVLDELIDEITSE

>DCAR_012067 (DCARv2_Chr3:43482340..43485726)
MPPENLRSIAYRSFVTCDDPKGVVNCGIRRHKSSKKLEEKLEQPKMLKKVNSTLRYEEKKEVV
SKGGMEESHRSSSFQLMEVSRGAQKL

NQVIDSWSRRASFDGGSKDYAKDLLLGALDLQDSLAMLGELQEASQYRTNLKKKEKEKPCVSK
VDEVGIARTSSDRYGNPKYQNPRPSGDGSSRDCYDEL
REVIRDSLARQNLLPTQSSRESARQNLSTPQSFRENAYFDRKKMNSYGDIPSTSSSSISSMTYS
HDFASSVSTSSSKIPEGKPRTSNLVAKLMGLEDISS
KPLQPALQKHLQRESVLSQTPIFDVDMNKERKPQFVGQKMDRSMTLEEIIDNMQFQGLLKHNQ
HQAYHQNTSFKERRVSYDASPIVLIKPTYPGVEVKKH
SPHRFIPDEAALNSEIKQRFWKTEEEITSKIKEYPRRPSNSNESRRKLHSGDSVVKKLSPEKGA
KTSRNVLAKPRDVEVISEKKVSSNKINASVPISPSP
PKEIVVKKIDKIRKATSKKKLPEIEDVISKSVPESHGHDSVPTMKLRKSETGSSNLSKNSGTQRK
GSGLNSPIKHTKSTSSCGDSVQKRTVKNSKPVKEP
VTANRSIPKAEKLQKSMQVPSIVHNDDNNMAHIKEKLSSKMVDSEPDVQLVLEEEMDDSEIPKK
ENCDSNLDKFCEDSAHPTTLLCEDSAHPTLLENGTD
SAHPTQLINGTIRHDEAPEQSKHDSKESDVFQPETTKATPETLQLVEEPSENFDASQQIVSSTLT
GVYDSAPLDSKLLVDYASELLEIKNHKLLMNPFLN
LANDSRLCMSNNNLLEEARIGMENLRSYGQPGLNSAPASDIVFSVLERDLRCKGVAADGPWNL
GWREGFTNNQVEQIVSDLEKLVFRELIDEVLADSVV

>DCAR_013367 (DCARv2_Chr4:30687977..30690663)
MAHHHPDHKHKHLRELLREDQEPFLLHKHIADKRCQLKTTSLQLRKHNLFINQTSSSSKHVSFL
CRKACFLSFTDSPDFKKSPFPSPAK
SPCARFLHIPAKTAAMLLEAAMKIQSTKPKAQAQHIGFNLFGSFFKRLRNKNKARKREICVNDAS
TPSVEANAVVSSSKNIEEVSEDTRRVSSAGWSESN
EGKSLDMESCSTTSRSDCSSPTSSPFHFALQRTLTPDILSPVTSPVSHKKDNNHEEESLQKIPV
GEEERDQCSPVSVLDPPFEDDDELNREEEDEEEDSD
FDVESSYAIVQRAKLQLLHKLRRFERLAELDPVELEKRMLEDVEEADDDDNEEEFDYVNDLGS
SLDIQENLDEVVREVLSRSNQYNQNKISSDVRRLLLD
LIAEERKSEPDNTSEALVSSVCKRLNAWKEVESNTIDMMVELDFRKELDGWTRYEEEVRDRAR
DIELAIFELLVEELVSY

>DCAR_015992 (DCARv2_Chr4:2853758..2857473)
MEKDEAGCMRGLIRMFDIRDGRSTRKFLTDRKRGSRNPAGTDTKLKMLTYKDDKDPASEDFE
ETKTDDINTSVKKLIEGGMFDAKGLKK
NINIAGAQLHELNSENGGHLSDKPKHPKRNHTKSCDMHANDLDVAARSSHQLSNQEKPYNHD
LEVMLEELFQLNQKGSSCEHDWHDSRSNQVYSLAQEDI
GAAFKVLISQRFGDNGHLGGDCNILHFKEFIDALEILGVSKELFSEHLHDPNSMLAKYIQRIETGH
LTTDPHTNPLFESSLLDQESNKSSLKKLVDQKHH
SFFRRRKRKGKSQNDDSSKYVDEFQPSSKIVILKPESTALQNSDTKSLGTASPESQWTVADITK
NERNYSQFSLREMKKKLKHVMSIEWHGVSRSSKSHR
EFQNLKNHEKGVVEGNSGWSSPNRNHFFTERFAKSLLGMKKDHKLNRPGESETTGVNEHDG
NSKQAIPKIYNEAKKHLSEMLSNGDEKEDRVRKQLPKTL
AKILSLPEYSSTPSSSPGNNGNHIFLTPQVKLFPPGKSDIVQENMPCILQENHVSQQLPPKQCLE
SGSCISVEETDKKVLSCIFDPYILGEQSNEKGVEG
TMSSTHDEEICEELPEIVTTESYLWEDIELEEDSCKSSSVPLIRAIEILEAAEISDKEKSASILTLNPI
RDEMLIHSPPVSPTSPITTVEIGSFDNCVDR
ADRPSPISILEPFDSDDDISPASSKLIPVKETIEPLHICFDEWDSSDVNHTVSIGTCMEDGESAFE
YVEAVLLGSGLNWDEYLLRWLLSDKILDPSLFDE
VELFSSRSNDQRLLFDCTDKALKETCERYFTRSFVRQGIYPVPNGMKLINEVWRGVEMEILQH
PASQSLEQLVRKDMPKFEKWMDLYFETDHIVIELEKF
IIDEMVEELILFSDDHATEDWSLVISHTLAKDEENPICQ

>DCAR_014029 (DCARv2_Chr4:25434499..25440017)
MAAKILQSLSEENPELSKQIGCMTGIFQLFDRQHLATGRRITGHPRRLPPGDSEYDYSTSGTNI
NKNNRYYRRPTVENQSNKHNIEKQR
LSTETSRPSFSSSSRSSSFSSLDFNRTAQPEPLSFDRMIFSEVPSKEQALSQSSASPHSERQVS
NLRDVVRDSMYRELREVSVKTNKKEAASDSLVKRRD
SPRPLHLSKSNDEFYRHGRDERHNMPVDLEEPLKFISEVRDARPWYLDEPSKLSRSSSYQFRD
GSSYLNPKDAPRYSYDERGKSHLSYYSQDSMNQTLKL
KELPRLSLDSRESSMRSFNSDTQSSFMSKGMQDSGNYTEQILLRQHQTFESQQRPPSVVAKL
MGLDSVPNSASNIDNKIGTNPVKESEASSSSSKATNAY
RSIQPFDYSRNSLKEPTSPRWRNPNPVKKPLSRVSVEPAPWHLLDGTRGSQKAVPRNLKALG
KAPSSFPSVYSEIENRLKDLEFRQSGKDLRALKQILQS
MQMKGLVDTSKEGHRDHEQMYTAPDQNTREMNQKQKQNDNVSSHTSQRSISLRTSESPIVIM
KPAKLVEKSGSRTSSAVPLGRLSIKSRRQGYDYLDKTI
GSVTSKMDKDQIYKSTHRDHTETSTNMKVNVRTPKTQISTRHQQVHTESTTSSVQSSASISPRL
QQKKHDLEKRSRPPMSPSNSSKLKKQQSNKQQRESS
SPGGRHRAKSSILQESDHQISENSTGARKSNYRVNINSLVSNEVNSAVRPSGISGNQSSLMQT
AKYSKSRVAKKSSVIRNDDDSVAELGTTSPEYPSPVS
VLDGAVYIDNESSRLNEILVTIKNNSLSNLSDNITKEQFHPVDNNVSRTEESVLAPHVNRQKLQSI
DHLVQKLRRLNSGHDEARTDYIASLCENTNPDDR
YISEILLASGLLLKDLGSNLTTFQFHQSGQPINPELFLVLEQTKASNRLKEDCRAEKVVQLKPDN
EKTHRKLIFDAVNEVLTTKLAFLRQSTEPWIQPLT
VTGKTLNAQKLLRELCFEIEQLQAKKQACSLEDEDDGLKSILWEDVLHPSESFTDFQGEISGLVL
DIERSLFKDLVGEIVTSEAANVRVKTTSRLCRQLF
VN

>DCAR_014830 (DCARv2_Chr4:18030671..18034734)
MDGIRDQRHNSTTPTNAAGGNQHISKDKGILKMTSPNVVARLMGLDVLPSPEVIHRPQKTLAG
NNRLKSSPRRSQSDDHLCEAQFYHQK
GSVEQPQFKDVFEDTSKKSGASKNPKVELHQQSDSLIPEHPCRHKSPTSYQISVLKPSNPGKQ
KTKAEGWKAERGSQVKDDFVSQQIHVDDFLTRTYGSP
GSQSSAKSSIIPADRKSESDIIPKRIVILKPNYVMAQNASNSFLSPDSSNFDFSDYSNHKDYASTL
MSEYYFGDMEIRKDVVNFRKPRSRKVTKRVMKAT
RDMRDTSSYMMNNRDTANKFGENTGDDIGVVINSIPSYVQGIADMRDLYDFSGNVFANESEG
MKSTSCDLRESFVTSEAKKRLLRRLEKAHKYKDARDNS
KESMLRDVGVNGKKITLGEMLSTRDAYRNLDVKMSSINRRDGWNDSYSKPSSSSRSVHQLPG
RTSEQQNADGEFHVDDKLLIPKNETNQYRRKGAKRYYK
KKDKSTIKNSRSSKSIAQSDHLRCAYNCESLLESCSSQVPMEINLNNKVSPDEQLLIPRTPSSAA
SIVDAENIQHRSGTQFLGASGELHPELSICMEKYD
YFFVADQENSTPQRTKAEAESAESSKEADNPSPVSVLEVTLREGDVLSCPESFDQVSADLQEL
QKQLQLLKRESTSYAGDPILNDCAAQQGPVTSFENVG
IHKHEYWESTYIIDVLTESGFHNTDTDTILSTCYSPDCPLGPWVFDNLENRLCKEVTGLKHARKL
LFDRINLALSEIPGSFMDPFPWVRQSSTGIGFRWQ
KCDINDKLHELLEGQEKDAYEDELEKLLDKEMNWVGSRDFVDAIGIEIEKVLTDELLTELVNEVA
FH

>DCAR_014604 (DCARv2_Chr4:20535428..20537920)
MGRDWVYNLSSHSGAESNRKRSRKRGGVDRNNSSTTTRSPSGCMNAVFQLFDFNHYFNFPF
NSSASSSSTTIDHHPPPLQGVVAPRNSL
EMEDTFNIKAAVLSSSSSVNNPQSLQLPMGGIQIKTRVSTDQDLSSSSESAYTSGSPAGTKTPT
LVARLMGLDLLPETSSPRPSSSSSTSNLSSSKSHLV
YSKNKRRLPRNITNEANTGTRSLPETPRTSASSARRSDVDQRFSLQSNKENSQLSGHEFEFSK

FLTAKINAAAGRQGEENMSPGHYAKQIVKQFKESVSR
RVGRDITNTVENRSDQRRDQHVVLLKSKKPTSSSHDQKNSCSPKLKLSDMKSKSMSSTHSPK
LCPLPLSPFADNQFESAKGPSSVKAQPVVVVEEKRIQQ
NVGKKVSNDQKYNSRLLVPQSSDLIRTKQEESFVKSSTSSRPKPSRHIRIPDQKCSRSTPLANV
PTILTIKRATRLPHQQEGQRQQHKSSESQTKNSSRN
GPPSPSCLSQISKCKQKGPKPTVPHLVTPCTNSSATTTGDHLRQYISKILERTGIHDSPAAVSIT
NWYSPSHPLDPSIFHHLRLSETSTLSQCQLVFDVA
DEILAGILRPYLNLKPWNNYQKGKIVMCLEYPLSGSQLINTVCGRIRSLPSADCRVLEDIDGLIGT
DLSGSRERDAYEEEFGDGIVGDVEGDILDGLMHE
TLHILLVSVRLKLE

>DCAR_017186 (DCARv2_Chr5:12549833..12553892)
MSDTGSALVVNEKRTQRTGGCVGIFFQLFDWRKRFAKKKFPPNKLLSLDSAQHGSKKFGGDD
KLPKLRLMSNEKNGTRNVDTGQKYAMR
TPTLVARLMGLDSLPAVQRGKIKKISSDRIEVDTGGKFASDCCQFGGQHKKFKPEGSKHELKP
HKLQKTGFSERRAVARFGAERLQFKNVLSRSNKHHHP
NFVPSVKSPTRLSKRNAARLIGAATRILEPGLQARNRAKSGLPYSSTTDGLLVEAGTGLSKDQV
ESATYYENVARFSRGKSSCENCGNIVDIPESGTYME
EQPSCQPSILVNGGCPLSGREICTQRLPLSYLGTEKGIENPKKITAFSAQSPGNVKPGAEDNFK
RETLHRGSQIRWQPNQRYRKEEIPSSSICYKQKFCV
RNLGVTGRDRTSPRCTDTVHLKKDISLNQSTIDHKCSRVPSKLDKCRYASKRRSTDRLYDPLSS
ARKRRSVDAARQDDIFSSFGSTIERKSKINCNVLSQ
TMVSGDIQSAKLACISSNLVSLEKINKTSDTNAGVLPFTFNSKKLKDRTLVKTNDGGFPSECTSS
CSKNSSVLDKININEQVLFQNSSPFTVDTLGVLEQ
KLKEIRSLNDNEMALGGAPLRQTPAIILQELISALTAEGSFNVNNVISMPTETGISSCCEHTISAHP
HLQAQQRKEAVQGRHLNVSTSNYMNPSCVLEAS
ISNGSFLSNSLDEGLVHKPQTDFKYLFHNEQVVESGADLLHGTTSTSIGRPDTELMANFVNSIS
DVLCSMDLVDSRLKGTKLAHAEEVMLNAELAFGNPF
PHNSNETKGFSICRFIVNELETLGSVLWTNFGCFSESEDTEGNLLKGFLFDCVIEYLDSRYISSIQ
QRINSLTNLPIPMNTEMLIREVVEEIRRWTSWSG
SVLDEQIEREMRSWTDFDVEAFETSVAIDGDILHSLVDEIVLDLWHIKLTSCI

>DCAR_018538 (DCARv2_Chr5:30939649..30943390)
MTGIQNGRTRNLEKPFPGCLGKMVNLFDLSAGMPGNRLLTEKPYRDGSPLSRSCSDVSGMSP
IQDLVEDKLIISELRRTSSSNKSNSKP
IKMLVAEQMAKEAASTDNAPNVVARLMGLDALPCQQPPALRRSHTRSSSQFDVPLNYWQQGH
QPLEAEERPEIRQHLEPNEFKDVYEIWQSHNIRVKDNS
SQRDRYGGGESDKKMDLVREKFTDLKRLGTDEKLRQSKKYQDALEVLSSNKDLFVKFLQEPN
YMFSQQLYDLQSIRPYSDTKRITVLRPAKIVDSEIVAG
SQKQFPMRKTSKTEQVNRDKRSGFSTPENCKFEDTRVQTTRIVVLKPSHEKSHDIKNVLLPCS
SLPSALSIEDHHGECEEDNAREFSKEITRQMHEDLAR
HRRDETLPSVFSTGYIGDESSFHKSEDEFAVENISEIELMSPTFRHSWDYINRTDSPYSLSSLSR
ASYSPESSVCREAKKRLSERWSMMASNGSCQEKRH
MWRSSSTLGEMLAISDMNKPPRPEEDSSHRQEGRGSTSSLITISNGKDNSDVSPRNFSRSKSV
PVSCTNYATRPDLEVSDPKMGRTEIIKEVTKAKSVKS
SLKGKVSSLFFSRNKRSSKEESSESNCATDSGLPKFTRIDSYASEQGSDIAPSNKASSSDGFS
MEPKSTPIPAKAGFSVSKSFKNDSCNENQEQPSPISV
LEPSFDEDDHSIPEFYNNLKPDSNGAELSSHLTKLNLIDKSPPIGSIARTLSWDDSCRETATPSS
LKSAPLPIGAEEEKEECFFLVQTLMSAAGLNNEVQ
SDKKFPRLYSLEGPLDPSVRENYIDLNDWETQHEVRRRPSRSTQKLVFDCVNSVLMDLASYES
ATCQGGKSRSGSPYKLQDHASSTIVDNVWDKIKDWFS

DEVRCVFGDYGYNSLVVERVVSKEVVGKGWVEHLTTEIDDIQKDIEKNLLEELVQEFVEEFTGG
L

>DCAR_017894 (DCARv2_Chr5:24613932..24617183)
MHILMLVSLILAGNHKDRGTSKLVSPNVVARLMGLDGLPSPEVSHISQKVPADNKGKIALPKISD
RDAHWRELRSYKKSYMEQSEFKDV
FEDTINKSSSNKNLGTQFLQQSDSLFLENLPMQKAPSSYEYNRIAVLKPSHSTNQKAKDKGWQ
DEREGPMKHGFVRHTCNNHGSQSLYKSSTISPDRAGE
SDIMAKRIVILKPNYVKAHNAFNPVSLSSPDSSQFNFPVYSEYEQSEDSYREGYYRNMDIFKDV
DFSRRKSREVTETVMKATREMRNTYSRMKNRRGTAK
ELNGETVEEFGYVMNNFPSPFRGSPDISGLYDFSGNVTASESEETKLTYDFTESFASSEAKKRL
LERLKKAYKYEDKRNMTRDVYQYAGMDGKKITLGEM
LSTSDKKVRNLDVTMGGISSKDGWKDSFSKYSSNSRSICQLPGVVSEKNNAGGECRVDDKLL
VPKEDTYQYQRKVVDKNCKKKENSSLNSLKSSKRRSLS
DRKKYAYNTDSSLEIYPSQDQTETNFNKEGSPDKQLGASQTSGNITSIADADIIQNGSRNLSPR
SSDEMPQEPSIFMKNSDDSSVGNQENSTPQRTKAEA
ESLESSKEADHPSPVSVLQVPLREDDALSGPESFEQVSADLRELQKQLQLLRRESRSDEGDST
PQDDDYDVQQGSVTDSDHRRENKPECWESSYIADVLI
ESGFADTDTDMFMTICYSTDCPLGPWVFDNLESKHYSEVNRLKHDRRLLFDRINIALSGMPKSF
AEPLPWVKPSAVGIRFKWQACEIRDELYKLLEVQVR
EASEEDSEKLLDKEMHWMGSRDCIDAIGVEIEKLLTDELLTELLNDVKFSN

>DCAR_021448 (DCARv2_Chr6:22864332..22868406)
MSEAGGSVLAITEKKAQRASGGGCGGIFFQLFDWNRRFAKKKLFSNKLLPLGSKKFGTDDKLP
KLRLMCDEKGEAFSNGKKNGARNVDS
GQKQGMKTPTLVARLMGLDSLPAVQRNKSKKGYELGVDRGEEIATDSCALARQQIEVEKAGS
KHESRPQKLQKTGGSERRSVSRFGAEGLQFKNVLSRSG
KHHHPKFVSPVKSPKSLSKKSASRLIGAATRILEPGLQARNRAICGLPSSSQQRHHNPTDAHLV
EAETAVSTDQAECSTYYESEVRLSGQSSCKIYGKSV
DFLESRSYLEEQRSVSDYMGCSSFGFESRQRLSPETGKESIFSANPVNSSDCTFQPPSNVNPR
AEYNINRAALRKGGQTRWQLTSQQYKRQEASPSSVCY
DQNIYVQDQGILRMDRIPPRSTYSVDLRKEPLNQRPPVRSSRMHGKLDKCGYVSERRSTDRR
YDPLSSARKRRSVNVAREGNVSGFAGSPVDKERYINSN
ASSQAFVRGDDKSTNLPCIDSNLARPGEIIRISDTSNGDVSFTFSSSKKNKERILAKPDDREYQS
ECTCTHSSQRSSAFDTINRKRQTCFQKLPSPGDTL
SVLLEQKLKELTSEEENAMPLEGSPLKRTSAIILQELISALTVERSFDANDVVARPTEIGDSSCCE
HTSGSHPSFQAKQKVTEAQERYSNDSKNNNMSPL
SVLEASISNESCLSSSIDGGSDFSYNEAQQLEPGVDGLHGVNSLSKERRSIELVADFLNSISDVV
CSMDLVDSRLKGSKLAHAEEVILNAELVFGNTFQH
NPAEIKGFSVCWFLVNELETLGSVLWTNFGCFSESQDTEGNLLKGFLFDCVIEYLDTKYVPSMK
GGIKSWRGLPLSMNNEMLIAEVVEEIRSWTSCTSFV
LDEVMDREMGKWTDFDIEAFETSVAVDEGILHSLLDEIVIDLCHRDQSLYV

>DCAR_020774 (DCARv2_Chr6:28467368..28472283)
MASQKHLRELLRQDQEPFHLHNYKPKNKPVKPVTKSASSKRGSLYKQACFTSFQDSPDYFNS
PLKKKPSATTVLHVPAKTAAGLLEAAA
KIQNRSSSKPKNARFAGLFFGSILRKIKDKNATTSKTRELGSSSINGPAFEAQRDQFVVDNSKD
KYEMMGYSFSECNYSNISRRISSAGWSESNTPEDDN
KSIDMDTISSCTTSRSDYYNHDFVLPDQPHFCLSPLSPFRFALHRSPSVGRRTPDFLSPATSPG
RHRAQEDDDQATENSEKINAQEEDEKDQFSPVSVLD
LPFEDDEREDEREEEEEEDEDDYDLECSYALMQKAKFQLLEKLRRFERLAELEPIELEKRMLEG

YEDEDDNDDYLDENEEHEEFGMNVDDLICEIFSTSG
PCNVEKVPGHMKRLVFDLITEEKKNGMDNRDDAMVKLVCKRLGSWKEVESNTIDMMVELDLK
REVDGWKNHEEEVGDRAKEIELAIFGLLMEEVSEELVRH

>DCAR_025439 (DCARv2_Chr7:27232257..27236706)
MSSKVLHSATDKGTDVQNQLGCMSGIFQLFGRQYFLTGRRVNGHSHKRLPSGQYVNHGMEK
SGTRQETPVNSILQQHKLLIRSSTSGIV
NLQMRLKQVGKEKLMAPVEPSRTSFSSSCSSSTFSSADWNKKAQSEPSSSCQSSCTKITSPPL
LTRQPSSSPHLLPQSLDIRDIVKDSMYRETNRLSVKT
TKEKVGVKAMKHIDSPRPFQQHKSVNTKLTRNDGSLRTVGKSRGAPRTAKEQKYSSPTLTPRD
APRYSYDERISQDKLKSTAKLKELPRLSLDSRERCIR
VSASQSRSNYQPIERGTENGISRQTTSLNQEPGSNKRPSAIVARLMGLEPTPDESQSLLKEDS
DAMSRTSRIYDKSKQNQVTGSPIVSRIPGSKDDNPLK
KSTSSSKSPLEPAPWKRTDGESLKIQEPLKKSSQASPSVYGEIQKRLTELEFKRSGKDLRALKQ
ILEAMEKKREILENKKEEQLTQFESHRSECSPSSSF
DRVSIQQRQTNNPTSPIVKGSSSPKRHGSQIVIMKPTTQIEKPATKTDCKPVFHKKWSGDNDHN
ILELVGRRKATHIALKRNHIRDASSWAQPPNIVKTK
VEILKSSQQQRVPQHIGGDNHASYAGNSRNAGQRLQKKKEIDKLTYSTTQSPDPGMSRKESF
MHPVQSSLPTRNFRAKPTYPQQDIDQLNRSSNESRNSS
HQGDTVSMQSESSSSPDSQIGTEKPGQNHLEQITDLFRPKDPNKENFTTRLAEDVLNAELAAV
TLEQPSPVSVLDITFYEEDFPSPVKKIPSAFQDYDTE
NSGEAERGAVYIDHIKNKKKSSCSYRFEQGKLENIDHLIHKLRLVNSQDEATKSCIESVCDDNNS
EHRYIAQILVAAGILMDRGSNLTAIQLHPSELPLS
PKLFCVLEKVMCNTKLVEENHEKERQSKFNKAIQRKLLFDVVNEILALKSTLTGSVKPWIHQNLR
AENSMRGDKLFKELCSKITHKQSATDSSVVGDYDS
ITTVLSADMINESGWAAYNFELPEIVLDIERMIFKDLIGEVLCGGTTGLRLRLQSGTHCRKLFPK

>DCAR_025910 (DCARv2_Chr7:31634058..31637198)
MSDTVSSLVAITEKKSHKPSSCTSVIFQLFHWNRRITKKKKMLTQAGAKQASKKFGSDDKLPRF
PLITDKSITVPDSGKKQDMLTPGLV
ARLMGLDSMPAVQKTSCSEIGSGLETVEDCLYGKHTENSETFSSKHELRPQKLQKTGSSGAG
KYAVTGFGAEAAPVKNILSRSKNYHQKFAPPVKSSMSL
SGRHASRLIDAATRILEPGLKSRNRAKSVLHDSSKKHHTFANTVNTEATRGSTDGLLEGSYVMN
DSKSVNRCHNPLNIVNSRQNLVGQPSVSYSVNHSSH
DFSRVRPRSPMSSFESEKEKVNQDNQEKITAADQAMHNASSYTGSISYRMPLHRNVRNRWQL
TSQQSKSQQDLNCFNSYKQKFPTQNQVNVVNLPRSNES
RDSVGLNRSSSGLAQQHLPTEVDIFEYDTERRARDKADSHVPSVRQKSLKVIRKGGGSGFQH
SGNQMHTKSDAVSGNGQSRVCRESKFTCKQESNKATGR
VSVQNDVISFTFSSPMKQKEIPEETSKMGDEVFCSNDVIEHIRSKKTLLLKGDTLGTLIEEKLQEL
TDQEIDEFASGSTPERTSSMILQELMSALDSETP
LYHNNLVVEPYGRNATSCSGHPPNLHARFQPKAKRIGDLQSYSNDSNYGVSPVSVLEISGLFS
STNYTSVHQHQDGLIELLCNKPQVLEPYYHPTISLSE
GRPGLPLVTDVFSDASELLSRVGNIGSRLGGTELDYAKEVILNAELLLGSSVQRDNDMNKGFSV
SRFLTDELDTLASVLCANSSFRFDNKRTSLQGFLFD
CIIEYLDSRYGPHLRYKNKTPRNLPSCINAEVLINEIVKEIMSWKALASLIPDGHIEKEMSSFLDRS
FETELFETGSAIDEDIFQILVDEIVIDFPLTIL
NFSY

>DCAR_024016 (DCARv2_Chr7:7948275..7949513)
MTRDSEDTASNKSCFSGILRRILCTGSLPTHPSDSDIVPGSDDLILEKKNIQVKGQGTPGVVARL
MGLESLPDIKWVPKDAVNLRSRSV

RSADYFPQFHHLSEESQHRRVKTSVSFREIPTFLHQQNEDLFVVCFDKRDGNNASEDIKNLKS
GSRSARGNMKERVAVKKKEKQQIKKRNVEPKKSYVKG
SHVLPYYTKSNEVYIRSSPRIQKKRRTKAPNSYCEEQVSSGIKHARMRENRRTSSHARIGSSSS
TGSCSESTTTTSEDSRKAGQNFFKQSKAAVTIGNQI
TESEGLVKSKVMNFYIKNLAEVCRLNEEDAESCHDWKWMKPESLKFDDSGDICLEFEQCILDTL
LQQLVDELC

>DCAR_027681 (DACRv2_Chr8:20635645..20639639)
METKEQTPSVIARLMGFYETRHQRPIHKKYRVLSEDYLRKSASIDLLLKKSSCNGRSFRMSRVK
MPEFKDSFEGQEQQRQYIPTILQGR
ETSQAAKESIKFEKQIPKDKRYVELEERILDSWNFGYKLENVHSRKNRSLKYSDKRDNLFPHHS
HNQLCTHSSFGQSTVSKLYDVPSYRNDENSWKPEKT
TLRKNVLRSPQKLEYDSDALEDLHNDLQNISKMRVKLKNRIPVINEASDTGKPLINLEPCKPSFS
TKKTVMETEVSRSALGADDNSVRETKTITPFRSSF
SNLENQEKASYPHSNWSTFTRAGKDIDAGWQKWHVGRKTKVSAVSVSLYSFDQKRAESTYKL
SKTGCSNFSQNSEYSKLSRPFGVNSNRGVKKDVVTKPS
RFRSSPSFPNSTGNFNSNKNDGFGSDEYLRLEEAVTEAGNRFGNQKFSDKGMSKPGDSTFNS
ETSPFSGYTDLQNHQSTEECLLNELGNKLEDGTFSKKI
RKKSAFLEVSHTPVGQESPTESFEDGLVFSNCFEFEPELTSSWRDTHQPSPNSVLNPPYEDEI
LSDIECFDGAEPAGANLIGLWKQLQLLKSDVEDTLSD
GPGMMISSDEGTRDVSDDFLKDNGKSGVFKAKETRDFSYLVDVLDEAGFYDANWEINIEKWYS
PESAVNPAVFEALEKKYGDQISWEKYERRLIFDRINS
GLIEILQPCLDIPEWANSRSTWLGTLPKRDVIEEKLWNLLVSQEEEVNKGLSEKAVGGDTSWLK
VGGDIDIIVREIENLLFDDLVAELGNV

>DCAR_028703 (DCARv2_Chr8:2501854..2504094)
MLSRLQEASQYVARAKKKEKEKSASFSDEVGIGSSSDRFGYQDYQNSRFCNENSSTDCYDEL
RKVIRESLGRQNLLSNGSINAKAYSDR
RETEFSTDLPSSNSSRTSTSYSHDSASVSSNSSNMSEDRPKASNLVAKLMGLEEISSMPVQSE
SRKQVERDKNLNPKGLIFDIDRPIQRKPGIAAQNGDR
GCMTLEEIIQNMQFKGLLDRNQFQIYDSSESEDQLRNHAPPVVLMKPVHAGDGANDFFSRKCI
PGEGDVDSGKRRSMLKTKEGSTSRILEHKIPETNVAK
PTRKAIRRSADNKVKLTNSVSPRLQKDPIDKKVDKVPKSTSRRKPVEKENVKSTGLLKNREQPK
SVSRSHEHLSSPAMKLRKSETGPSIHERKLTTQQNS
TKSKLINKALASNFKNGLKPTITSTNNLQDPSIVHKGKEAHTDQRKKADPNVKDTTATPQLRAEE
EFDDPEVLIKDIYVDSPSHTTKHEYDSSCPKEHDA
SETTFHDTKTTSITILPRSFSFLTRVEDLFDNIDTYQPMALQILTGLHCRDGTNSNLLMDCANELL
ETKSQRTIPCIHPLIQRPSKNPSFCISADHLMQE
VRSGMESLMSYKNHAGEMVSTSAVSALLQRDLWCLGRKVEAWDVGWRNGFTGDEVQTVLW
DLDELILSELVAEVLAEICS

>DCAR_029362 (DCARv2_Chr9:5391980..5397183)
MEAERRSSKGGFFQLFDWNAKSKKKLFSNKSALPECSNQGKENFSSSEASRLHQMNMHEVG
PSPSVQGHDYNCISSVSGEEGHGTKVPG
VVARLMGLESLPKKDVSELSYDSSYYDSYSFRDSHFPRTQPDTQSDHHIMDYGNTRNKLDGF
SRNPVDFRMHKVQNRSIDRFQTEILPPKSARTISSTHS
RLLSPIKSPGFILTKNPASIMEAASRIFEQSPQSTTKGKTSFGSPSVPLRIRDLKERLEAAQKAPQ
VPVQYQRTKDNNSEKCMKGQPNERGQRGLENTQL
RHSVALKQGGSRSLKNKEKTPSSSRTKTNFQMREGPSWSGNRSLNRKGDNGVKPNHLEKKQ
VYMEKGVQRRTSASGTSDVLKQNNQKQNCASSKETICLK
PQTTSCQLDRKVASSKNSSKVNKPVNKIAESSSTGSRKKNIVAADTRKETPSSNSKSFLAKKRP

VSAVARNNSNAIKDVLINKDDRSVKCNVSVDGLKNW
DAVDAKSGMDVVSFTFTSPIKKSLSGSQASGHFREKDSSLSVVPADDKPANVSYLSSLGLNVIG
SDALSILLEEKLKELAYRVEPSDNNLVRHSQFSGPA
SSLHDSASSLSATDSTYAKHGKNPHLDLHKHNSEKQQDFSHSSVDGMILDAKQKWQESEDFE
EISNSRNNKKTEYGQETDSPISSPTSFSGGSCNSLDSK
ISYTSNGNRQCSSLESYETVSFISSRHPHSLEELEFSDSASSLSVGAMERKNRACTSGYRNFEP
SPNWELVYAIEVLSNADFQLDDYVLGQACLTADFFT
SWENQKTGSDKAEDEYYKLEQKLVFDFLNECLEFRCEQISVGNHKAWSKLTMLFLKKDLLAEE
FHREMSRLTSMKDLPTDEIVDKDMSSHYGKWVDFETE
ECEEVLDLGDEILSSLVDELMIDLLS

>DCAR_030482 (DCARv2_Chr9:25854383..25828162)
MGVDKQGSKTGGGYVGGFLHLFDWNTKSRKKLFASKSDPPERLKQKKRDDGNYLTTQLHLM
DQDDSLAGSSFKGSSDYSCASSVTDDDI
GGSKAPGVVAKLMGLDSLPTSNFSEAYSTPFFDSRSLKDSYYHKRSEEFNDNHPIMHSGNMF
NRTQEPLRNNIDSKQQKTVSSPFKKFQTEVLPPKSAKS
IPLTHHKLLSPIKSGGFHPSKNAVHIMEAAARRIESGSQVISKAKMPPVGSSVPLKVRDLKERAE
AARRPLKLAETSQKPAESIAVKNIKGQSTDKNLNR
SLDTKTFIASSDLAESSVGSRNKGKSVSLALQAKVNVQKRGLTPNCNRNSVGLKEQGEVTSNQ
IFKSQPSVQRSSHKKSHTSNPPSVLRQNNQKQNCSTE
REKVASKSLPYNNIQGKKVISGDSSVGRQRSSSKNSGNSKVGSRKIGREIIDDGKDLPYSSTSV
TRKKRCIDGDFNFQKDRAVVDNENNGKATQSDGVMD
SKFSWAEDSKRNGMDVISFTFTAPMGRSLPVPETSRDVLEKNNAFSADFEGKKVFFNSGGTN
GLRSSSVGCNVIEGDALSALLEQKLRELSLRVESSGHK
TGEAGSSASSFQDQTPLKTVAKPTKLHVEGSKRGSWTDSLDEQQSPVFSSTTYEKGRISKHKL
QDVEDMFDCGISSSEARKMLSCRNPSPVSILEPSIFA
ESCNSTDTGDSFSIEGLVSSKQCSSSVQGQDVYDIRCSKKFHVIETDAELSDSASSSSTRVVAT
KHANIAVIDAVRPVKWELEYVKTILCNTETMFKDVS
AGRTSEIIDPRLFDQLETQKGELCLQRKVIFDCVGECMDLRFRKYVGGGYKAWEKGLSMARRE
DWLAEEIHREISSWEAMGDCMVDELVDKDMSSQCGRW
LDFSVEASELGAEIERRILNSLLNEVISDILVL

>DCAR_000349 (DCARv2_C10729808:1394..3970)
MQKRREHVVVTEMVQVGCKWKLKGFFAHRHCRPKKNLLRKNQNQEVTGAEHQRGKSSALG
SNLQGIKSAPKNKGEGIDDEINVPKLIQE
SDDMHKFALEILDSNNELFLKVLKDPNSLLVKRIRNARNLQQAKKETVKLLYENKLVECGNNAISI
KSQKPTHHTNKSLLERIKLQYGCPSTDAEVPSTS
NSIVLLKPRQTKHERDIFRKHRNLESSNRATGGKKLHLPTVSSFNQRDIEAKKHLSEMLRNAEN
VEHYTDKQGPRTLKSILSSPLSSPVHEYSTASNQEK
GMDASVCPQRIQFSSCSNYEFARDDNSSPLQNSEVAASSTLMKLTGDSPIVRFTKEITEPVYSS
DGLSYEAYANDMEMNDTIISEGLDISEAACAEQSST
FFECPIQDFWLENKPLNSSSVVFSSNPWTEREEHPSPNSVLEALFTKDFTGLSSNTNCSAISHT
RPHHTDGKEASLVKDHIRNLLQTTELHWEELSKKSH
PSIQLLNISSLVEHTLFLDFIKDILLELHQCHFDFYPRVPSIRQNTLASLVLAKNMMEEFMEEIQW
YLVPPAMPRTLEDLAIKDTKKDEIWSGRKLETEE
VVTQLTDCMLEELIMQTTYVGEK

Carrot Ovate family proteins (OFPs) amino acid sequences with homology to tomato OFPs.

>DCAR_008765 (DCARv2_Chr3:1037958..1038569)
MPVSFNTQHCPSPSQKIYNSPVAFGSDDSFAWPITPPPKGGNSKPQRINNKNKSKVSSVSSGE
SGWFSSDEDGKESLVSSSENFDTSCD
FGNVSISSKFGTSKKKNNDRNGGNESVVKKSSTSVFKRLMTSCAVNDESFAVVKNSQNPYED
FKSSMMEMIMEKQMYEAKDLEQLLQCFLSLNSRHHHGA
IIQAFSEIWHLLFM

>DCAR_017254 (DCARv2_Chr5:13671894..13672685)
MAKRLKLHISRAITSSFHSCRSKDPSVSNPIPSFFRFSPNLAVPPPQPPPSAVKPDHLSSKSHVE
PQLFQWQNKEEEWQLVYHKSPPTK
KKKIIRRNKKERGESSRSFSSDSSAEFHPIREKPRKKKKKKGCVTTGLCRLSISSSPARLSVFK
KLIPTVVEGKVRDSFAVVKKSEDPYEDFKRSMMDM
IWEKQMFEEGDLEQLLQCFLSLNSPRHHKVIVDAFAEIWNAMFADSNITGTSTTRNSNSVSVVD
CEPTSSIDFS

>DCAR_013429 (DCARv2_Chr4:30253465..30253995)
MSHRKKLTVRNVTVNLGCGSCSRTSRLLSNIFNPKPKPIHKPLPSFRQAHNPSYSSTSSWQTA
DTSTYYSDSTDSDIRSLRAVQGFGKI
GGESLAVEKDSEDPYLDFRQSMLQMILENEIYSKNDLKELLNCFLQLNSPYHHGIIVRAFTEIWN
GVYTGRAGSSTNMHGVYKSRDY

>DCAR_020721 (DCARv2_Chr6:28906664..28907131)
MSSHNKKRRVILSNTTVKLGCYSCRKSSKKFLFFFSKTIHFNLQKLKINPTSRTSSSASSHFHTP
VLSFSPAPSRPLQGFGRIGSDSLA
VEKDSDDPYLDFRRSMLQMILEKEIYSKDDLKELLGCFLHLNSPYHHEIIVRAFTDIWNGLYSSY
R

>DCAR_012074 (DCARv2_Chr3:43525622..43526404)
MENKFKLKISRMFRSTLDSCRTTSLSDDVASSEQPFFFPENTHHNHHLVELFSPKPPPPRSFPS
LCRPKSHENQTTLFNDFNSKPKHKK
QRSKSRKNRKRNNRKFDEFFCSVAANYYGLYSSDEDEKENVMDDYETTFFSSKSMSSGSLKS
SAAAYNNVVALEKMREEEYFNDDDDDVELMKISSPLNG
KVKDSVAVIKSSSDPYNDFRTSMVEMIVEKQIFGPKDLEKLLQCFLSLNSGAHHRVIVEVFTEILE
TLFSD

>DCAR_010451 (DCARv2_Chr3:23239734..23240519)
MENKVKQKFSRMFRSSLDSCRSKSRSDVSEQPFFFPENRFHHHQLIQLFSPKPLPARPLPPFR
APKSPQTTGPTSPLPPPTTTTTNTLF
KHCKPRHKHRRRRSQSRKTRQKNNDKFDQFFTSVAANYYGCYSSDDEKEEENASKYDDDTT
FFSSKSMSSDSSLAMEKSSTKQQWDNFDDDDDDELTAKI
HADSFAVIKRSSDPYSDFRTSMVEMIVEKQIFGSKDLENLLHCFLSLNSLIHHRVIIEVFTEIWETL
FSDWD

>DCAR_016895 (DCARv2_Chr5:8913728..8914282)
MTNIFRCSPRKKSLISDVTEHPFFYPHSFHLFSPKKPPPSQPFSSICRPKTTQTAPVKPRHRKSR
KRRNNSELKNKKFDELLDSVSGNY
TNCWFSEDENEGKDDDRTTLFSGFKDSYAVVKRSSDPYNEFRTSMVEMIVERKLFKARELEHL
LHCFLALNSSSHHMVIVEAFTQILETIFSGYQ

>DCAR_025926 (DCARv2_Chr7:31757169..31758107)
MAQRFRLRITRVFRPKHSPPTPTSAAPQPSKRRRIVSFKRRLSSALLCRCSSNFNNSSDDENTA
KPLDFHWQNDQKEWHVALQNDDESP
RQKIYNSSVSGGDSDDTDILAPTLLPPPPHKKRRRRKKRAPLLKHNRISTSSAETETLVSSSRSF
SPEETETLVSSSSRSFSTDELETIREKPPKRHRYR
KPKNKNLEPMTSSSRCSVTVSESESPARLSVFRKLIPCAVEGKVKESFAVVKKSVDPYEDFKKS
MSDMIFEKEMFEEKELEQLLQCFLSLNSRQHHGVIV
QAFAEIWNSMFSLNTATNSTISN

>DCAR_018654 (DCARv2_Chr5:31914846..31915919)
MIPNAWFYKLKYSTRTTKSPNSMQKLKKNNHAKTKSPAYYSSLSSSSGGAAAASQRKSYHITR
DLPPTPDKNSASQHSDSPRRSSRKRG
SNRKKRRSIRSTSPRLLLSAPVSTDCSCRASHEDCQNDPLVESSYSEDDYSVFPELWSEKEDK
PFHKMVSSCHCRAKSDIPIGMTMSKDSKCENFDPILQ
FELKPIITKINKVKKNDEEVSKIRMISATNDESISHSFYKEQKSVNSVKKLSFNTNSTGVKLKTDS
PRTASKRFLQGHHRKSASLGGARRSLAESFAVVK
TTQDPHKDFKDSMMEMITENNIRTTKDLEDLLACYLSLNSDQYHGLIIKAFKQVWFEIADMKHIT
RDS

>DCAR_015345 (DCARv2_Chr4:13256186..13257259)
MGNYKFKFSDMIPNAWFHKLKHISRPKNDSTMHKSKKMSLTRTKSPAFSSTLSPPSGGAAAAS
PQPHHQRKSYHFTRDLPPTPDNNSAS
QLLDQPRKSTRRRRSIKKNRPSIRLASSPRLLMNASVSTDCSCRASLDEHHNNNPTDSCSSEE
TDLSVFPSENGDKPFDIMVSSCHCRVKSDIPIDMTMT
KDSKLETFDSVSQFELRPIITKTNKIDTTDEEVSKMRISHSSLKEKRNLNSAKRVNTTSSPGVKL
RTNSPRLASKRFIQVHHRKSVSSSGVARRSLAESF
AVVKTSQDPHRDFKDSMVEMIMEYNIRSSKDLEDLLACYLSLNSDKYHELIIKVFKQIWFEITDIR
FK

>DCAR_007928 (DCARv2_Chr2:38299219..38300714)
MKWGRKKPSSSTNPRVISHVFAKSWFSRFRKMSEDTKPVVGSVKGEEKKSLSSPRTQGRFHE
GHDCSYLRLSLGAERCKCQHLKSVWYD
SDDEIQVMLQSPPCSRHRIRELEGQEVVGRRAVSELPRNVEFLQRNYICDREKEDLKSKILKRR
AMKDPKSRTMHQTAHEEKQSESPRKLYTSVENFTKP
VHKGNSELEPARNIQTEKDHQKLNALQLRKKRGNYSTINSSLKGIQERCALGTRKMEQDDAAC
EELLFTEWQNMKDAKINELMLKSEEQKRNVLLRRDSS
RKRTKQARRVRVYTPRTAARIECKIRALEDLKKAKIKTTRARKMRPAKEATAFDSFAVVKSSLDP
HMDFKISMAEMITAKCIRQPQELEELLACYLTLNC
DEYHDLIIEVFRQVWFELNQVEIILPYNSIPSKAENSEKFHT

>DCAR_006331 (DCARv2_Chr2:24347085..24347852)
MGKKIVKFPSMFKKRSSSDQWQWPSSGKHNVNYENGDELMFKTVNSVFLEPCIGDQTSGDK
PSYGWFTNAEYSPSLSDTSEQVEDEDDQ
EMEDELEEIIRGVRSDRLFFEPCNSLLGSSTTTTTTTTGTAATSDTRGASDDVCYNDVEELPFK
ESIVLAMESEDPYEDFKGSMLEMVESHGLKDWECLE

ELLEWYLKMNGKMNHGVIVQAFVDLLVGLASDDSTSFSSAASSFSASSSSPLSTVSRRELCNH
DHM

>DCAR_012488 (DCARv2_Chr3:47514527..47515120)
MPRTLGRNLQMCFPKIIYPPPPQKSIKKLNPQEKTNPNPSTLSSILIKNFNSLYQSEDSDTTPPDF
AAVYASQRFFFSSPGHSNSIIDS
ATSSLSLSPSSSSSSTNTCSSTRADNVVVDGCVATPTLSPDPYVDFRRSMQEMVEARELGDV
RANWEDLHELLTCYLSLNPRSMHKFIVGAFADLLVSLL
NPAGPGRD

>DCAR_018427 (DCARv2_Chr5:29731440..29732081)
MPRTFGRNLQMCFTNIMHPPSSSQSSSPIQQQTQENNNQNPSKISSILINNFNSLYHDPDNYNH
ATTSSSSTMPPPSPQSDDTDSTTPD
FAAVYKSRRFFFSSPGHSNSITESATSSLSSSSCSSSLPERDNGVVDGGVPVPTFSPDPYLDF
RRSMQEMVEARDLADAREQMDYLHDLLTCYLSLNPKS
THKFIIGAFADLLVSLMTSSPVEG

>DCAR_002726 (DCARv2_Chr1:32323256..32323738)
MINPQTSPPHEDDRSHFLMKNFNSLYDPTTSHEPSSSPPDFATAYASQRFFFSSPGRSNSIVD
STSSLASTSSSSLLPESDTLSGGIPV
PTLSPDPYLDFRRSMQDMVEARGFTDVKTNWDNLHELLRCYLSLNPKSTHKYIVRAFADLLISL
MTTSPQP

>DCAR_030639 (DCARv2_Chr9:28494805..28495419)
MFPFTWKNYLLCFSNIKTLPITALPNTSPSNQIKNFNSLYSLNSTSTSNLTSSADHDFFSNASDV
SSEGDDSPPDFATVFASQRFFFSS
PGRSNSIIDSPNSLGDPECSNLVPGSVAIQTYSPDPLQDFKKSMQEMVEAHGIIDVKADWEFLH
ELLLCYLTLNPKHTHKFIIKAFSDLLVSIMSSESCP
KIEDCQHGTSSPPLA

>DCAR_013428 (DCARv2_Chr4:30267772..30268701)
MGRKMKLIPFLFKPTEQILSSSSSASSWPWPNCGSPKTLSFRAEKIMNSVFVANDSRFLTSSSV
CEQSSSILSTTVVLEEEEYLAEGAD
QTIESVIRGAKTSSERLFFDNHAGETTSCIVSDQHQLTKSNDNKSGDVTSSATNRIDCATSNER
YKYCSSDKVLLMEMDSRDPYDDFKKSMEEMVEANGF
NVKDDWESLHQLLKWYLQVNGKSNHGYIVGAFVDLMVSLEYSSASSSFSSPTSSSSSSTDYD
HNNSSSSSATVITQSPVSALSFSSNYSTAPCLLTLFEE
EEEEEEEEEKDAALDHRGST

>DCAR_002384 (DCARv2_Chr1:28822039..28822674)
MFPFTWKTYLQCFSNIKCLPITTLPDPNQHQNLHTTSTTLMKNFNPLCDLTFTSTSNLTSSLDHD
SFSNASDISSDAEESPPDFATVFA
SQRFFFSSPGRSNSIFESPSSPQAPRKNFLVPDSVAIQTYSPDPFQDFRNSMQEMVEAHGIIDV
EAEWEFLHELLLCYLTLNPKQTHKFIIKAFSDLLVS
LMSSQTCRIISCQRDIASSRLV

>DCAR_020722 (DCARv2_Chr6:28889122..28889910)
MSKKMKLPSPFKYTKSPSFPSSTWPWPTCADPKAFSFRAENTTINSVFVPEEHSEFIASSSDSN
SASESSRRHVISTADELDTECFNSA
DAIESVIRGVKKSERLFFEPSSSTLREPEKNKINNDTTNEDENDSNRASKYVVIEMETMDPFME
FKESMKEMVEAHGLDSLDGLEEMLSCYLRVNGKCNH

GYIIGAFVDLLVNHDEFDFTFSSTCFSSDYQSTTCISQSPVSASSLSSFSASNCKCTTAASGASS
SEDEEARA

>DCAR_010565 (DCARv2_Chr3:25523639..25523953)
MTMDRPEGQNQHHRKHVRRNQISFSACLPTDVRGIYADSICAVKYSSDPFSDIRESVLEMIETV
GVRNWNELEELVYCYIALNPTEVHH
LIEQAFVSVFISYAT

>DCAR_016924 (DCARv2_Chr5:9159966..9160859)
MGKKMKLIPFLHKPTEPAPSSSWPWPSCGIPKTLSFRDEPMHNSVHVATESCFFNSSARDLSA
DTIEQQEDEDLDKTIENVIRQARLSS
ERLFFDNHIAEATSPQLKLSKTDKNSTDFNKNNPVTSTSSYISSSDAKLVVMGMDSTNPFEDFK
KSMQEVVEASGLNMKGDRDSLDQLLSWYLKFNCKSN
HGYIVGAFIDLLVSLEISSASNTTFSSPTTLLSSGTSSCDNEGKSSSDLSSATVATEPPVSVLSFS
SNWSNDPCLLTLFEEEDDEEVAAGLDHHLIRDQE
ANKISETS

>DCAR_016221 (DCARv2_Chr5:1326740..1327597)
MPRQFHKTIQDYISKIKKPTSHLKLPSKSLTSSTSWILRGCKHPKTPSFTIDPEKQKYHFSDTED
QDHDDGSATLSDIDKFLIENFRSL
YAKELDQGEEHDQENEGRVSCDSPKYDNPPSHDLCSSHRFFVAPASSGSVIDDPRTSLAVSE
GIEGPGPGNTIFDGSTSECSEKKKMGAEDFIAVLRYSS
SPCVDFKSSMQEMIEARLHHNGKVDWEFLEELLLCYLNLNDNKSHKYILSAFVDLIVLLRENSVK
VEDNSGPSGEVPARFPDVKKPQDDKRKGREV

Carrot IQ-domain (IQDs) amino acid sequences with homology to tomato IQDs.

>DCAR_014966 (DCARv2_Chr4:16758215..16761372)
MGRKGNWFSSVKKAFSPDSKDKKNKRANKSKNSVEKEKSSSPDFSTLETISTSQHLPPPPVVQ
TTEIVDEQSKYAYSVEDANTSSYVPAVEAPEVVTEVVRPTPVTRFTGMSREEVAAIRIQTTFRG
YLARRALRALRGLVRLKSLVEGATVKRQTTNTLRSMQGISRVQSQINNRRIRMTEENQALQR
QLLQERAKELESLQMGEDWNDSAQSKEQIEAKLLSKYEATMRRERAMAYSFSHQQTWKKSA
RTTNLLFIDPTNPQWGWSWLERWMAARPESKTEKEVTNG
HSSVKGASLNFAGNEIAKSFARHQLNSDKPIAPVQRKLSSPSNLQSPSIPSSKVTSPKPARKFK
PPSPGASVMSIDYDSKSMMSVQSEQNRRHSIAGSSV
RDDESLASSPSVPGYMAATRSAKAKSRMQSPLGMEDGTPEKVPAGYAKKRLSFPASPARPRR
HSGPPRLDSTSIADNNVNDIPVN

>DCAR_020875 (DCARv2_Chr6:27643046..27646563)
MGRKGNLFSSIKKAFSPESKEKKKQKAIESKNKGIEKEKPLNPESSTLETVSMPHSLPPPTEVNF
TEIEIEDEQTKHANFGTASTSTLA
EDAAEVPLGVTEAVQAVAVTQIKSKMTDDVAATKIQTAFRGYLAKRALRALRGLVRLKSLVEGS
AVRRQTTNTLRCMQGISRVQSQINSRRIRMSEENQA
LQRQLLQKRAKELESLQMGENWNDSVQSKEQIEAKLLSKYDATMRRERAMAYSFSHQQTWK
KSARSTNLLYMDPTNPQWGWSWLERWTAAHSGESRTVKE
HNNDYSSAKSASLSFSGGQIAKSYARHLLNSDKPNSPNNPRPRDPSSIQSPISPLSKATSSKVG
KKYKPPGPRGSVMSPDFDSKSLFSMHSEQNRRHSIA
GSSVRDDESMVSSPAVPSYMASTQSAKAKSRLQSPLGMEYGTPPKGSTVYAKKRLSFPASPA
RPRRHSGPPRVDSTSVADLNDVAVN
>DCAR_006462 (DCARv2_Chr2:25712341..25714895)
MGKKGSWLSAVKKALSPEPKEKKDKKQHKSKKKWFGKNKSIDAGFPQPTETAAAIPDPSPSAE
EVKLTEAENEQSKHAYSVALATAVAA
EAAVASAQAAAEVVRLTSTARYSGKSNEETAAIKIQTAFRGHLARRALRALRGLVRLKTLIQGQS
VKRQATTTLRCMQTLARVQSEIRTRRLRMSEENQA
LQRQLLQKHEKEVDKLRSPRMLRNSSKSSNPTFMDPNNPHWGWSWLERWMASRPWESRSA
IDRELNSDRASVNSATSHAISVGARRDLNFGNNLLSPAGK
KLSRPPSRQSPSTPPSKAPSSSSVGGKMRPPSPRGSNCGTEDDSRSFRSVQSERWRRHSIA
GGSSAGDDESLASSPAVPSYMEPTQSAKARYRLPSPLGM
EKNRTPDKQSPAATAKKRLSFSSSTVGPRRHSGPPRMDSSSVKDIAMDL

>DCAR_017740 (DCARv2_Chr5:23020922..23023972)
MNRASGISNTTADALQRITCNAREEITVLYYDEESNQSCSYSDRTHCTRSERSSDFTCRYKSVI
VATMGRKGIACFSSVKKAFSTPKSK
EKKNQKWKKWFRKQTSLKRKSSLEACSTPQNRAQLPQIHTTGIVRPITVSRVAGKSREEVAAIK
IQTAYRGYTARRTLRALRGLVRLKSVVNSAAVKRQT
TNTLQTMQGVSRVQYQINSRRIRMSEENQVLQKQLLRKRAKQLESLQMEGEWNRSSQSKEQI
EAKLLSKYEATMRRERAMAYSFSHQQTRKKSARSTNQL
FMDPANPQWGWSWLDRWVAARPESKTEKEFTDDHSSVKGGRRNFAGNAMAKSHARPQLIC
EKPMPACYSRLYSPRNHLSPSTPVCKVTSPKPTRRLKLHS
PGASVLSIDYDSKSMFSIDSELNRRQTIAGSSVRDDESLVSSPSIPRYMAATHSAKAKSRTQSLL
SMENGTLAKKSAGYGKKQLSYPASPARATQHFSHP
SLDSPSIADNNVSDIAVN

>DCAR_017741 (DCARv2_Chr5:23024880..23027930)
MNRASGISNTTADALQRITCNAREEITVLYYDEESNQSCSYSDRTHCTRSERSSDFTCRYKSVI
VATMGRKGIACFSSVKKAFSTPKSK
EKKNQKWKKWFRKQTSLKRKSSLEACSTPQNRAQLPQIHTTGIVRPITVSRVAGKSREEVAAIK
IQTAYRGYTARRTLRALRGLVRLKSVVNSAAVKRQT
TNTLQTMQGVSRVQYQINSRRIRMSEENQVLQKQLLRKRAKQLESLQMEGEWNRSSQSKEQI
EAKLLSKYEATMRRERAMAYSFSHQQTRKKSARSTNQL
FMDPANPQWGWSWLDRWVAARPESKTEKEFTDDHSSVKGGRRNFAGNAMAKSHARPQLIC
EKPMPACYSRLYSPRNHLSPSTPVCKVTSPKPTRRLKLHS
PGASVLSIDYDSKSMFSIDSELNRRQTIAGSSVRDDESLVSSPSIPRYMAATHSAKAKSRTQSLL
SMENGTLAKKSAGYGKKQLSYPASPARATQHFSHP
SLDSPSIADNNVSDIAVN

>DCAR_018751 (DCARv2_Chr5:32812332..32817601)
MLQFVTHTLLEKAPSSGKFLHRRRHSVKIENDVLQDEFNLKTVPPVKAEHSVSDAGAFSSNVL
QMVDASQVEPNMREERAATCIQTAFR
GLLARRALKALKGLVRLQALVRGHAVRKQAAITLRCMQALVRVQARVRARSVRLALENQTSEQ
KLKQRLEDEARVRQIEEGWCDSAGSVEQIQAKLVKRQ
EAAAKRERAMAYALAHQWQAGSRQLAPSSGYEPDKSNWGWKWLERWMAVRPWENRFLDIN
QRDGVAIQDNEAGDGRNDSLKKFSAKKTIPLRTESNTTSE
KIDLSNSVGCNSTATRSSDMQEASGTSSTKVKTKHIFEDLVEEVNSRPGIGSRSQSNPKERSSL
SDKQGRRRQSLPNIRLKNNN

>DCAR_027520 (DCARv2_Chr8:22296761..22299927)
MGRKGSWFSAIKRVFVPNSKEKLEKGSEKRSSKEKKGRGILKHAETKSFIPLFREPSSIEKILGE
VDQQQLQFRPPTPYELPKPPPSLP
ARPASPRSASLRVTSPRNASQRITSPRAATSRRVTSPRAASPSVTSPRAATPRAASPRGYRPR
PEPTLRNHHLSATKIQAAYRGYMARRSFRALRGLVRL
QGVVRGQNVKRQTSNAMKHMQLLVRVQTQIQSRRIQMLENQAIQRQANKNERETESTLGKW
NSEAGDNENWDDSLLTKEEVEARMQKKVEAIVKRERAMA
YAYSHQLWKGNSKPGQSPPGDIVSGGFPWWWTWLERQAPSSKPPENQTMKTFLQAPMRMT
PEHKPSPRSHTSNYKPQNFGYDNLETPTPKSSRSAAPVRT
RQMFTPTRTPPSTSSNMTKYSRSRNFAANSPYDVPLKDDDSLVSCPPFSVPNYMSPTVSAIAK
VRPTSNLKDSAPGTPGSQTSKRRFSFPLAPSMGSFKW
NKGSSKDSASQKVMEKPRAPPSIGDRSVDSTVSMPAAFGRKPFNRFV

>DCAR_015494(DCARv2_Chr4:11468234..11474220)
MTGVRLAVLLKLALWAFLENGLEHWLVQRNHRGHNLQKKMKMLRDPDSGKFRHRRRHSIEND
VLQDEFNDKTATAVKTENSASDAGGSP
SSSLQMINASQFEENMREQWAATCIQTAFRGVLARRALRALKGLVRLQALVRGHAVRKQAAIT
LRCMQALVRVQARVRARRVRLALENQTSEQKRQQQLE
QEARVREIEEGWCDGAGSVEQIQAKLLKRQEAAAKRERAMAYALAHQWQAGSRQVASSGFE
PDKSNWGWNWLERWMAVRPWENRFLDISQQDGTAVDTEV
GEGKNGMMKKLPAKKAIPLIPDTNTASEKMVLSYSDGCDSSPTRSSSMQEASASSFTKGKTKP
VIDDLVKEVSSRPGVGPRSQSNPKERSSLLNKQAKTR
QSLPNVGQGEQGVGVHTSKQLARTAVRRKPTAQKPINDKTKINGQ

>DCAR_020324 (DCARv2_Chr6:32327250..32331581)
MGKKGSGWFSSVRRVFKPSSKDLPPDSRKENGNKSQQDVPEVVSFEHFPAESSPDTTNDDS
NEDSFSPVTEDQHANAVAVATAAAAEAA
VAAAQAAAKVVRLAGYGRNSKEERATIFIQSCYRGYLARRALRALKGLVRLQALVRGHNVRKQ

AHMTMRCMQALLQVQSRVRARRLQLAHEKFEERIKEE
YKLAPKKDNHTQKTPERSFGSKACDYEDQTREKIKGSSMRKHDADLKRERALAYAFAYQQQKI
AHSHWDLHGDEFAQLSNEHDKQQWGWNWLERWMASQP
GHLGNLGPHDSSYTTLTTTDNMSEKTVEIEMAMPMASEHVPMGRLRGNTLELAQFSTRPNRQ
SGLDVVPSYMAPTESAKAKVRSQGPMTTKPRNSSANQW
NTSTGRGPIFGLGNADSSSSGGGTAGYQVLKSPKSSLLQHGQAKWMTGYSPDSSGGDERFS
HGWRHSMYSKMIFYACIAKGTTILAEFISKDEDLGALAL
KCLEKTPQFHSMYSHSVRKRIYMYLIEDPFVYFGIFDGNLEKPLGLDKNSVKKLSNLKPHCFQG
DLYPVFHQLVAAPLGEESLDGGKLVVSDDSGSCSVR
DNRVGLTPLLGGSEKNVMKKKRRFLGEASGCKEEVSKENKVDMSCDNGITSSREFSLISSHKN
GLFVVDKAKRVWKKQLWIVLSVDLIVCCIMFAIWLWI
CRGFKCISE

>DCAR_005240(DCARv2_Chr2:10321682..10324413)
MSTNVSHHVCSGNPGIRIEDLAATRIQSAFRAYRLKGTVNLRAVTLGSSSKKQASVTLRYLHTW
SRLQTEIRVRRASMAVEARIKQRKL
ENQVKLDTKLHDLEVEWSNGSETMEEVLARINLREEAAVKRERAMAYAFSHQWRASSNPALG
NYELSKAIWGWSWMERWVAARPWESRALTQSSPKKVVS
SKKVSNTAKNMKSPTIKSSNSVKSMSPSGKITTNPRKLSYGEADQEANTKKEQIVSS

>DCAR_004026 (DCARv2_Chr1:44070118..44075238)
MGSGGWLKKMIGSKKEKNGNAKSLEVYSQKDSSNTVNNIADEEPVTVAKSIEDIAATRIQCSFR
AYKARKILRFLKGTLKLRTLTLDSS
VSKQASATLRHLHTWSRIQSEIRARRISMATEARIRQKNLENQLKLDAKLHNLEVEWDCGTKTM
EESVSNVYLREAAAVKRERTMAYAFSHQWRANKNPV
LNNNELGNAIWGWSWTERWIAARPWESRALVQSSPKKSMSRQSSKTSKNLKSPTMKPMRSV
KSISPNGKGTIKSRKLSYGEPGQQAAANKEQMAWEIALP
GGKTEQGDADDVQTALREAHEEIGLDPELVHVVAVLDPSTTRYGVTVVPVIGILYDKRKFNPVA
NDAEVEAIFYAPLEMFLKDENRSETQTEYIGEKYLL
HFFDFETDNTKYQIWGLTAGILITVATVVYQRPPAFTELRPRFWSGNHQCKF

>DCAR_008942(DCARv2_Chr3:2869659..2871818)
MDTNYCNMKDSANKWQQDVPEVVSFEHFPTASSPDATNDESNMDSSSSPARRALRALKGLV
RLQALVRGHNVRKQAQMTMRCMQALVRV
QSRVRARRLQLAHQKFEKRIKEEENLAAEEEYYRLRTPMRTFGPEAWDNRNLIKEKIKENSMR
KHDAEMKRERALAYAFAYQQQQQNEYLHSDLKGDEFA
QRSSEHEKQQWGWNWLERWMASQPHHMRNGPHDSSYMTLKTTDSMSEKTVEMDMAIPLG
SEFVPMGRRLSGDTLEPARYSTRHNQHSGSDSVPSYMAPTK
SAKAKVRSQGPVSAKPRNSSVSSWNPSTKRGPAGTGDSSSSGGGTVSYQVQMTPSPKSNAL
HQGQPKWMAGYSPDSSGGDERGHLWRHHYT

>DCAR_007973 (DCARv2_Chr2:38613511..38615084)
MGAGGWLTKIISSKKEKNSKEKFIKVHSAPKSIDGMNEDISSQEEITYNDKNLVPLAITVEDIAAIR
IQNAFRAYKARKVLRHLKGTAR
LRTLTLGSSASKQASITLGHLHTWSRIQSEIRARRVSMATEARIRQKKMESQLKLDSKLHGLEVE
WSGGTETMEESLGKIHLREAASVKRERTMAYAFSH
QWRATSNSDSNNSEIGKAIWGWSLTERWIAARPWESRALVQPSPKKALNRQSSKNGKIQKSP
TMKLMPSVKSISPNGKGTIKGRKLSYGATDQEATSNKE
QTAS

>DCAR_016145 (DCARv2_Chr5:644696..646236)
MGASGKWIASLIATKKSHRPNDPERMGGGKGKKWTLWRSSSSGSGGGVTTLSEGTKVRGRL
SEAAASDSPFMAAAMATVVQAPARDFMV
VRRQWAAIRIQTVFRAFLARRALRALKAIVRLQAIVRGRQVRKQAAVTLRCMEALVRVQARVKA
RCASAEVQKDEPDSIKQGGWCDSHGTIEEVKTKQHM
KQAGAMKRERAIAYALSQQQLRTNPGLNSRKNKMGACNKFDKNSGMSWLDGWMANKPWEG
RSMEESQMDSPDLSFASKNKEDYSVTSWSNYSEHDSVNVR
RNNISTRISSRPQCSEFLSNESLSSISSTTSDTPGSSCTLAEGSTVKRAGYMNLTESIKAKQRGS
VDSSQLCKKPSPLSKGLARRNAACQFRSVDLS

>DCAR_002978 (DCARv2_Chr1:34558546..34561082)
MGKKGSSSGSSSWLTAVKRAFRSPSSKDSPEKRSTRSRKDEQPSPQYPQPSDDDKKREKRR
WLFRKPSNVIDNNALVPQTPANGLVEDR
HHAIAVAVATAAAAEAAVASAHAAMEVARLARPSTVPQHYNNYDYAAILIQTAFRGYLARRALR
ALKGLVKLQALVRGHNVRKQAKMTLQCMQALVRVQA
RVLDQRMRQSNDGSRKSAFSDTASLWESRYLQDISDRKSMSRDGSSIADDWDDRPHTVEEV
KAMLQNRKDAALKRERTLSQAFSQQMRRSGRNSSIGSEA
DIGETNQRRDRLTNGKQWDSRGRSSTDQRDQIKTVEIDTSQPYSYLAPNYSRANQNQYYLQQ
QRPSSPLHRAQINLQHQSPVTPSPSRTRPPLQVRSASP
RFVREERSYQASQTPSLRSNYYYNGGLQPTRGSTSGAGGVPIPNYMAATESAKARIRSQSAP
RQRPSTPERERASSVAKKRLSYPVPDPYNIGARYSGGY
GHSLKSPSFKSISGVNFGMEQQSNYSSCCTESLGGEISPSSTTDLRRWLR

>DCAR_000804 (DCARv2_Chr1:3015685..3018402)
MGASGKWVKSLIGLKKPDKDDQARRALRALKGVVRIQALVRGRQVRKQAAVTLRCMQALVRV
QARVRARRVRMSLEGQAVQDMLNERRS
NAELLREAEEGWCDSRGTLQEVKAKIQMRQEGAFKRERALAYSLAQKQWKAFQNADSRTNIS
MASHKNEFNKNSWGWSWLERWMAAKPWENRLMEKVQSD
SSEMTPPPKAYADNIKSDLAKSSEQYSVKIRRNNVTTRISAKPPLIGQTTRSSSSPSSEFRYDKS
SASSSFCTSTTPLSANTNMASDRTDDNNNVSKPSY
MNLTEAAKAKQRNTSHRSMSRAEDEFQFLRKSAVFSDEDSKGSAGPEPSLVNMSRLLCKPAS
RVDRSSTKLKG

>DCAR_003234 (DCARv2_Chr1:37178475..37180590)
MPSDGSMAKKSWFSLVKKFFVSDTYTNQYKKQRRRRWFLGKFKIKRLPSISAASPSRETKVSE
AEVAQSKQDCNVASFTDATPDETAAD
VFDQPSSTPQYTYHSEDEAREFSSINFQYNAPPSTPQWGREIQELAATKIQTAFRGCLARKALC
ALKGIVRLQAIIRGELVRRQAITTLNQLQSVVNIQS
QVCAKRSQMADSTSYSQGNKEPVEFKGKDIKIDQNSQRRWDDSLLTKEEENALSSSKRMAAIK
RERIKEYTFSNRRSAELEQNKIDGRWRYWLEQWVDTR
LAKTEDLQNLVSVFSMRDRNKDNEAGVKQVRSRNVHKQSHNEESELPTRNSIHHRKQRSTGD
GYSTGGGSPVVPTYMAATESAKAKSRSLSSPRLRPMSF
DSNSETFSPYKHKLSPISSINSEVTAISMFTNPTSGFMQRSPGFKGPVKSHRSSKYLSVD

>DCAR_031820 (DCARv2_Chr9:11638146..11639957)
MAKKSWFSVLRKFFTVEARSNRDKGQKRRKWISGRLFINPHVVISAPRLSGERTVISETELVHS
KHEYNGDILAANPVPVLVQSPQRSK
EELQEFDAIKAECNTPPPTPQCEMEIQESAAIKIQTAFRGHLARKALRALKGLVKLQAIVRGRAV
RRQAIVTLNRLQSIVNIQSQFRMNRSQTMDKTQCF

QQNKELQEFADKDIKMDFNSQKRRDSGLLTKEEENILCSTRRMAAIKRERLKEYTFSNRRSAEL
ELNKVDGRWRYWLQQWIDTRMANTEDLHDVASVSSP
KTKGREESRSRLTKSRTTRQQCNKDNELDITPRVRPFRHLKHQSIQEDGNFCRGKSAVVPTYM
AATESAKAKVRSTSSPRPRPVNFDAQSETLSPYKHKI
SPISSINSEVTAISMLANSCTGFVQKTTGSKGHVRSKRTLKYLSTD

>DCAR_022359 (DCARv2_Chr6:13773295..13775245)
MCFLGRFRIKRSPSIPAASPSRDTGEVVQDSNVQIFMASAADEIAADIFDQPSCSTSKYTCQRE
DESRDFSSIYCKYNAPPLVPNWRRL
IQEVAATKIQNAFRGYLARKVARALKGIVRLQATIRGILVRRQARKTLNKLQSIANIQSQVRAKRS
QMLDDPSCSKDKDIKIDLKSQRRWDDSLLTKEEE
NDLCSSKRMAAIKRERIKQFSFRNRKQCHYKESELPATRLVHLQKQHPIGNRYSAGGGSLAVP
TYMAATVSAKAKVRSMSSPRLRPMSFNSNSVTFSPYK
HKSSPISSINSDLTTISMFNRPSHRYSQRSPCLEGPVSKRNSK

>DCAR_019733 (DCARv2_Chr5:41372863..41374986)
MGITGELVRSVFSKSKSIRGHEANVRSNQERKRWKSSVRSYLCGDEFNSVLAEEDSASLRSSK
VTVSTEPEFSSTNVDDPASSVWSSEA
TVTQPVPENLVEKTDAESKQNDVDIQEEKHNSTSNLFRKDDAAIVIQSALRRFLARRHKEGLSL
MDCKEKLVVGAESKRSNSVGTSIEVQTGNLTVQSFQ
EESESQLQRVQHKGRPQVLKLKEDWDDSTVSSNISKLRMQNRLEATTRRERALAYAFSQQLR
VCSKKKHTRSESEVVESNMGWNWLERWMATRQQDNCLT
EITKQYEPLNRNQKTATRKRLLFDIGGEEESCGSNEVAIQLDNFSVPAFSKEKEDYTKPLQDRL
KPTSVSRCKTLPRDHYSKETVKLDAQTSNPKDNVKV
DARTWVIKKQSAGEDEIDKKHNKPKQPSGLKKEAECKDATSKASPEL

>DCAR_028026 (DCARv2_Chr8:16166116..16167767)
MAVEARIRQRKLENQLKLETKLHDLEVEWSNGPQTMEEVLARIHLREEAAVKRERAMAYAFSH
QWRATSNPALGSYELGKAIWGWSWME
RWVAARPWESRALVQSSPKKPSPKKALNKQASKTAKILKSPTMKPINSVKSISPNGTVTTKPRK
LSYGAADQQANTAKVSTTS

>DCAR_011147 (DCARv2_Chr3:33005989..33011206)
MGKSPGKWIKTVLFGKKSSKSNAAKDVTSGRKISSVARAPSSDLVENPSAVLETAMQTTNPNG
GLPERATEVTRAPTSEVTLLSGVQGA
DDQGTMPLSSADTDELNRQHQAATKAQAAFRGYLARRAFWALKGIIRLQALVRGHLVRRQAV
ATLQCMHAIVKFQALVRGRAVRLSDVGREVQYKFGEPV
GKPLDMLRANTFPGTLKESSNPFVRKLLSSSPTAMPLSLQYDLGEPNSAWKWLERWSSSSFW
VPLARPNKIIDAKPKRKQAGMQSDEVEAVRPKRSVRKI
SGMNGDSSKLHSSEYEKPKRNPRKSLGHQPESVQEPQNELEKVKRNLRKISATALVPTDTPET
VIEKPINPKKTHGSPKHDASEQVADLSSEKPSDIKVS
DSSVALPTQPVAEAPSESLALDKPVDVVCDNLSAVELQPLENNEKVENSLKVDEDQTYKEDQS
SKESQKRRRRKSNPVKAEYPESVSQNSPTLPSYMQAT
ESAKAKLRAQEAQRFGDEEAENGFTRRHSLPASTNVKLSTPSPRMQKPLQANGKTASKSNRS
LTSSRDGYVAIPNCYEVVSL

>DCAR_030682 (DCARv2_Chr9:28843959..28845597)
MGKKSKWITKFLTGKKDKEKSSNNEHISTSYENPSTPISILPVTPKEKRRWSFRRSSASQTPQK
DLSSVETAAVVVPIQSSLEADNEQK
KHAMALAMATTAAADAAVAAAQAAAAMMRLAAATPGRGSPVEEAAATKIQSVFRSYLARKALC

ALKGLVKLQALVRGHLVRKQATATLRCMQALVTVQAR
ARAQRIRMVDDANPYNQRPSNYRKSTQQDERSRHSHYDLMEENIKIVEMDFGDARASLKSRG
GYSNYTNTDHRFSPNHQFLKQDSQQVSPAPSAINDMSP
RTYSGHFEDHSFGTTQSSPQYYSGNSKHDPSKVPFSYPREYAESMSYDYQFFPNYMANTQS
SKAKVRSHSAPKQRPAETYERQLSRRRPSIEGRNVPRSV
KMQRSSSHVSATAQNYQYPWSIKLDKSAVSLKDSECGSNSTMLTNAHYCRSLVGFDVQGHRY

>DCAR_028654 (DCARv2_Chr8:3416380..3417848)
MGKATRWLKGLFGMKKDKDPAENSTSGDRKNKNGQNVGHSGRGSTGLLHNHATIPPNITPAE
VSWLKSNFFNDSDSEQTKHAIAVAAAT
AAAADAAVAAAQAAAEVVRLTSRANMLGGGRERTAAIKIQSLFRGYLSRKALRALRGLVKLQAL
VRGFLVRKQATATLHSMQALIRAQATVRAQRGSAFI
RKDSERFQSLFRARKSIEKCDETRSEHATSVHSRRLSGTFDNTIDESPKIVEVDTGRPKSRSRR
SNTWVSDCIDDPHSQTLSSPLPCRVPSRIWIPDCRN
CQESDWGLTGDECRFSTAQSTPRFANSAGSNAPLTPSKSVFLDGFFRQCTNSPSYMASTQSF
KAKSRSQSAPKQRPEAGQGSRKKLSLHDMMESRSSLSG
VKMQRSCSQVQDVVTFKNAIMGKLDKSADLSKF

>DCAR_016186 (DCARv2_Chr5:1070706..1072329)
MGKTTRWLRGLLGMKKEKENVDTFDLNDQKGKKKWSFAKSGKNSRSANQSPAINPATDSSW
LRSYTETEKEQNKHAIAVAAATAAAADA
AVAAAQAAAAVVKLTSQGRPNLRMSGRERWAALKIQTVFRGYLSRKALRALKGLVKLQALVRG
YLVRKRATATLRGMQALIRAQVAVRSQRARRSFNKDH
LSQLEFIQRKSIEKFEEPRGEYHSKRLSASYETSIHASEESPKIVEMDTLKLKSRSRRMNFSASE
SGEDRCDQTSSSPHLCLNPSRLYIPDHRQIHDYDW
GFIDEQYKFSTAQSTPRFAYSGHFKTPATPAKSVCGESFFRSYSNHPSYMAKTQSFRAKVRSL
STPKQRPEDVQKKRLSLDEIMASRTSFSGVKMQRAVS
HIQEYSNP

>DCAR_009937 (DCARv2_Chr3:12711029..12714673)
MYYVVDLIYRIDGFRLVTSLPYQKSKASTDKRGWSFGKRSARHRVLSNTVISEIPSSDSKEIPEP
ACADYQIQTRAAIPEKSSDVERTE
EMLQLSASINSKFPESVAVAEVDTKLDIQPDESVVTVIQTAIRKVLAERELSKQKNIVKLQAAVRG
HLVRRHAVGSLRCVQAIIKMQVLVRARRTRLSAV
ESTDEEKLHEKLEKYNHTQKVDRNSGAKPDLAHTSIEKLLSNRFARQLLESSPRTKSMNIKCDP
LRPDSAWKWLERWVSVSPLEIEQSPKSEPVTEIKEH
EVMKLPERHVETTCPAEEGAESTDLRYQGTMAAVPCFNPDTTEAIVPSEIEPNLVNQNFEQPQ
PEFLDLIKSTENLHQLPDKDKASDSSPQVEPKSLSSK
PDLEEQPKRSMKRVASEEPETEGRKFVYGSRKASNAAFIAVQSKFEDLTSASNSGKSVNISNQ
ESELKTCEDTVASVDDSFKARDTSLADCAVSSVTKVV
VGESECGTELSISSTLDSPDQSEIGAVDEQEVHIAQEAVENPKNTTNIVFEARVDEPIKLDTDLA
NPITTQLEKHITDEEVVTAESLKPDDRLEENTSDT
QIKLEHEAVNQMCKSSPEASPRSRATVQESQTTPSSQVSTKSKKSRSEKYGSSQKRKSLSVG
KRSPLPSNDSGVRSSLELLPKDQRSGKRRNSFGSAKAD
QVDQEPRDSSSSQSVPSYMQATESARAKAYANISPRSSPDVQDKELYIKKRHSLPGTNGRQG
SPRIQRSMSQAQQSTKGNGSNPSQGIF

>DCAR_023082 (DCARv2_Chr6:2023194..2024814)
MGKASRWLRGLLGMKKDNKDSADNSGMSDKKGKKKWSSAKSGNESRAANQSPVINRANDG
VWLRSSDKEQNKHAIAVAAATAAAADAAV

AAAQAAAEVVRLTRQGRQNVYGGSRERWAALKIQSVFRGYLSRKALRALKGLVKLQAVVRGY
LVRKRATATLRSMQALIRAQVTVRAQRARRSFNKDHPS
QPEFLHRKSIEKFEEPRSEYPSKRLSASYETSFNTSEESPKIVEMDTFKPKSRSRRMNFSASES
GEDTYYHSSSQPLPHRLLIPDHEQMQDYEWAFITDN
YKFSTAQSTPRFANSRHSNAPATPPKSICGDSFFRPYSNYPSYMANTQSFKAKARSHSAPKQR
PEIGQKKRLSLNEIMASRTSFSGVKMHRPVSQGQEDL
NF

>DCAR_022438 (DCARv2_Chr6:12422950..12427584)
MGRSTKSCFKIITCGSDSVDRDDLQVSESKSSARGWSFRKRSASHRVLSNTVISEVPSSVNKE
SPEAVSADYQNQPKAGLPEKSSEMQW
TEEVPQLPAAINSKFLEAVVGAEVDTKLDIPPDESVVTVIQTAIRKFLAQRELTKQKNIVTLQAAV
RGHLVRRQAVGSLRCVQAIIKMQILVRARHTRLS
AEEKRHVKPGRDSSKVKEERKSGAKPGAEYTSIEKLLSNSFARRLLESSPSTKSMNIKCDPSRP
DSAWEWMERWVSVSPTEQSPKVEPSTALKDQEILKH
PENQVETACADKEDSELTDFTFVATSAAVPYLESDSEEAAVLSKSEQNLASQEFEQPQPESQD
STKSSESPQQPLDKPSDSSPEVELNNLPSKPELEEEQ
PKRSVEIVASEHTGTEGRKIVFGSRKASNAAFIAAQSKFEELTSTSNLVNSVNSCNEDNEVESG
DITFSSSIDDSVKTKDTNLADDAIPTAPKVLLGGSE
CGTELSITSTLDSPEQSEFGAVDGHEVNISEEEMSNLKNTTICIENEARVDDPSIILNTDLSNPLM
VQSEKQSDTDIVNEESSKLEDRSVEIASSMQKKM
EHDTVNQMYKSSPEASPRSRVTILDSQTTPSSQVSTKSRKTRSEKNGSSQKRKSLSVGKRSPL
PSNDSGVRSSLEKLPKDEKAGKHRNSFGSAKTDHVDE
GRRDSSSSPSVPSYMQATESARAKAYANITPRSSPDVQDKESYIKKRHSLPSANGRQGSPRIQ
RSTSLAQQSTKESGNVPAQGMF

>DCAR_027238 (DCARv2_Chr8:24917852..24919493)
MGKASKWIKNLLTGKKSSSHQAVLVDSQQYPKTPVSESMASAKDKRRWSFRKPSSTTETYLD
AISNVKITVEAEDEQKRRALAVAVANA
AAADAAAKAANEAVMRLTCSSSPWTATTLVKNIAVAAEGSAQPADEAVRRLTCTSHRVATSVE
DTAATKIQSVFRSYLARKALFALKGLVKLQALVRGHL
VRKQATATFRCLQALLTVQARARARRIKMINEPNPYNPEDDHIKIVEMDIGVPRGRAKNIIGYFN
NKPTDNRYSTYQHQYSKQNHQQISPAPSAITDMSS
KSKSGHFNDFFHKTSYRSPQYNPSMDNYKPHQSKVHFSYKQENDRCYDYQFSPNYMADTKS
SKARLRSQSAPKQRPADTFQRQPSRRRVSLEGKNLQRAV
RMERSSSLVCSTPQNYHPWSELDQSAISMKDSECGSNSTLLTNCRHYRF

>DCAR_004404 (DCARv2_Chr1:47822568..47823538)
MQALVRVQARVRARRVRMSMEGQAVHDMLNERRSKAELLKDAEEGWCDRRGTIQEVKAKIK
MRQDGAFKRERALAYSHAQKRKSIQSSN
SQTSLLMASPKNSLGWSWLERWMSAKPWENRLMNKVESDPLEVTPPPKAYINDNIKASISKSS
EPCSLKIRKNNITTRISAKPSITEQSTRSSSSPSSEF
HCKSSASPSFCTSTTLSRNKNMASNRTGDNSVSKPRYMNPTKAATAKQRYMSPTAVVSRG

>DCAR_007588 (DCARv2_Chr2:35587408..35589120)
MGRATRWLRGLFGMKKEKANADSSNLSDKKDKKRQSFAVILEDSGFVDQTAAKNHASNSTSS
KSYTSESGKEQNKHAIAVAAATAAAAD
AAVAAAHAAMTVVRLTSQGRGAVSPSGREICAAIKIQAVFRGFLSRKALRALKGLVKLQALVRG
YLVRKRAAATLYSMQALIRAQASIRSQRARRSFKHE
QHCQPVTRRRKSAERVEDRNEFHSKTLSASFDNKYTAFEESPKNVEIDTFKPKSTPRRFPTSS

PESGEDPYFQSSSSPAPCALPARVYIPDHRHVQEYDW
SFMSDEYKASTAHSTPRFANSAYSKIPATPPKSLYGDSFFRPYSNHPSYMANTQSFKAKVRSQ
SAPKQRPELGQKKKMSLSEIMASRSSFSGVKMQR

>DCAR_026363 (DCARv2_Chr7:35717480..35719220)
MGRATRWLRGLLGKKKEKENVDNSNLSETNDKRRQSSVKAAKDRGVVDKTRGKNNYASDSN
RSRSYTSESEKEQNRHAIAVAAATAAAA
DAAVAAAQAAMTVVRLTSQGRGVVSATGREKWAAIKIQSVFRGFLSRKALRALKGLVKLQALV
RGYLVRKRAAATLYSMQALIRAQASIRSQRARRSFNY
DQHCQPVTRHRKSIEKFEERHEFHSKRSSASYETKYTAFDESPKNVEIDTTRSRSRRIVTSSSD
SGEDPYFQSSSSPVSSAVPARIHIPDHQHFEEFDWN
FIGEKHKLPTAHSTPRFANSEYSNIPATPPKSLYGDSFLRPYSNHPSYMANTQSFRAKLRSHSA
PKQRPEQGPKKMSLTEIMASRSSFSSVKMQR

>DCAR_020362 (DCARv2_Chr6:32009503..32011328)
MGKNPAAGSSWFNAVKKAFRSPTKSIKSSKRREEHEQEDDEKKRGKRRWIFSKQCIYETTIQH
YAPASSVVDTHLTVKPKALVDDYERR
DIASAEAVAEVASVQAAVEVIRLSSPSKITGEYFHAAIKIQSAFRAYLARRALRALRGIVKLQALV
RGQNIRKQAKMTLRCIQSLVRVQTRVRDQRRRLS
YEGSKDSTFNVSNSLRDYHAGTRKSRSREGSSITDEWDCHQHTLEEIKAILRKTKEADSDHRN
QLSTALSLRSLRPGKDQFLCKEGEEEKPGWSDQWKRI
GRNSGDQRDPMKTVEIDTAQSYSVPSLRRSHNSHYQYPQLDPGSESDCSPMHRTSRYLSGA
FPITPFQSKIKQLQVHSASPRCLKVERNIMMAQSPNMSS
NYHKMAGQENAVAASVPNYMAATASAMARSRSESTPRQRPLTPERGSVTSVKKRLSFAAPEP
DACAVMSDTELERNLCSPSSRYGMRGESNALLRGQPW

>DCAR_016970 (DCARv2_Chr5:9841834..9855217)
MASRKRASSNLNTTSVSTATQNNTTTHSTHTTLLPPTKASNMLKLSLLLFLPYFYLIFYHYGNIID
AQLKRSILINAGLSIFGFFVTIR
LIPVASKYLLRRNMFGYDINKKGTPQGSVHVPESLGIVVAAVFLVVAILFQYFNFTSDSNWLVEY
NAALASICFMVLLGFVDDVLDIPWRWNCKSRKLVL
PSIAALPLLMAYAGHTTIIIPKPLVSYFGVEILDLGWVYKLYMGLLAVFCTNSINIHAGINGLEVGQ
TVVIASAILVHNIMQIGASTDPEYQQAHAFSIY
LVQPLLATSLALLCFNWYPSSVFVGDTYTYFAGMTMAVVGILGHFRFDPQTGLLTGTTDGTLVN
LSLRLFGRMSEKSLCIVLLVLQFLMGKKKSWLSAVK
RLFIPEGKREEDKKSKRWGWFFGGIKTKQLPAISVPQRTLIEANEEQRKHAMVVAVATAAAAEA
AVSAAHAAAEVVRLTSTPSNFGKTTRNLAATKIQTY
FRAYLARKALKALKGVVLLQAVIRGQNVRRQIITHMKSLQSILKPQSQVHQIHVPTSEERCVHGN
DIQSFSPKKMLLGCKSSTTWDYSLFTEEDMEVSWL
PKKDGSAKRDRMKKSYSHRERPNNIVSGEYSNKEDGGKRPWFEELQDVKIRAAKKGQKFKEK
TESNFIDIVSTGRTQHKLKNVVKGESMEVLNSSLTLPR
RSFSRTNKKEYSIVDDTSLPHSPVFPAYMAATESAKAKIRSASTPRQRLAHIDFSSVHNSLYKPV
LSSWTSWDGELPEFRGKGATSLHKSQKL

>DCAR_008974 (DCARv2_Chr3:3191499..3193111)
MGKNNTSGGHSLLNVVKKAFGSPTKSTKSSRSEQHEQEADEKKRGKRMWIFSKPCIYETTIQH
SESANSGTAATCYHSCSSNASSRKLA
VNDVRPKADDLSHRRAIALAVATTAAAQAAIMTAQAAAEVVRFASSSEIARKYCNAAVVIQTAFR
GYLARRALRALRGIVKLQALVRGHNVRKRAKMTLR
CIESLVRVQTRVRDQRRRLSYEGSKESTFGVSISLREIHVANRKSQEKEEKNYGWSEYWEKMS
RKSCDKKDPIKTVEIDTAQCYSYRVPGSVCSPMHRRD

RNLSSPFPMTPSPCKTKRLQVHSASPRGLKEGRNSIMAQTPTLGSSYHRMFRHENGVSASVP
SYMAATASANARIRSESAPRQRPLTPERENVSHVKKRL
SFPSPKADVYGVMSDTELERRLSSPSSTRGMQFGTGRRSSTMLRKQPW

>DCAR_006559 (DCARv2_Chr2:26640080..26642359)
MGKKGASSWFSAVKRAFMSPTKENTKNSKRRESEEPNQEDEQKKKDKRRWLFRIPTQNAQQ
SQTKTAAINIPPANLSSFNADHKHAIAI
ALAQAHEAKAAAASVQAPVEYITLTRPPSYSLKHHYAAIIIQTAFRRYLARKARRALQGIVMLQAL
IRGQNVRKQAKMTLRCMTALLRVQARSREGSSIV
DSWDERPHTVQELEAILHSRKEAAFMRDTSMSSSFSQQSRRNTSIDEKDAGDRTKWLEQWM
AKKQWELNNRASAERREATRTTEVDTCRAYSYSASNGLQ
AQYMDQYQRQPSSCPVASPRYREPYSPSHHLPVTPSSKTKSLQVRSASPHRTKEDRRYSTAN
TPNLYFTPRASFGAARYSTCGNDGPVSAPVPNYMAATE
SAKARARSLSAPRQSRPSTPERDQRGAAKKRLSYPVVDPCINAGIGTSYSQNLRSPSFKSVQA
GYVGMGQQSCYTDSTGGEISPCSTTDLRRWFR

>DCAR_020520 (DCARv2_Chr6:30661972..30664070)
MRKGLGAFLFKGRKALMALKGVVRLQAVIRGQNTRRLILTRLKSLQPIAKPQVHRIIIPQVDKDYI
TISNPQKILLNKDKKTGCTIGKN
WEDSMMSKEDMEISWLQKIDAIARRDRMKKYSFSHRERRTNLTLDNSTDQDLKRRPWFEECQ
IDKSKSAIYSDLIDIATYGTRQHKMRYGGLKDEAPEVL
NSSLALPRRSFCDANIKKYSNTDDSSLPSSPKFPSYMGATKSAKEKVRSASTPRHRLGHDEPF
VVQNSVCYSSKLSSWSSVDGEIVGITRKSST

>DCAR_008876 (DCARv2_Chr3:2101369..2105774)
MGKTTRWLRSLFTKKHPPSTAEARRSVRSSGGPTSSSSSSVDANKHAIAVAAATAAVAEAALA
AAQAAAEVVRLTSGGAAQGGRCGPGY
GREARDQRKEWAAVVVQSAFRAYLARRALKALKALVKLQALVRGHIVRKQSTHMLQRMQAMA
RIQARACAYRVYTSESPQSSSKSSQSHQTGISSFETFD
HQVRTYGKRNESPLKRNSSKSGIKDNVRPESKNVGSSWLDQWMEENSRNRHKDTSSKISQA
DDEKSDKILEVDTWKPRMNLRQGGKVFQSSQHIPAWNKN
EVMFTPFDSLSRLSTQSQNSNPGSVRQEILHLKSLQMRHEVDQAAVWAVENSPGARSISSRP
GSSGSRRGPATPARSEFSQNYFSGYIGHPNYMANTESY
QAKVRSQSAPRQRMDFERPVSTRRFAGGYMDEETSSDRGWPLHGNINFRNRVHQDTGRSHI
TAMQYQGYEPTYGNR

>DCAR_018181 (DCARv2_Chr5:27212764..27215289)
MGRTSRWFRSLLGGKRPSGKRGGTVGNNGSVSSESSSYPHGLDASKHAIVVAAATANAAEAA
LVAARAAAEVVRLTATSATGRTRAADN
YAGMERRRYLAAVILQSAFRAYLARRALKALKGLVKLQALVRGHIVRKQSTYMLQRMQAMARI
QARACAHRAYTSDSPQSSNKSSHSQPPDLASLKAMNQ
HSRTHGTRPNGSALKKNVSKSNTKDNIGRGKTYNNRPNWLDKWMEENAGIKLREMSATNEYC
DDEKSDKILEVDTWKPHMNLNQNDQAYQRLQHVSAWNY
NEPIYIPLDALSAHSTKSQRPTRSQSPLEFSKISSVKVNHTAAGIAENSSGVCSATSRPGSSSSR
RGPTIPARSECSPSFYSNYLCQPNYMTYTESAQAK
IRSRSAPRQRLNSERPDAVKRYVNGYWDEEAISENGWPLHANFSTIAYPGTDSFEKFGYA

>DCAR_026389 (DCARv2_Chr7:35902541..35904090)
MGASGKWIIWFISRRKIQTTRDPARQALRALKALVRLQAIVRGRLVRKQAAVTLKCMQALVRVQ
ARARARACSESQASEDDLNDNGFIN
QGEWCDSPGTIEEMQAKLKMKEEGAIKRERALTYAIQQQQLRKKEFGLNSRKNKVATPKVSKS

SSGWNCLEGWMTPVSKHKQDYSVWPCSNSSESDSVKI
TRNNISTRISARPCITSPIIRSTSGPCSELFYDDSSTSNSSASTFGTPRSSNSQSERTTAKRNYM
NLTESIKAKQRASNHLSRNVKDHATEKLQLRKKSS
PLSKMLARRSSDCDMYSDDMEDLYPPLI

>DCAR_031045 (DCARv2_Chr9:32258293..32262562)
MPILGTSASTKENLPIKKEEATVSPKAEPVLPSPDIHNDENAPRNISPIDDSEKRRLEQAAITAQA
AIRGYQAREKLHSLEAITRLQAL
ARGHLVRRQAVATLQSVRGIIKIQALVRGQRVRRSIGSEIREREPHKEIDDQCLSSFGNIGSQRT
EMLLKTAFVGKLISSMPAAMPLQLCYEPEEPNSLS
NWLNRWTLLQVLGPHSQFKETIGVTGSSSLSVGTDKARSRGSVSRITSSDNDNGLKRSVPESE
RNKVKLKKVTKDPVKSIQEHPQNGTKKVLRNPKKVSK
PLVETSVKAVAGSERRKHSIGKSSKSDISEGISNPVEKVIEVSEKAESKPSDVEISQELPAEGTD
SKLDNHSYAPLQPSETPVQQKEEVTPVQQKEEVTP
VQTKEELTLVDEEIKKKNALINDENKKASMRRASLPAKHDYLEKDIHSTPRVPSYMAATESAKAK
LRAQASPNFDQDDAEKYALRRRHSLPSSNSGKFGS
SPRMHKLIQASIKGGMRGDRSLMSSRDSSDSRVIQAEWKR

>DCAR_025317 (DCARv2_Chr7:25970753..25972219)
MGKASKWLQKLLTRKREDKEENKDTSFSLEDAVLPAAVFPGTPNQKRRWSFGKSAASTDNNN
HKTSRSVDAIDGTILPLDLLEYRSEQN
HDANQLVPHNEASYRVVSARFAAAKQYRAIADAAATKIQAFFRAHLARKALKALKGLVKIQALV
RGHLVRKQTSEMLRCMTALMSIQVRARVQRIQMTED
PPIVVKRNLIHREPAHNQLRRGHSDNLNLSERRGSSRWNHQQLQTREHEFSTNYSERISVSKQ
ENHGRISVSKEESQFHVYPNQSPLTYTSCSQFEELFY
GTPDRISQRKYKVSNFSHIKAPESPSVDQKYMANTSRANLRSCSEPKQRPFKQKTVRSTSFGG
INTTPDNRDQNQSSNGKKKQPPWSIKRYRAAKSSKDI
EYDSNRIVTGDSSYYRTPTHTNK

>DCAR_004668 (DCARv2_Chr1:50220274..50222742)
MGKAAKWFRSLFGLKRPNNITITTPCTDAKRTPSRRWSFKRSTSNRYHTLSDSIVSALPRVHLK
GDKEQKHMIAAAVKNMSELKGNCDH
WAAVKIQAHFRGFLARKALRALRGLVKLQALVRGHIVRKQIAGRLLQMQAALRARTRARALRA
QIYEAPQSTIKPSHIHHLGPATPENSEHDIRRSRSSK
YEQSVMLEKNGSITASKVGPDKGKLNVRYSTDGESQDRVYLQDSTYHQAYFYPLSGEEQSLS
PLKYTIDVDEAFCAAETSLHVNTSRRASFTRSGPFTPR
SDISKSSKSGYSDNSPSYMSYTASSKAKVRSPSLSAPHQKTLERSNTTKRLPINGGYGNMKSS
SVHNVPSTLHSKFVSKAYPGSGRLDRLGSPLGSNGVA
FSCGLLSSY

>DCAR_000592 (DCARv2_Chr1:702618..706101)
MGKATRWFRNLLGLKTTPSSTATAKPPPKRRWSFVKSTTPHHSPAPPQFYDHAPTDHDQQHA
IAVAHATAAVANAAVAAAQRAAEVVKL
TSGDINVFKGPEYWAAVTIQSHFRAYLSRRALRALKALVKLQALVRGHIVRKQDTERLLQMQAA
LRARTRARVLRAQIQETSQSAIKLSHLNHPGPATPE
KSEHAIRRSTKHEQSVMMKRNGSRTNFRVGPEKEKYNYHYSMDEGSWDRVYSTRSGSMDE
GKSDKILEIDTGKYHVTPKRRSLFQSSLYPAASDPNSHSF
TTSKDSIFHQAILSPSSGEVQSLTPLKYTNDVDEDSFCTAETSPRFLSSKGGSFTKSGPFTPSR
SDASRSCLSGYSDYYPNYMSYTESSKAKVRSLSAPR
QRTQLERSNTAKRLSLHNVYGDMRSSSTQRIPSALHSNFASKAYPGSGRLDRLGLPLGGDGIG
FSGGLLNKY

Tomato IQDs amino acids sequences (allele name)

>SlSUN1 (Solyc10g079240)
MGKRRNWFTFVKRLFIPETESTADQKKPKRWRCCFLRKFKLRKCPAITSAPQQTLPEAKGTPQ
QTLTEAKEQQRKHAFAVAIATAAAAEAAVAAANAAADVIRLTDAPSEFKRKRKQAAIRIQSAYRA
HLAQKALRALKGVVKLQAVIRGEIVRGRLIAKLKFMLPLHQKSKTRVNQIRVPTFEDHHDKKLIN
SPREIMKAKELKLKCKSLSTWNFNLASEQDSEALWSRREEAIDKREHLMKYSFSHRERRNDQT
LQDLLNRKQNRRSYRIDQLVELDAPRKAGLLEKLRSFTDSNVPLTDMDGMTQLQVRKMHRSD
CIEDLHSPSSLPRRSFSNAKRKSNVDDNSLPSSPIFPTYMAATESAKAKTRSNSTAKQHLRLHE
TLSGQHSPYNLKISSWRLSNGEMYDSARTSRTSSSYMLI

>SlSUN2(Solyc01g009340)
MKKKHVPSYNKHTQSQGTTTKTNDHQLSLVTHEGETLLTKCFIGKSIIGGPSWLGLMKKAFRSP
IKDDIKSIRRREEAQDQEEEEKARKALGALKGVVKLQALIRGHNVRKRAQITLQCMQSLVRVQT
QVCDQRRRLSCEGISCGSMFKEPKSILELHLNDKESNSMNQAYIPDKDGYDHLHALEKIEALLH
KAKEATKKRENTLAHAFSRKMWTSNKDEDSSYNAELDEDLRVFDLIDEKNRKSISRASCDQPR
DRIKNIEIDTACSYSDSDTEFWRLHHQYYRDHQQKFYSSYVVPSPLSHREDINGLLMTPPLKMK
NIQVHSASPRYRREEKSHHHRMALLSHRANVNSSPTAADQPSYMAATASARARERSQSTPRQ
IPMTPEREKTSSTKKRLSFPIQ

>SlSUN3 (Solyc01g088250)
MAKKKSWFNLLKTFFVSGSESRLDKGKRKRWVFTRLKIRRLVATSALSPPRERRQQKTEEKQN
KHAINVDVETINESNVDAENAKVYFKEDIESIHEPNNETLVLSKLTIHGEETKYFYLYERRIENLAA
IKIQTAFRGYLARKALRALKGLVRLQAIVRGRAVRRQAIATLRSLQSIVNIQSEVCAKRCDNQVK
STVHCQEKTLMDLGEKDIKIDLNSQKRWDNRYLSKEEANKMFSSKREAAIKRERIREYWLSHR
RSTESDINGRRRYWLEQWVDAQLAKRDDLKNVDTLFSARNREEFERKEMKPKPSLRQYHTDQ
ELVSPVYVPRRSFHHRRQKSTGDHDNTFMGSPSIPTYMAATESAKAKARSMSSPRLRPINTDV
YSEINSPYKYKLSPISSINSDATVTSRMGSVPGFSQRSPCLKGTPGPIKSTRSIKDLSFGSDGAF
ANWDRIGACR

>SlSUN4 (Solyc01g097490)
MVRRMHSLMVIQLRARVQRVQMTEEAHTPNSRKSQKVSSGNNQLTRACRIEKMDASIQEKGR
VQKNNSTKNRSAGMENGLSTSEARRFSVSRRSHQVLQTCPSPSTLSDMSTISYDRNIEDFSFK
TPEKGFEHCSNVSTTTSSKTPFSIPHSENPNSIFSSATLALTYMSNTESSRAKARSHSEPRQRP
NWSIIRKSKRTPSMDGITGIPDSRREETPTHSRRHNVPESHEAWLLKLYKQAKSIKHVKVDSASI
VSTI

>SlSUN5 (Solyc02g077260)
MGSADWLKNVIGMRKAKDGRSKKNKGTSANKKSNGCKGHNPLQKEPSKITNIVLIKNQREMGI
PIEDRAAIKIQTAFRAYLARKTLRRLKGHTRLQSLTQRPSVKKQASSALNYICSWNTMQNEIRAR
RVHMVMEGHLKRKKHENQLKLETKLQNIEIEWSGGPETMEVILARIHQREEAAVKRERAMAYA
FSHQWRANSNPVFGSGNNEMNKANWGWSWKDRWVAARPWESRALVHASPKKVNDKANKN
TKSTTSPIKKTPVSVILTSLNGKGTIKAKKSNEVAAQKVMSNEVKETSSEKQ

>SlSUN7 (Solyc02g087760)
MGKKSPAKWIKAVLFGKKSSKSHLSKDASGEKISSAKAPVGDLSIDSPSLDLPVQNFDNGGDQ
AGLEKGTSTDFACETASLSSATHDIEPPVNGTTSSDDAELKRQEHAATIAQAAFRGYLARRAFR
ALKGIIRLQALIRGHLVRRQAVATLRCMQAIVRIQALARGRRSRLLDPGHHLLRKYSFEELKYPE
QRQAKLTVYAFPRKLLVAVPTAMPLSLQYDEGEPNSAWQWLERWSLSRFWEPLPQPKKVVG
AKSLKKQGNKPSVETEAVRPKRSVKKVLTASNGDAHGVSSSEPEKAKRNPRKFSNHHIEPVQD

QPQNELEKVKRNLRKVSAALATSSERSETEIEKAQQTPNLAQAQATVSKSSAPDVVEQMMVNS
CEKTSDSAPEIEKLVESEAPLPVAVDEPTDVLHDHPTTEQQQPEDVNNTANSPVVNEEISSMD
DQTTKERIRRRKSLPTKQDNSENISQNTPSVPSYMAATQSAKAKLKAQGSPKVSDDGAENGFV
RRHSLPSSANGKFNSLSPRIQKPGQANGKSGNKTRPISSSKDEKVLPGWRR

>SlSUN8 (Solyc03g026110)
MGKASRWLRSLLGSKKSPSESSPPAKEGKNSKWGLRKSSNGTGRTTGAEIAYGNEDPPASPY
AEALDANKHAIAVAAATAAVAEAALAAAQAAAEVVRLTSGKRSTSAYGSSNTERRREWAAVKI
QSEFRAYLARRALRALKGLVKLQALVRGRIVRKQSADMLRRMQAMARIQARASANRNVTSDPS
HSSIRASRFEHPGIATPRKYDPQQYSFNCKYHGPNLKKSGSKLKLHESFGQDRSHLASQYIHH
WMEECAKNGYGDTSLKKNGGDHDESTDKILEIDTWKPSLHPKPSEKKSHNSRYSSWNDNAH
GTRTVNSMSKLLGNHMKPNPSISSGEVSSLRSLTFCQDTDQPAAWTVERSPGVHSTLSRPGS
SSRRGPSLSRSDCSRSLFGDYLGHPNYMSNTESYLAKLRSHSAPRQRVQFEKIGSTKYVDGLV
DADTNSEKSWRSLGNFMNKPKPGSGQSDRVGTPDHRSAVRSSSPFGPRQ

>SlSUN10 (Solyc03g083100)
MGKKGSGWFSSVKKVFKQSPKDSPDKKKETLDSKWQQPEPPEVVSLEHFPVGSSPDFISNGG
SNVDSSSTVAEDHNHAIAVAAATAAAAEAAVAAAHAAAKVVRLAGGYGRVQSTEERAATLIQS
YYRGYLARRALRALKGLVRLQALVRGHNVRKQAQMTMRCMQALVRVQGRVRARRLQLVQER
LESKLEEARRHAAEEQQHKHTSPIKKSGTECWDNRNQSMDKIQESSRRKFDAEMKRERALAY
AYAYQQKQQQLLHIDPHGEDAEYFGNEREKSQWGWSWLERWMASQPYQYSRHIVPRDSSY
VTPSTTSDMSEKTVEMDLITGPLDSETINSAHHTFDQAETSPYSARKVVHSKFDNVPSYMTPTK
STKAKVRNQGLVKHRSPPSAQWNVSKRKGPHYGLGLESSSGASTTAIYQASRSPSPKTTANR
SSGHANKWMATYSPESSGDDRASPLGAHPWRYNFS

>SlSUN11 (Solyc03g121760)
MGGSGKWIKSLIGLKKNQSNDSEKSCGKNRKWKLWRSASGGIAMAFSKGVKGGGNLGDSDG
SESSFLSDSALAAAIATVIRAPHKDFVVVKQEWAALRIQAAFRGFLARRALRALKAVVRLQAIFR
GRQVRKQADVTLKCMQTLVKLQSRVRARCNQTSVDGDATKGPLVDSQADPVKQAEGGWCD
SPGTVDEVRCKLKMRQVGAIKRERAIAYAQQKLRTNPSLNSRIRKVETLNKFKANGDSVWLER
WMANKPWESRLVEDFHTDASGMTPSSRKYEDYDAGSLTDRSSVSIRRNNMSTRISTRGPMSC
QIANSSSEPYTDYYQYDDSTTSHSSISTSETLGSNQTPPEDGHSKKPNYMNLTKSIKAKVKQRN
SNYLSHSMQRNSTDNLQVHRKSSPLSRTIARRSADCDLYSVDLCKDLYPPSNAF

>SlSUN12 (Solyc04g016480)
MRKRRGWFVFVKRLLFISEAKPKAEKKPKRWKWFLGRFKFKQCPPAIEAPQQTLTEVTEQQKK
HAVAVALATATAAEAAVAAANAAAEVVRLTIAPYELERKRRNAAIRIQTAYRAHLARKALSALKG
LVKLQAVIRGELVRRRLVSKLKFMLPFQMSKPKVYHIRVPTVEEYYESIEKKLDDSLKESVKSNE
IKLKCNSQRTWDFSLASKEEIDSLFLRRQEAFAKRERMMKYSFSHRERRNDHVMQDPSIIKEN
RRGSRLDQWAEIEAQRKAELFEQLRSFANSSSPLVGMNQMRQARKLDVTEDLNSPSSLPRRS
FSHVKQKSIGDDSSLPSSPMFPTYMAATESAKAKTRSMSTPKQRLILNETYSVQHSPYMLNHT
SWTSYNGEVKKSTKKSEISQKTAINIKGF

>SlSUN13 (Solyc04g050050)
MEAENEQNKHAYSVAIATAVAAEAAVAAAQAAAEVVRLTAAACSSGQSKEEIAAIRIQTVFRGY
LARRALKALRGLVRLKAMIQGQSVKRQATSTLRCMQTLARVQSQVRARRIRMSEENQTLQRQL
QQKHEKEQEKLKASSQSGDDWNDSTRSKEQVDANLQMKQEAATRRERALAYAYTHQPTRRN
PSKSTNQTFMDPNNPHWGWSWLERWMAARPWEDKCAMDKEPNSDEVAAESPASQSTAVA
AITHRDFHLDNRPSPTAHKQNRPPSRQSPSTPRSKTGRIRPASPRVSNVDDDSRSMASAQSE
RCRRHSIAGSSVRDDESLASSPSVPSYMAATESARARSRLPSPLGFEKIGTPEKGSVSSTKKRL
SFSASPGGVPRRHSGPPKVEI

>SlSUN14 (Solyc04g081210)
MGKSTASSCLKIIACGSDSVDRDELEAHPESKSSSDKRGWSFRKKSTRHRVLSNSVVSETPSG
NKDWPEAANANLQTQSNSTIPEKASVVQWADEKPQFSTVEKSQVSADEKPQILEDETPLVSVD
KKPQVSADEKPRVSTDAKPQLSVEVSVDEKPLISEEEKLEVSEDEKPNISIDEKPPISSEEKSLV
SDLVDAKQSESVIIAGFNDAKADVIPDEHALVIQTAVRAFLARRAQLKQKHITKLQAAVRGHLVR
RQAVGTLRCVQAIVKMQILVRARHTNRIAEESSIKEKLKGKENSGTKSEFTYISISKLLSNSFAQQ
LLESTPRTKSINIKCDPSKSDSAWKWLERWMSVASPGNQPSPQSELSADQQENEPIEHPSNLI
ENEVQLDSESMDFRQGEEASLSAVPSESDDNLITYDADSLDFQANIPFSPPQPQNVDEKTSRD
DTFCSIPTQHKEAKALPETVPNSFPANTEVEREDTHSLELSETESKKILHGSRKASNPAFIAAQT
KFEELTLAAKSTKDSSLPNHKTEDESSEDTFSTITNHSFGARDAAPSENSVPHSTRAQVGGSE
CGTELSISSTLDSPDRSEVGGHVFEQELPSNGGTDHHKSNGYPHIEDDSTNDLSHSDYVQAGR
EDPTDDAKHVDVMVSSDLSPVEQKPENNSVNVQIEQEARTDRLDKSSPDASPRSHITVPESQG
TPSSQVSVNPKKIRSEKSGSIPKRRSAPAGKKSPSKLNHAPGTTSSEQLSKDHKNEKRRNSFG
STKAGLADQEARDNSTSSSLPSYMQATESARAKAIPNSSPRSSPDVHNKDEYIKKRHSLPGSN
GRQGSPRIQRSLSNAQQGAKGNGTQSPQERKWQR

>SlSUN15 (Solyc05g007130)
MGKSPGKWLRSLLPGKKSSKSGTSKKSSNEKASVISTNAALSGSSVHLPLISEPVACNAGGIKE
DSNFEKGGVTNEVILPSIERDGDEQNTCLTLPEDTEKMRLEQAATKAQAIVRGYLARRAFLRLK
GTIRLQAAVRGHLVRRQAVATLYCIHGIVKLQANIRGQIARRSSIGCELITKQGLEKQDAKQLDY
QRANASKLARELSTNGFTTKLLASLPTGMPLHLHYGQEEPNSSQEWLVRWTISQIWQPRSKLE
TLPGKKHQNAEADIAMSKHSVRKVHSKKMQNGSNHSTSSGSEKKKSSHLVNSVLQNPGSEIK
KVKHSLKKTSSPILEKPVQSEVDTERKRQSHDKSSSMASDEPLKNSEGTVENSTNVAPPPVVV
DDTITHLDILSVSDTHHKSTTDAADQKSITDNREDDTPVANEDFCTNHDNNEGSESNKVNRRVS
LPAKHDVDASTPTTRKVPSYMAPTKSAKAKLKEQASPRFGQDVAEKNAVTRRHSLPSPMNGK
LSSSPSPRVQRLVQASAKEGIKIDRSLSSSRDGTDKMTRAEWKR

>SlSUN16 (Solyc06g052010)
MGKKGSGNWFSTVKKVFIKPSCKDYLPTDHKKEKLENQWQHEAREVVSVEKIPAESCSDLTIN
RESNENSSISSAEDRNHDIHVIEATVAAAEAATYTAPKTIKLDGHNHKSKEEIAATLIQSYYRGYL
ARRALRALRGLVKLQALVRGHSVRKQAQMKMRCMQALVRVQSKVRARRLQLRQSKVVEEVK
SRSSIREESLLLNKQETHSIENTRRKQQVYNAYQKWLHSDLDDEECFGNEHENPQQSWNWLD
KWMASQHVVRQEDSSYVSLSITDDISEKTFELGPEDVNLAHQNEKSPYSSTQSNKDSVPSYMA
PTKSAKAKIRSPGPIKPKSPPGVAQWNSTPKKVTARRWSYDSSARSPNPKISAKWMATYSPQT
RVHDRTSLVGPPAHRYNYN

>SlSUN17 (Solyc06g053450)
MGEKGSWFYALKRVFTCNSKKKSAYGSGKKTAKEKKKGRRILRHGEFKSFLFREPSSIEKILGE
VDDQNLLVRPPTSEQSGIPSAFPVAPTSPRINSPRDASPQSSYRATSPGVTSPRVASPRVVSP
KAASPKVTSPKEASPKVTSPREAYPKATSASAPSLKALSPREASPKANSASAPSQKATAPKEAS
PRVSSPRSTYPEPSRNHNEISYANRPEATLTLHLSATKIQAAYRSYMARRGFKSLSGLARLQGV
VRSRSVKRQTVNAMKQMQLLGRVQMQIQSRRSQMFQALHSQAYMNDKEVESTLSKWTQLTE
AGNHDDWNNSMLTKDEVEARRREKVEAVIKRERAMSYAYSHQLWRRNPKSATDIRTSGIPW
WWNWLHHQLLPGNDSESQSAVKDVHSTPSRAISEHKPSPWRLSQNFRHLHLDYDSHESVTP
RSTKSAVPLRGKLMHTPRRTSSPMSSSSVSKYSRRRASAADSPFNHSMKDDDSLTSCPPFSG
PSYMSPTISAKAKFRGKTILEERNIGTPSNSSRRRLSFPLTPSSTGSVKWNKGSGKDAASLKEH
ESMGDHMSVHSTGSTPTIVGRKPFQRFV

>SlSUN18 (Solyc06g066430)

MKKDKENVDNMSNSSDKRDKKRWSFGKSSKESIGVGDNPVNFPGGVPAVDSNWLRSYISEN
EKEQSKHAIAVAAATAAAADAAVAAAQAAVAVVRLTSQGRGAMFTGGREKWAAAKIQTVFRG
YLARKALRALKGLVKLQALVRGYLVRKRAAATLHSMQALIRAQAAVRSQRARRSMTNDTRNQP
ETRARRSIERFDEYRSEFHSKRLSTSNDTSYDGFDESPKIVEIDTFRTKSRSRRMNNACMSES
GDEQHSQAMSSPLPCPLPARLSIPDCRHLQDVNWSFLADEQCKFASAQTTPRFAGSGRSNAP
PTPAKSICGDGYFRAYANFPNYMSNTQSFRAKLRSHSAPKQRPEPGPKKRLSLNEIMASRTSF
SGVRMQKSCSQVQEEYCF

>SlSUN19 (Solyc08g007920)
MGKASKWFKALLGFKKNESSISSTNKKKWGDVKSYKDKDFQQHHEKSHYMNSRAGVDLAIYE
VHSSLTTSSVIRTTTWNSEEWAAVVIQSYFRAYLSRRALRALKGLVKLQALVRGHIVRKQAADM
LRRMQALIRAQSRARLGRSMVFESPPFSTKSTQSIHHGPTTSSRCTRSRTGPFTPTKSSTRSY
TSDEYSNNHPNYMSYTEAAKAKTRSMSAPRLRSQYDKKYARSNMQ

>SlSUN20 (Solyc08g007930)
MGKASKWFKALLGFKKNDSSISSTNKKKRGDVKSYKDKDFQQHHEKSHYMNSSAGVDPAIYE
VHSSLTTSSVIRATMWSGEEWAAVVIQSHFRAHLSRRALRALKGLVKFQALARGHIVRKQAAD
MLRRMQALIRAQSRARLGRSMVFESPPFNAKSTQSIHHGPTTSSRCTRSRTGPFTPTKSSTRS
YTSDEYSNNHPNYMSYTEAAKAKTRSMSAPRLRSQYDKK

>SlSUN21 (Solyc08g014280)
MGKKGSWFSAIKRVFTPSSKEKLPNESEKKGAKEKKNRGKLKHGETKSFIPLFREPSSIEKILGE
VDEEMLLSPRFTLPAGAVSPRISSYRFATPTATSPRVASPKASSRRVTSPKAPSQRVTSPRAIS
PKAHPPRVPSPNVSRNRKEISYAYRPEPTLRALNLSATKIQAAYRGYMARRSFRALRGLVRLQ
GVVRSSNVKKQTANAMKQMQLLVRVQTQIQSRRIQMLENQALQHQAYKNDKDVESTISKWTQ
LCEAGNNDNWDDSLLTKEEVEGRLRKKVEAVIKRERAMAYAYSHRLWKNDPKSGLDMGANG
FPWWWNWLERQLPSRNANKTPSAMKDIKLTPPRAISEHKPSPTPLNNVTFRRILSDYDNNESS
VTPMSTKSAIPMRGKQMHTPIRTPPMNNSSLKKYSRGRASASNYPFDLPLKDDDSLTSCPPFS
VPHYMSQTASATAKAKARANSNPKERNPEKQSNDTKKRFSFPLTPNIWSSKWSKGSGKDSAS
RKEVDKHESMADHISVDSTVSMPAVVGGRRPFNRFV

>SlSUN22 (Solyc08g062940)
MGASGKWVKALIGFKKSEKEDHEKKVKKWKIWRSDVKGLKQRNGVGSEGSDCSSMNNDAYT
AAVAAVVRAPPKDFKAVREEWAAIRIQTTFRGFLARRAFRALKGLVRLQALVRGRQVRKQAAV
TLRCMQALVRVQARVRARRVRMSIEGQAVQKILEEHRGKLDPMKEAEEGWCDSKGTLEEVKT
KIHMRQEGVLKRERALAYSQAQKQSKSSQNFDSRTTISVPSLKNLDYDKSNCGWSWLERWMA
ARPWENRLMEQAYTDSMETTPKSKACLETLKDKEKATTEPCSVKVKKNNVTKRISAKPPLI

>SlSUN23 (Solyc08g080470)
MGKASKWFRGLLGLKKQDPSSSSNQNPKPTKKKWSFVKSYREKDSNFVKGTDKESSNYGRV
VSNSLQNGAVGCSVDSSKRAIAVAEATAVVAEAAIAAAQAAAAVVKLTNSGRATTATNGGAAA
VTTWNGVSLTSSAVGCKRKGVAAGNRENWAAVVIQSHFRAYLSRRALRALKGLVKLQALVRG
HIIRRQTADYLRQMQAISRAQSRARAGRSQVSGSPHSSTKSVQFVHDPTTPEKFEHVIRARSLK
HDETSVLKRNTSKSNWKVIDSEKARIRPQGSSARTSSIDDEKSDKILEIDTGKPYVTPKQRNLFH
SSHLCLNSDQYSYSLTTSKESTAHQTVPSPSSCGNQPLSPLKFNEDLDEACFCTADNSPQFYS
ASSKGGSSKRGPFTPTKSDGSRSYMSGYSDHPNYMSYTESSKAKVRSMSAPKQRPHYERSS
STKRYSIHGYSESRNNSQKGSFYANFTGKAYPGSGRLDRLGMPVIRADPSGFSGGLRHRY

>SlSUN24 (Solyc08g083240)
MGKKKGATSWLSAVKRAFRSPTKDNSCDKKAKIEHQLDEDEEKKRDKRRWLFRKQSQSEGK
AMVDPKHAIAPEAAVATDQAALEIIRLTRSNKYSPSNNAAVLIQTAFRGYLARRALIALKGIVKLQ

ALIRGQNVRKQAKMTLKCMQALLRVQARVREQRARLSHDGGRRSMFAETTNLWDSKYLRDIR
DRNSRSRDGSSIADDCPRSLVELESMLQARKEASFKREKSLAHAFTQQELDEMDIVCSEERNE
RELEETANWLDEWMSSKQWNRGSFDRRDSIKTVEMDTAKPYCNMVPNARRSQHSSPLHRQA
SSPHYTANSPHHQRSSHYNYSAIQPPATPPPCQPKPLQMRSTSPRKSQSTANTPCLRSTSRS
NSIMSRYSTSGNDASVPNYMAATESAKARIRSQSTPKQRPSTPERERVGAVKKRLSYPIPEPYT
GYGYSQNLRSPSFKSLQAAYVGMEQQSCYTDSLGGEISPCSTTDLRRWLR

>SlSUN25 (Solyc09g007410)
MEKKGSWFSSVKKALSPDPKEKVDKKASKSKKKWFGKEKHTLVDSSTAVTATASPPHPVPVL
PVEEVKLEEVEEEQTKHAYSVAVATAAAAEAAVAAAHAAAEVVRLTTVNQFSGKSQEEVAAIRV
QTAFRGYLARRALRALRGLVRLKSLVDGPTAKRQTTNALKCMQTLSRMQSQISSRRIRMLEEN
RTLQRQLMQKHVKELESLRRGEEWDDSLQSKERVEASLLSKYEAAIRRERALAYSYSHQQTW
KKSSRSTNLLFMDPTNPQWGWSWLERWTGARPWESQSMSEKQLKTDQMSVRSVSIAGGEIA
KSFARHQLNSELPSSPSRQKPSHPSRYHSPTTPSKPTTSVAAARKLKPASPRISAMNQDDDNR
SMLSVQSERNRRHSIAGSSIRDDESLASSPSVPSYMASTQSAKAKTRLQNPLGMENGKPEKG
SAGSVKKRLSYPPSPARTRRHSGPPKFDNTSLNTSIAEDHVNGVVN

>SlSUN26 (Solyc09g082560)
MGKTGRWIKNLLIGKKDKDKDKDKLEGEKIQNSITSNEHQPTTPISIPSTTPKDKKRWSFRRSSA
TPPGQRDLSGTDIVATTTAKQELLESDNDHKKHVLAVAAATAAAADAAAAAAKAAAAAIQFTAA
ARAYALEEDAASKIQAVFRGYLARKALNALKGLVKLQALVRGHLVRKQAAATLRCMQALVTVQ
ARARAQRIRMTEEENPNNPRQSVHRKSTQDNKFRHSYQDYEEDIKIVEMDLGESKGNTKSRN
CYSNQGQTERTEHRFSTHNAYTNQEHHQHIISPVPSAITEQSPKAYSGHFEDYTSYPTTHSSP
QYYNNTMSKPDPSRILYSSYARSEYAEPESLYNEYPFYPSYMANTKSSIAKARSHSAPKQRPD
QTSFERQPSRRRPSIEGRNVPRAVRMQRSSSHVGSTAQNYQYPWSIKLDKSNISINDSECGSN
CSVFTTHTNYCRSLVGFDVQEHRY

>SlSUN27 (Solyc10g005000)
MGNNKKGSSSSWLSAVKRAFRSKKQINEEEKKKENQRKQQHTTNHNAAAQAATIIQTAFRGYL
ARRALKALKGLVKLQALVRGHNVRKQAKMTLKCMQALVRVQARVLDQRFRQSEQASRKSDTT
TSHQHMSSTIPDDWDERPHTIEEVKAMLQKRKEAAFMKCQGTPFSQQTRRSGRSSSIGSDAD
FGEKRAAAKSWDSSSRGRASTYPVEMDTSQRNLITRLHQRPTSPLHTSFQFPVTPSPSKSTRP
IQVRSASPRHDKKSQTPSLRPNNNYSSYQPNRLTSSAAAIPNYMAATESALARIRSQSAPRQR
PSTPERDRAGSAKKRLSFPVPDRYGNVPSAYGHNLRSPSFKSLSGVHFGYEQQSNYSSCYTE
SIGGEISPSSTSDFRRYLR

>SlSUN28 (Solyc10g008790)
MGSGYWLKKVISLRKAKDGRSKRLKGTSIGRKEDEHSQKEPSRRTNGASVKKHREMGIPSED
NAAIRIQTAFRAYMARKTLRRLKRISRLRSMIQGPSVKKQASTTLSALHSWNRIQAEIRACRVRM
VIEGRLKQKKLENQLKLEAKLHNLEVEWNGGPETMEVVLSRIHQREAAAVKRERTMAYAFSHQ
WRANSNPMFGSSIHDLGKANWGWSWKDRWIAARPWESRIPVQSSPKIANRTASKTPKSYKTQ
TTKTPVSVKSTSANRKRAMKPRKLSYEAADKLTALKGINKVETSIDKQEVAS

>SlSUN29 (Solyc10g084280)
MGKKGSWFSSVKKALSPNSKEKKDGKSKKKWFGKEKQPLPDSSTLVVASVSPPQPIPQVEEV
KLAEVEEEQTKHVYSVAVATAAAAEAAVAAAQAAAEVVRLTTVNQFPGKSKEEIAAIRIQTTFRG
YLARRALRALRGLVRLKTLVDGPTVKRQTANTLKCMQTLSRAQSQISSRRSRLLEENRTLQRQ
LMQKHAKELESLRRGEEWDDTLQSKEQIEASLLGRYEAAMRRERALAYSYSHQQTWKKSSKA
TNLLFMDPTNPQWGWSWLERWMGARSRENQNMSEKELKGDQMSVRSASMSMSGGEITKAF
ARHQLNSELPSSPLSQKPNRPSSRQSPTTPSKPSTARKPKPGSARVSAINQEDDTRSVFSVQS

EMNRRHSIAGSSVRDDESLGSCSSVPSYMASTQSAKARTTRLQSPLGVENGTPPAKGSAGSV
KKRLSYSPSPAITRRHSGPPKVEITSTNTSNAEKYVNGVVN

>SlSUN30 (Solyc10g086060)
MGKKGNWFSSVKKALSPDPKKSKKKWYGKEKDPVPDSFSPVAASVSPPHPVPHVEEVALDEV
EEELTKHAYSVAASTSAAAEVDVSATEAAEEVVWLDKVTQYTGKSKEEVAAIKIQTAFRGYLAR
RALKALRGLVRLKSLADGPTGKRQTAHTLKCMQTLSRVQSQISSRRIRMLEENRALQRQLMQK
HAKELESLRRGEEWDDSAQSKEQIEASVLSKYEAAVRRERALAYSYSHQKTWKKSPRSANLLF
MDPTNPQWGWSWLDRWMGAKPWDTQSMSEKEHKNDQMSVRSASIAGGEITKAFARYQLNS
DLPSPSSQKPSHQSPTTPSKPANSTASRKLKSARVAAISQDDDARSMISMQSERNNRRHSIGV
ASIRDDESLGSSSSVPSYMIPTKSAKAKTRLQNPLGMENNSTPEKGPAGSVKKRLSYPPSPAR
PRRHSGPPKLENTSMNTSINTSIAKDNVKEVIN

>SlSUN31 (Solyc11g071840)
MGVSGKWIRALVGLKKSEKSHSSEKEENKSGGTGKFWHRRKHSVEIDSNLLQKELTYNDAGA
GSVEDISSTSAPFASSSPSSSHQLHHAPLVKQNMREELAAIRIQTAFRGFLARRALRALKGLVRL
QALVRGHAVRKQAAITLRCMQALVRVQARVRARRVRMSLESQTEQQKVEQQLEHDARVREIE
EGWCDSVGSVEQIQEKLLKRQEAAAKRERAMAYALAHQWQAGSRQQATLSGFEPDKSSWG
WNWLERWMAVRPWENRFLDLNVRDGVMPNENESAEPVNGMKNQVKVAGKKPATTTLSNDR
VGPSHSSSNSKSNEKAAASLSDGCSSSPNVSASTQETPAALVNKPKSKPNREDLVEEASSKPV
LGSRSHSNPKERSTPSDKQGKQRLSLPGSGLAPQTARQPSRTIKRTSSTQKPLKEKSKLNETD
TKSTATVSQQAAD

>SlSUN32 (Solyc12g008520)
MGKAIKWLKGLFGIKKLEIDEKGKIGTTCFGHSGRDTTAVAAVSDRLCHNPTTIPPNITPAEAAW
LSSFYGDESDKEQSKHAIAVAAATAAAADAAVAAAHAAVAVVRLTSQGRSSAVFGREKMAATK
IQTVFRGFLARKALRALKGLVKLQALVRGYLVRRQANATFHSMQALMRVQASVRALKINGGFY
VNHQNSQFQQRKSIEKFEESRSGSTKQMSSRRLSSSFEANNINEESAKIVEMDTGRPKSRSRR
TNTWASNPCDDPFEQVLSSWASDWAQQPVGTAQSTPRFANSCGSNTPTAKSVCVESKYFRN
NNYYDTNNNYYPNYMAKTQSFKAKLRSHSAPKQRPELGSRVKKMSLNEMMESRVSSSGVKM
QRSCSQAQEKISINFKNVVMSKLGNSTEGERDF

>SlSUN33 (Solyc12g014130)
MIGLKRNKRRKSKQLKITSTSTAFDEPKGDVQVPSAHYSNGTSSSKKITCKTKFTHNTAATKIQT
AYRAHLARKTLRRVRGAVRFQGVIEGLSVNNQISGTLKQIHCWSKIQSEIRARRLNMVTQGHNK
QKKIQNQQKLEAKLHELEVEWSSSAETIEEILQKLQQREEAATKRERAMAYAFSHQWRANSNK
YFGQAYYDLGKESWGWSWMERWIAVRPWETRVQTNPIVPKTSHSQQVAKITSKATNLGPMKL
VVSIKNH

Tomato OFPs amino acids sequences (allele name)

>SlOFP1(Solyc02g085510)
MGKSLKLRFSRVIASFNSCRSKNPSSLPQNPNFFPHKLTSTKHISPDFPLIDQNQNQNHRNYVP
ESTMISVGCCRSEFKWEKEEKFHVVSSSFVSEEEECEEEINLALRPPLTPPRFSRIVVEKKKKK
QQRVKKTKTKSRIIRMSTSSADEYSGILSGTNTDWDNNEEETESLVSSSRSCYDFSSDDSSTDF
NPHLETICETTTMRRRHKRNANTKRRSIKQSRPSFSSSKGRRSSVSTSSDSELPARLSVFKKLI
PCSVDGKVKESFAIVKKSQDPY

>SlOFP2 (Solyc01g007800)
MSTHRRRIILSNVTVKLGCSSSCIRPKLSSIFHPKPRKSPKSQTQNKNYSNYSSCSSWDTTTTTF
SPNSDSTTNESSDFKTSKAVQGFGRIGGESVAVEKDSDDPYLDFRQSMLQMILEKEIYSKDDLK
ELLNCFLQLNSPYYHGIIVRAFTEIWNGVFSLRPGVAGASSPFLHGGSHVTYR

>SlOFP3 (Solyc01g007810)
MKLSSLFKNSSQNSSSTTTTTPWPWSLPTCGKPKTLSFRLEKNQHNIYNSTFHLDDINDTTSCS
FDDFFSEIDETSSSSTTTINGQDCIEKVIKGLRLEKERLFFEPEETSSILDFQENKNISITSSNININ
VVDEGNIISFVPMGLDSNDPFVDFRKSMEEMVEAYEIKDWENLEELLTCYLKVNCKSNHGYIVG
AFVDLLVNLATFSDNNNNVGVDIGAGVGAGVDESTIIMTTIDEEQQCLSSSTTTTTTTTNHSFTS
PLSFCSSSCSTSSSITSTSACLSLLLEEDEVIQTKKH

>SlOFP5 (Solyc02g072030)
MKWGKKKPSSSLMTHVFPVSWLSKFKQKKVCRSEDQEGAKMRKVDLRTNVCLKQGRFYEDD
PYWRISFSEENHPQNPLWCGECDQNSKSSLGEENHKFNDMVSRKISEKPKNEAEFSNRKRNS
VKDEKLRKLSRKALEERIAENAREEVTEKDIFEIEPEDEKVMKRGKEKPTAYKSRKARSLSYND
SSPNSVEESCMMFTSLNLEEEADALSEEEFESECLKIKEMSEKSGCQQRKSVYINQKRRRKHG
IKVRAYSPRTAKMECRIKALEDMKKARMKTRHETKESFTGDRTVFDSYAIMKSSFDPFSDFRDS
MIEMITQRGIKSSEELEELLACYLTLNCDEYHDIIIKVFRQVWFELNQINIGEELQKCCCSDE

>SlOFP6 (Solyc03g034100)
MAKLLKFRISKAISNSFHSCRSKDPCTLPQHPVPSFLQNTQFITDDHLLFEEMIQQNNESQLIST
HHEHFPIITSPSFKHHVSVTPITATGQCSSRNGEAFSTTSDDSHTRSPSHEFKWKKEEDKWQH
FIKTNSDDDTKQQPRRKISYSFSSDNSDNDKILIEIKKKISTSKTNFFMMMSTTSSSSSMDENEIN
FTSKKTKWDYHEDIDITNEDEENETETFISSSRKSHVEFPDDSSLNFSHEFDTIYKNTTRRCQKK
IGYSKRRDHVKNTRSRSSRDMNNIGRRSSISTSTTSSDGELPPRLSVFKKLIPCNVEGKVKESF
AIVKKSEDPYEDFKSSMMEMILEKKIFEKNDLEQLLQCFLSLNAKNCHGVIVEAFSEIWETLFSP
NHN

>SlOFP7 (Solyc03g120190)
MPKQLQKSLSDYLTKKKKKATAQQTTNSANKTLSSSTSWLLRGCRHPKTPSFSAVDRKEKNV
QGENEAATLADVDRFVFENFKSFYYKDDDNEAEIVENPNSLSESPRHIIPPLNHTGSRRFFIAPG
SSSSLIEEARTSMTVSDDTGSTSAITITTVTNTNSNELSAISTEYSKETLNANDFITLVTYSPSPYD
DFRQSMQEMMEARLKDQGKINWEFMEELLFCYLDLNDKKSYKYILSAFVDQIVILRENSGRVPA
ISRNVRPLDGELNQRDT

>SlOFP8 (Solyc03g120790)
MEESYKKIEPQMLLKKTIQKTKNFLYRTPHNLKSFLFGGHHKLPKTACHFNPFLSVSKRFSSSK
RIPKTNVKELDDLYRDYYQQWNQPDHNEIQERKMTSKNARKFQGMAEGDYSESQRELAVRFG
MEDIESERRKEDEKKGREVLTRSTSKGSLTLLKKMEELEMVEGEDMDHVLDIEEVLQCYTLLNS
PVYVDIVDRFFMDMYTEFSIRKPSGSVNSSMRRLGPLKL

>SlOFP9 (Solyc04g080210)
MTRRFKLKLSMPSFRFCRPKKASFLPKSPMPLSLYKFSPANILDNSPVPVPPSTPHHPYILRKA
HNLASKTYNSPSSEYSDPDNNNMRRGESRKSRLNMSFSSVDSGWFSFNSECCDEKPNDETE
SFMSSPSFESSFDVDHGIDPLSGIRRKKNNNNTKVRRLRRYLSNSLKDSMMPCMADGKVNES
FAIVKRSVDPYDDFKNSMKEMIMEKEMFEAEDLEQLLLCFLSLNSRHHHAIIVEAFTEIWEELFG
KSSKSMDLKLPRFQ

>SlOFP10 (Solyc05g055220)
MAKKLKISSIFKKKELGLATWQWPSCTHSKTLSFRGDDNIFKTINSVFFDPFDGIETPQSYSTNS
SLDTNSISIESHEEIIKGARSERLFFEQVATSSIFQEPQEENQENDLPFKESVILAMESKDPYLDF
KKSMKEMVESQGIKDWDNLQELLACYLKLNGEVNHGFVLGAFVDLLVELVIPTTPSTNSDNSIT
SYSSVASSSFSCPSSPLSSLGHKETEEQENAKVS

>SlOFP11 (Solyc06g073040)
MVNYKGGKLLKHQRNRVSFSAKLPEDVRGAFADSTCVVKYSMDPLTDIKESIKEMVKNVGIKD
WKEMEELVYCYIVLNSEETM

>SlOFP12 (Solyc06g074020)
MTRKYDQCLVDNMFGPFPESCCPDEALEMAKQALATRRLSFEENESCSVLSMVGFPFKDCLL
LAVETENPKMDFLHSMEQMTKVYGAQRGDMVDWEFMEELLTWFLKINNMKNQHFIVAAFIDLC
LGGHVQDVEPVENVEPLTDDIVNVILEELWWGL

>SlOFP13 (Solyc06g082450)
MNTSCCLKFNNPCKKIVKLFKFKLRKPLFIRRLRIFRPSTRCESNTSTRRKQASQVLSVFRFIRR
SKPREEDQVMALKSFSGHIKAPVPSPITPAYARLSGATKKEVVIFQDDVEDACRSFENYLAEMIV
EEGKMRDIMDVEELLYCWKNLKSPVFIDLVCRFYGELCKDLFSHTYKDDINSPQKIMQ

>SlOFP14 (Solyc06g082460)
MGNHKFKFSDMMPNTWFYKLKDMSKTKNHKSPFSSSSTNKSQYSQPRSSFSYTRRSIRVDKI
YNSHSYNFLDQPRRSSSSSSSKKKSKRKTIYKPSPKHIPSSVTNYVSVSNKLNTSSSVYSTEEDK
FPELDFLNSPSSEFDSVDSQTFNELPSTWPNSCNCHFTSSATDIIIDVNDKALSNEFHNLTTEYA
EFSDIDQLPPIFTKASNSIKNIKQDENVKAQREKEPKNRVGSPVSRKHYSSSSGVKLRTNSTKV
ANKRNSVSSSKRRSKTKKESCSASRGTSFAIVKASIDPEKDFRESMVEMVVENNIRASKELENL
LACYLSLNSNEYHDLIIKAFEQIWFDLSDLHL

>SlOFP15 (Solyc07g055240)
MQNSKAAVAAAAKKQKLKGCSALCCSCRLSVSSSSEEAESSSSSRYPTISSLTHAMVQERLDK
MIREREEAKNEEMRRRRRRAERDEKTKFIVMIAMEKSSYDPREDFRESIEQMIIANRICDPKDLR
RLLNYYVSMNAEEYRGVILEVFHQVCTTFFLSCKQPSSQV

>SlOFP16 (Solyc08g068170)
MDNHIVNKFSRLFPSSFTSCQFRNIPDVVENSFIISKNSKFVHNNAVGVSSRTNSSIKTQESKSN
KNTLLKSSSSNSGWFSSEGENDETEDAFFSLSSGYFSDSFRRKPDESCRKMYNQKSSEMGR
CYSELSVNSVSRVGSNLGESKWVKSRNGTSKTEQNGGKVSRTLRETGGTSRKKYGSEMSHC
SATLSRTNGRVGSNLDESKWVKSINGLDFASAKTEQNRGKTSRTARKTANQNDSEMSPSSSA
LWRTNERVHSNLDESKWVKSINELDFTTSKTEQNRGKAESKWVKSINELDFASSNTEQNRGKP
IRTARKTANQNDSEMSHSSSELSMINEQVNSNLDELKRVKSINELDFTISKTEQNRVNTSRKTP
PRSSRRTLRKTTPVEKFDYYSDFTSNTRRKTTKCRRKIKKSSSTSSDEMGRIKIVIEGRIEESIAV
EKNTNDPHNDFRTSMLEMIVEKQIFGLKDLQRLLHCFLSLNSPSFHKIIFEVFAEIFETLFH

>SlOFP17 (Solyc09g018200)
MGNYRFKLSDMVTSSWFYKLKDMAKSRTQIKRKQTSSSTSSSSFSIFYSSSNVQQHHRKSYYF
SRTLSPNPHQSNVTPMKSSKKRKNTRRNTPKFVNSPKSMILSPSHRRCNDHIFDSVSKIDLPPIL
TKPNKKEEKTELKFLTVKTEQSTSPKRRISVSSSSTGVKLRTKSPRIISRRSVGEKSYAVVKSSK
NPQKDFKESMVEMIVKNNIKTSKDLEELLACYLLLNSHHYHHLIITVFKQIWFDLQLK

>SlOFP18 (Solyc09g065350)
MPRTTLGTNFNLCFTKLKRSLPLRSIDDNDNDNDNERHQQQHHSMHFCNSVKNFNSLYDLSS
SECNIPTSSSTDESDYNYELENNTPDLATIYASQRFFFSSPGHSNSIIDSSSSISSSIASTSSSVG
SDAPLEGASRFQRIHPTRIWIFDDRCKKWWRHVD

>SlOFP19 (Solyc09g082080)
MPRILQKKFYHCLPSFKCLPTILSLPFEETEEEKTEQKKIKNFNSVFDIPSSDSATTSKSLTNSST
TTTEEEDNNTNCTFTSFEDSDYTNIPDFSNIFASQRFFFSSPGNSNSIIDFPPENPKVVTGGVAV
QTYSPDPYSDFRRSMQEMVEAHELTNVKANWGFLHELLLCYLNLNPKHTHKYIIRAYSDLVVSL
MSMDDSEKKTEGIARP

>SlOFP20 (Solyc10g076180)
MGNYRFRLSDMMPNAWFYKLKDMAKSSSRRHSHTTSSSNLQLDKKRQPHNNLGCQRKSYYI
SRNLTITSPISSNSPKLDHNVHITEPSRKSYKKRRSTNFRRRNSPKPVNSSASVESVWTKPDST
PEQYPNSSSSSSSSSPSSILPHKSNPIASISPSCDCRTDYTNQNSANLDPGVHSVSKIDLPRIITK
PEKFNEKIQEKQRIVKQEQRIVRRVSTNGVKLRTNSPRITTTTTNSRKSVSSKRTSVTTDSFAVV
KSSRNPQKDFRESMVEMIIENNITTSKDLEELLACYLSLNSDEYHDIIIKVFKQIWFEITEIRLK

>SlOFP21 (Solyc10g082050)
MSTTKKRVILRNVTVKLGCSSSCIRPKFSSIFHPKPRRHHSADSAAVFNHHKTPNNKYSFSNSTI
TTATTFSPSPTPSPAHYSSDAERAVQGFGRIGGESVAVEKDSDDPYVDFRQSMLQMILEKEIYS
KDELRELLNCFLQLNSPYYHGIIVRAFTEIWHCVFSVNPGVTGAESPFL

>SlOFP22 (Solyc10g082060)
MNLSSLFKSKKKSSFSPFLCPLPHCGIPKTLSLRVENNDNIFNSQRLYNNVDDDMVDKMVEGLK
IEKDRFFFEAGEKTSSIMKVSSSILAKSNNELEILPIDESCVITPIDSMDIPCGEGAISIRDQVSSST
LSNNTNNSGKQVEYLPFNDSCIIKLSSSMDPYGSFKKSMVKMVEANLGIKDWNEFLEEMLAWY
LEVNEKNNHKYIIGAFCDLWISYSFTSSTTNIPNSFLFSSSEPKSVISPTSTSFVIS

>SlOFP23 (Solyc10g083070)
MKFSSLFKSNKKPSFSPMLCRLPRCGDLRTLSIRDENNHNIFNSQRFYNNVDDEMVDEVIESLK
LEKDRFFVEAGQKTSLILDMSSSRLSKKRTISKRLEFLPFNNDSCVITSMDSIDAYGETSRSILEG
SSSRLSKSTNNSTSSKRLGYLPSNDSMDSYGDQETSSILDMSSSSSNDNISSKGLGYLPSNES
MDATSILERSKSNSSHGFVYYVPCKKTYAIMRLISRDPYEDIKYFLEKMVDENLEIEDWEESLEE
LCGWLLEINEKNIHKYIVGAFCDLWMSYSCTSTINTPFGFSSSKPPSLYFMSLIENEADRMIAAS
TSSVTP

>SlOFP26 (Solyc10g083080)
MEFFSLFKSKKKPSFSPMLCRLPRCGNLRTLSIRDENNHNIFNSQRFCINVDDDIVDEVIEGLKF
EKKRFFFESGEKTSSILNVSSSKLSKSIGNKRLEFPPSDESCVITHIDSIDAYGETSTRSIFKGSSS
RLTKNDYSTSNNKFESLPLNNSCVISPSAMRVTSIDPYGYIKKYMEITVEENQGIKDWKESLKEI
CAWYLENNDNDKNIHKFIIGAFCDLWMSYSGTSTTNTPFGFSTSEPPSPYFMSLIEAKADQIIAT
STSSVIP

>SlOFP27 (Solyc10g083090)
MKFSSLFKSKKKPPFSPMLCRLSRCGNLRTLSIRDENNHNIFNSQRFYNNVDDEMVDEVIENLK
LEKDRFFVESGQKTSSLLDMSSSRLSKRRTISKRLEFLPFNNDSYVITLMDSIDAYGETSRSILE
GSSSRLSKSTNNSTSSKRLSYRPSNDSMDSYGDQETSSILDMSSLSSNDSISSNGLGYLPSNE
SMDATSILERSKSNSSHGFVYYVPCKKTYVIMRLISRDPYEDIKYFLERMVDENLEIEDWKESLE
ELCGWLLEINEKNIHKYIVGAFCDLWMSYSCTSTTNTPFEFNSSKPPSLYFMSMIEDEADQMIA
ASTFSVIS

>SlOFP28 (Solyc10g083100)
MKFFSLFKSKKKPSFSPMLCRLPRCGNLRTLSIRDENNHNIFNSQRFCINVDDDIVDEVIEGLKF
EKKRFYFEAGEKTSSILDVSCAITHIDSIDAYGKTSTRSILKGTKSRLSKNDNSTSNDMVESLPLN
DSCVITPSVMRVTSIDPYGYIKKHMEMMVEENQGIKDWKESLKEICALYLEINYIDKNIHRFIIGAF
CDLWMSYSGTSTTNTPFGFSTSEPPSPYFMSLMEA

>SlOFP29 (Solyc11g006670)
MGKKMNLGSWQWPSCTHSKTQSFRANHIFKTINSIFLDPSNTDHHHHGVVEIETTPESWFTNS
SESASFSTESEETGEPLMELIIKGVRSERLFFEPNCTSSSILEHQDQSQDQNQNQNQSQSQSR
DQDQSQSQSQEKLKEIEEDVDEELPFKESVALALESEDPYLDFKKSMEEMVDTHEIKDWESLQ
ELLQWYLKMNGKNNHGFIIGAFVDLLIGFTPSNCDSITCYSSAASSFSSIE
EKGE

>SlOFP30 (Solyc11g068780)
MSSKNKKIWNCITSNGTAGCGCSKPKLSEIIQPKPKPRPEPEPNAHSSSTSNSDSPSPTIMPAKI
VGSVAVVKDSDDPFGDFRRSMLQMIMEKEIYSYDDLNELLNCFLQLNSPSHHDIILQAFMEIWN
NGKNYIAN

Tomato TRMs amino acids sequences (allele name)

>SlTRM5 (Solyc07g008670)
MDGMDDRRMGCMGGFLQLFDRNHILAGKRLYSTKRLPHFTVPDDVSESDKFVASPAVSKELV
KPQPSLDQSKQAAVGVISVEPVVSVSVETPPKSPLPLPIFEVKEGTRSSWKFCKEAPRLSLDSR
AVVDAKGSLRPRELRTKGSVLSASRGENTEEGVVADGDDNQRPCPSVIARLMGLEPLPQSNN
ETLPKSELRRSASESRVSRDLFNGRYVDGNNVDFKELNNTRANVSNNAMKDNAVNERRSTMP
NARPTDRMGYPLKNENTERQKASNRSLSSSPWKSPQHRKFFFDTADIFPEPKQTVSLYGEIDK
RLKMRGIDEPSKDLETLKQILEALQLKGLLHNKRPSEQIGHRNFVYDPTLSSDESPIVLMRPSRS
VSPSHRRMANGTSPSSLRNRNGIGRRLNLSSESLPSVSPCRERPATERNARSPLRSKDSSSPT
QRENSVRLSSSVAKPKNLNVDSRRRAGEPAENRRVSPVQSPKLSSRRNSLEHNGAKRSPRNR
RETTESKQKEKITTFVTEDESSSISESTVSSSFQTDAERSNLEDYNEGRSLLERCDKLLNSIAEM
TAPESQPSPVSVLDSSFYRDDSPSPSPIMKRNIDFKDVSGESEEEIWLSAFSPVRSKCNDSMD
DTHLGYISDILRASCYLPEDSDVFLLLEKQQYLKGKDTSKVSRLQRKLIFDTITEILDRNRQLPPW
KAFSLSGSSIAKPSLEGIWSEFQRIQERESGNNLVEIICEVLKKDLAQDTVNGWGDCPVEMSEA
VLDMERQIFKDLIVETIQDLAVIGFKTTLLTASRRKLVF

>SlTRM19(Solyc09g005750)
MEKSRHRKSRSASGVMEGSKLAQKQVATPEVTLNSRSYCDEATRGDMIMHDFGKSSSKRVT
GTPIKNLLAEEMAREGESKKRPTSIVARLMGLEGMPSPQHIGRQQRRFSDSCQHRNEHIDSRR
RKQLFDEQSSKRSSMEHQEFKDVYEDLEASHVGNRRHSSRWNETGRFATPDMALIQQKFMD
AKRLSTDERFQNSKEFNDTLEALDSNKELLLKYLQEPDSLFVKHLQDLQVESASSKCSRIAVLK
PSNSVKYEGSAKSSKSVRGGSCKKSISLQKE
RLDGLLLQSQHRHSGHNSQKSSPVLSEGKEENILPTRIVVLKPNLGITQSNIASVPHHPDERKH
AKYLRASPGGAGEEEEKNSSKNMGIYRPKSNEARDIAKEITRRMRDSFGPFDGRDAYFRGSG
VKGYAGDESSCDIY
ESDSTGDSDIATLSCRKSSGRGNLKKSSSLGSESSVGREAKKRLSERWKMTQYYQDIEMAGK
SNTLGEMLSLPDGVTKHDYCDTMVHVEEATKEPGGRKGTTEWDFPLGISSRDGWKDVCINDS
SGYRSTSPPFFSKKHR
TRARREFSNKQCSVSKEPVNQEQSVNHHRSRSLDGMVNLRDEFSSKNSRSSKKKLHSRQLVS
DTSSKGKLRQRIDMNLKEDLSEKLSLASQVPSADGMSYTNASDDAETDSITLSSEYSVEMHRK
LPAECGSASPINQEV
SILQEALPEPSPTSSAAASVVLEYPAPEPESSISSKGADHRSPLSVLEYPAPEPESSVSSKEADH
PSPPSVLEVPFTEDVSSGSECFERVSAELNGLRMQLKLLKMESGPYADVILSDDEVESFEDNC
SLRSQSWQSSYI
LDVLTDSGLKTSDPDTFVTSFHTLECPLSPWVFDNLEKKYTDETTGPRYERRLLFDRINLGLLEI
VRKYVDPCPWVKPIEGIIWRWETYGMKNILHQLLRSHEDPANADTPGNVVEEMHWLAIKDEMD
VAVKDIEELLID
DLIEEVVGAV

>SlTRM17/20a(Solyc06g083660)
MLIFSFRTLLCREMTGGEMNGFQNGKNCNLDKPFPGCLGRMVNLFDLNSGVTGNKLLTDKPH
GSLSRSQS
DVVRMYPSGNQIEEKMIVSDLKRNSSNRKSNGTPMKMLIAQEMSKEIDSSQNPPSLVAKLMGL
DAFPTRK
SVSATQSHFGGHSRSHTDSSFSYCPHENGSLMEEMHQEFHQCPEENEYKDVYEVWQQPTKI
NCVRSKSPQ
KARHDETSIDKKVAFVRQKFIEAKCLSIDGNLRQSKEFQEALDVLSSNTDLFLKFLQEPNPMFSQ
QLQKL

KSVPPPPETKRITVLRPTKMVDNSRFGESGNKNEKEMKRATQVGQGNRVDESHCPVSPPAPG
WNDENPAQ
PTRIVVLKPSLTKTRNCMAASSPPSASPRVSEAEMKYVNIEDNEAQDSGEVALSQKMHENLGG
HRRDETL
FSSMSSNGYIGDESSFNKSENEYVAGNLSDSEVISPVSRHSWDYINRFVEPYSCSSLSRASYS
PESSVSR
EAKKRLSERWAMVSSNGSFPEQRHLRRRSSTLGEMLALSDTKHAGGMEQEISKEEPGTSYSN
LMNNSNCD
EGIDESPRNLLRSKSVPVSSSEFGTLLNADVPGHETGKPNLPEETTKPRSTKLSLKNLLFSRNR
KPSKDN
GRHLQSNNEVQSGVKSSYCPAKVDLGREFSSADLHKSPGKLVSQNSFGEQGIISPEVGLFVSK
SLPLENQ
CESQDEPSPISALDTTFEEDEHSACISFGRTKPDHGGELSVDPIRCNLIDKSPPIGSIARTLSWN
DSCID
TASSVPLRPFLSTWRTEEEEKEWFSFVQTLLTVAGLDEVQSDAFLLMWHSTESPLDPSLREKY
VDLHEKN
TLHEARRRQRRSTRKLVFDCVNAALMEIAGYGPDTCQRAIPHNGVSNNLPEGAKLILVDQVWT
RMKEWFS
SEVKCLSGDDDEDGNSLVVDGLVMKEVVGKGWLQHLRLEIDNVGTEIERELLAELVHESVIELT
GRA

>SlTRM3/4 (Solyc03g115000)
MAAKLLHSLTEDNQDLQKQIGCMTGILHIFDRQSMLASRRLIGNSPRRLTSGSSHIGSGTSEKE
YTSTYP
KSPAMESHTNKTVQDKQRLSTESSRPSFSSSSRSSSFSSLDCNKTSQQEPLAFDRLSFAETPS
REPAAGQ
PNASPQFGRQSLDIRDVVKDSMNREAQRFSAGPAVKEEVAESMSKPGDSPRPVQTLKNFDGA
YDSGPNGK
QNSSVDLKESLRVLAKLREAPWYSSEHRELTRSLSYHSKDTSTLSVSKDAPRFSYDGRETNHV
PFEQRDI
SKSTLKLKELPRLSLDSRVSPVRSLNSEPKSNFSSKSMQKDSGNTNAKSPTLQQTSGTPARPP
SVVAKLM
GLDTLPGSMSSTDNKMGLSTSSQVEAPVSFPRSSEVSDPCKPIRTSNTSKNLWKEPTSPKWR
NPDMAMKP
ISRFPIEPAPWKQPDRTRVYEKPISRTTKTPVKPAHPFPSVYSEIEKRWKDLEFTHSGKDLRALK
QILEA
MQAKGLLETEKEEQDSNFTGQKEHHQKFASPAQSAKLANQRMRQTDQVTAPTKRGINSSRNF
ESPIVIMK
PAKLVEKSDIPSSSMIPLHGGDSVSRKGNSVSRAAKEHQPRTSHGNSPVNPNEARRTSKPPQI
STRSQQL
PKEIISGSIKSSGSISPRLQQNKLELEKKSRPPTPPSDSNRSRRQSNKQHTEASSPGGRRRPRI
SNIQQH
DDHVSEISSESRNLSCHGNKISGQSNGNVVAESKVDFEVTSFERSLEMTSSPSSSIDASSYLRC
DLVEKK
SIRVLSEDEMLTEPAPEYPSPVSVLDNAVYMDESPSPVKHTPKVMKDESCNTADKFSSPPQCD
RSNTLAI
DATSSGLSSEINRKKLQNIENLVEKLRRLNSSHDEARTDYIASLCENTNPDHRYISEILLASGLLL
RDLG
SSLTSFQFHPSGHPINPELFLVLEQTKASTLLKEELCNDKMRQSNPKEKIRRKLIFDVVNESLAG
KLMLV

GPSYEPWLMSQKLAKSTLNAQRLLRDLCSEIEQLQAKPSKCNMEDEEDEWKNILLDDVVHRSE
SWTIFTG
EISSVVLDVERMIFKDLVDEIVRGDGSGLRAKPTRRRQLFAK

>SlTRM26a(Solyc02g082680)
MRSLLDLIDFDQGGMARKFLSQKRHGGVDTPRNSLELPVEASQWFYAGGDKAQCAYQMIDW
QEKNCYGYE
APMKKLISEEIARRPNTGYNAPSVVARLMGVDTLPLDTRPLPKHVEKKNEMKDGNPSKEEWLR
KVSIDHA
TQSSRQKISIPFNHDESCDSDRQIDSRKPNKYKPREHPQEEELQKFKKDFEAWQAARFKECSK
FVEHGTS
PSQWLAQQSLNKEKLTLYANSMRTAASEKPTELRGHTVAVNPWERGLLKHQKNINEFPAPAQ
NKTYCVKE
VIPNPDFQNHPLTNSYRRPDVAPAPTKIVILRPGPERIVTNENSWASSPGISEDRGSIEEFLEEV
KERLN
CELQGTNSKRSITVRGGGIETPYSERSPDAKQIAQSIAKHARESVTRDFGTTLSRSESTRSYKS
DIHSNG
ESSPEFVNRDTRKFLTERFRNVLKQETSHGVHRLARGSSRSMELNNETCSSEEMRYTSNTGD
KATNLDNM
KGELNMHNRSFRRDHGNDMLEQELSPRSLIRSLSAPVSATSFGKLLLEDRHMLTGAHIRRQHE
AIEKVTM
NVKKRQKEKFNLRRKVSSFSYSFILKGKLFGRKVHSWEEPHGQTYNLMKDFPSPPTGTPNFYE
RHENPTE
VPPSPASVCSSINEEYWRQTDYLTPSTTSDVPALDDSEMPRVFRDISSNLNELRRQLNQLDTY
DSEETMI
DEQAVEEEMLEIEDQAEAYIRELLIASGLYDGSRDKYISRWDPLGKPISNQVFEEVEESYKQLTK
DEEGY
IKDQLQKINHKLLCDMLNEALPSILGVPSTMSRFMKHAVGPMPRPPQGKKLLERAWEIVGVYVH
PPWDRA
FQSLDNIVARDLSSTPWSGLIDEDVNALGKDMECQIIGDLIQEMIKDMLS

>SlTRM17/20b(Solyc09g063080)
MPMKMLIAQEMSKEVGSGHNPPSVVAKLMGLDAFPQKSVPAIRNHFGGHSRCHTDSSFSYCQ
EENESLTE
ELQQELHQYPEQNEYKDVYEVWRHPPKMNSVRSESPQKARHDDQISFEKKSAFVRQKFIEAK
CLSIDEQL
RQSKEFQDALDVLSSNTDLFLKFLQEPNPMFTQHLSNLQSIPPPPETKRITVLRPSKMIDDCKFS
GSVKK
NEKDISRAIHIVQGNKAKSHMTFSPPIANWNIHENHAQPTRIVVLKPSLGKTHNFIDASSSPSASP
RVSQ
TETSFVHMEVDEAQESREVAKAITQHMRVNIGGHQRDETLLSSEFANGYIGDESSFNKSEKQY
AAGNVSD
SEVMSPASRHSWEYINRFGSPYSCSSLSRASYSHESSVSREAKKRLSERWAMVASNGSCQE
QRQMRRSNS
STLGEMLALSDIKTTRSIEQDNIKEDPQISNSNSPSNSKDDEGNHKSPKNLLRSMSVPVSSTAFS
SQLNV
GAPETVTGENDLSKHTTKSRSTKSSLKGKFSNLFFSRAKKPNKDRAKCLQSNDDLHSGPKPLR
SLSEIDK
YSGQFLDDPGAECSRTNLRESSCALTCEDLVEKQTTISPEVVFSGSRSVCARYLCENQDQPSP
ISVLETP

FEEDDHLACISSGGIKPDRHGAELSVHSLRSNLIDKSPPIGSIARTLSWDDTCADTASSVCVRPS
SSTQR
TEEVEREWFSFVQTLLTVAGLDEVQPDAFSTMWQWHSPESPLDPSLREKYIDLNEKETLHESK
RRQRRST
QKLVFDCVNAALLEIAEYGADNFQKAIPYMGVHNNLPQGTTRLVLLEQVWDWMKEWFSSEMK
YLSTDGGD
LNSLVVEEMVGKEVMGKMWLGNLRIELDNVGVEIEEKLLEELVNESVVELTGKM

>SlTRM13/14/15/33a(Solyc01g094640)
MAKRSHRHALRYEKDRAGCIWGLISIFDFRHGRATRKLLSDRARGSKPVLGSASSSSMQEIPN
PSDDRLN
IEDDEESEVAVPDPRTSVKELMEEEMVNEQSLKDQCNGSEIDTEDVDSQKSWRSRKNSRRTR
RAFSRPSN
TLSHDLDDAGNLRSEAPCHQDSGGTALDDLDIVMEELRQIHQKNRKFVKLRQGSHNAHNNQS
DQTHPVVE
EKVNAAIEVFINQRSRNNKQLGEDNKTLQSKEFMDALQTLSSNKDLIMRLLQDPNSRLVKQIGS
LEDAQF
EEKQRPNLISESNMSEENRVHAKTDDVINHKQRKFFRRRSKSQEVYPPMGNETPRSSSKIVILK
PGPTGL
QSPSAQINVNTPARSRYTEKHTIQNERNTSQFSFTEIKRKLKHAMGKDRHGISPEGTIRRFPSE
QLKRCN
SDRGVFGENLGWSSPNRDHFYTEKFAKSPLGMKSGDKIVKSKGVEAVTLTGTSDVPRPEMSNI
YIEAKKH
LVEMLDNEDETTEASSGHLSKSLGRILSFPEYNSSPGCSPRNNSKDGMLPFQVRKPLTDSIQV
ETDDRLQ
HVREDHVTGPSPSSQDLEIESSCSDKYPNESTKSASTNLDVPCENGNTMDEIAASTGHTSPEG
DLTEEAI
KTRCQVEGEILSVPIDREIQIDGDATNAVDDGNSPHVFEVSFDCLKEHPSGKDQNSLSSSPASP
AESSSL
VKVEDPDSAVDRKERPSPISVLEPLFLEDDVSPASTICRPVQLHTVDPEIQPRKIHFEEPVSSISE
QDCP
IVCFENEESAFEYVEAVLLGSGLSWDEFLLRWLSSDQILDPSLFDEVELFSSRSCHDQKLLFDC
ANEVLK
AVCERYFGCNPRVSLGKHNIRPVPKGMDLINEVWEGVEWYLLQYSAPHSLEQLVKKDMERSG
TWMNLRLD
LGHIGVEMGEIILEELMDDTILSISGDTLECAEDVLFPVTSETESSVDQ

>SlTRM30/34a(Solyc07g032710)
MPPDSLRSAVYRSFITCDDPKGVVECSTIRKSHMEKNTPCSSHKDEGRQTVNHTSSFHLMEVS
REAQKLN
QVIDSWSKGMTIERHSNDIAKDLLKGALELQESLVMLGKLQHIAKLKKKYKHELDGIPIQRTKSE
RISEH
RLNRFEFQKPRFSVDGDCFDELREVIRDNFARQPNSALQFQTNSEKASVGTRIKSSHVPSTSS
SHSSIVQ
SQQVSPPLDGPNLIARLMGLEEIPSKSQHQTTHKVVKQMRPIFEIDLPKAKKPTFISHKVDPKRK
TFDEI
IETMYFKGLLRSKSTHKFVDSPPIVIMKPLYEQNPSDSRNKCEEISPDDHKGASIYRKTQAGKDH
NNRFS
KERGEAPSKSKTLQVLIQPNTKIIASSPGKHRGEANAKSKTLDFVSQEKQHNKNIRASSPGKDL
GEAPAN

SKTLKLLLQEKYPNAMIKASSHRKYLGDATVKVFIQEKQPNTRTRASSPEKTPQTKKEPIGKRE
DGTQRV
APAIRNSKEMKNAKIDDSAKFQDQSKMSTLKVRKPERKPLAAQAKSTIYDLKRITTTASHNSIKR
KKNVK
ANKPIKSTPIATVADIKHKDESKEMVQAEDKDTDRAITNVTSSEELQLEKRANIFEDLVTDNAVN
GENVP
CESSVLSTYCLGDIKLVEQINCNINLDFTENVNFNSGATTRYLLLSSESFLCQSKELFETDVWEP
TVWQT
TSVDHEIADSTLLLDCANELLENKRSQCALAVNPLSMKAIKMRKFYVSFEKLVKEICDGIEVLRS
YNKVA
GKNLSADALYPLLERDLWCKGVAGSAWDLAWRTGLTKNEVEQVLNDIEKYLLAAFIDDLLTDF
ML

>SlTRM25(Solyc03g006840)
MGREWLYWGSGSGKSSSRRKTKGEEQVMNNIINDEAAAPSPAGCMCFQIFDLPHFQVALNQ
QSRSLKQHH
HHPSFFQHHKDPSLLKGVEAPRNSLELDEAVPERKSVSSSLSSSSTMKVDEQNLNIPVGIQIRT
SCDSRS
PRVSTSGSRTRTDYGISSECSSSSPAGTKTPTLVARLMGLDLLPENNNSPRISISTHCNTKSQS
KNVLVN
NNSSKNRRRFSSFESSDIATGTLSLPETPRISLARRSDVDHHHHRHQHHRLSLQINKENMGDA
FEFSDSS
STAKMGRNSRRSFHQQENDNQTRSPGYYARQIVKQVKESVSSRKVGHDITNTSSSIRRKDNQ
LAATYDQV
VLLKPKKPSNGNDDDFPSSKQTTPSCSPRFRFLEPKSTSAKHQTSHSPKFSPLSPLSETTTLSL
PAKIVT
KPKPQSSPKVQVQQRKCDKFVPKKPPQACDAIRSKKEELFVRSAAATNKANFSEKKCKLKTPL
SNQLVNT
STVPTILPLKKDPSPPTTKLLIKQSQESDTYPSKRRSSSSSRELSSSSSHNSYYYKLTTLQENRD
KCNGA
ISIDGLNFHHQYIQRILKRTGLDKSSPISLAKWYSPSHPLDPSIFHYLELFNSTTHNSTLRSNRKLI
FHL
VDELLVDILIKNNNLKHRSMNGEGLIDALCSKIQDFPSANCQVLEDIDALIERDMKIGGSVFFEEE
VESI
VCEIEREIMEEVLHDDGAV

>SlTRM13/14/15/33b(Solyc08g081160)
MEKKRSSRNPHQIDKSQLNCMWGLISSLYFGQNQRKQKLLSNGKGSTKNVIGKNSRKIDALTY
CSDQLYG
YEDGAEVEALGVRTGDKRIKTSIHEEISGEMQKSTQIIARNEQHEEVDYGLFDHMISKYKPSPKK
GCKNQ
SPVYDWKDTETGDIQQASSSAEMSIHKLKLASILEAIGSQIHREDGDSKRSSIKNDQLDEISLQVL
QTSA
KAFIDQMFIDRKYISKGNMSYEPEQFSNALEMLNSNGDLFLKLLQDPNSLLAKQIRNMQNVQMA
RDSIKS
FMSNRLPDCNISKSEHKHHQSAFEESSNSRPSNKIVVLKPIPRTVRCSENVYCYCSSIQSHHST
SSKGGN
LQHKNFSLKDIKRKLKYAMGEKWKEKHLISVGSTVHKLHSVSDRKNLEVDEGGSSCLTTARST
NSFTESN
NKNEAQNKQISTSEAPKVSFLTEKVRKKLDASAISYTKKRELDISMEAKRHLSQRLNFVNTTDEA
AMSTQ

PSRTLERILSSPEHDRLFNYCSKQDRKSNPEQPCYNDTSIAELPRDPTHTSFQSPQRHKDSQH
LKSSMLA
SPSEVWSPGSSTDVSSTSPYSMYKLRGVDSIMDRGDHPSPVSVLEPVFTDDLISPSRNEPSGT
VLQPRRI
NFEGCLNKESTENAILNRAEPDTLRTYIQSSLHTLHLNWEELWLNRHLSEQMLDAMLSDELETL
ALQCHS
EPKLLMDYTNEALLEAYDSHFRYPPWLSFFQPKLWSFPPEKHLLEKVMNEVKQHLVPLMDQPT
LNDHVET
DLAKSGSWLDIRDDTEDVLTQITDDVLEESIMCTVLQLQNLFLV

>SlTRM30/34b (Solyc12g007140)
MLGKLQEASEYVTGLRKRESDATGVGKTKSERLVADHRYNNNKDEFGKSSLSVDRSRDCYDE
LREVIRDS
LARQNLLPPCCASEKARSGRRKIDLYQDFPSTSFTSLSETSMEKACVADARKLVMSPELPSTSS
SQFASF
DCSRDKEKPKVPNLIARLMGLEEIPSTPLHQKQLEKDMIFKPTRPIFEIDLPKAKKLSVINQKADP
KRRT
LDGIIETMQFKGLLRCKSNNVISHQLKSSAADAPPIVIMRPVYAPEVQAERFSTSIRDENPLDTKN
SFGK
RNLKEESAPVNFTVHRKMHTRNIQKSSCIPEKGSKDHNEETLSLTKNRASSPGRTKQPKKEVID
KRVERT
QRAPGAKRSGEMKNVSPNNTTKVQDQSKRTTAKVTKPEKKSNVPEKLVASQRSADSKRITAV
VSQNSRNR
KKNVKTDKSVKSSSIVPVVENMEHNCEQDSDITVTNLTSSEEPPCEEVAEISKSVVTDNLKNGE
CSATES
TMTLIQCDHNIPLMEHTRYQIRQDSTEKEFLKSRATTRHILLSNESFLSRAEELFDTDAWEPTVW
KTVSV
ENEMPNSTLVLDCANELLENKRSQSALTISKSPVNMSRVSISFDKLLNEICDAIEVLRSHTKVDA
NILSV
DTLYALHERDLSCNGVISTTWDLGWRNAFTLDEVEQIVTDIEKHVVNGIIDDALTELTL

>SlTRM6/7/8b (Solyc01g100290)
MEVEKRTSKGGFLQLFDWNIKSRKKLFSNKSELPDNSKQGKENANGSANLRLQQAHDHSLGS
NSKQNYDF
YSASSVAEDESYGQKAPGVVARLMGLDSLPTSKESDPNFNASSDCHSFRDSPYLSFIADFQNE
HHMIVDG
NMRNKLDGFKRNPVEVRLQKVQSRPIERFQSEVLPPKSAKPIAVTQPRLLSPIKSPGFIPPKNAA
YIIEA
AAKIYQQSPRPAAREKVQSSGSSSAPLRIRDLRDQIEAVQRQSSIYEAPHRPKEQNSVKNVRR
QPCERGQ
VQSDNLRQLRVSEVSRRDVSQNKGKEKSVSLAVQAKTNVQKREGKESTSSKNPLNQKEQNES
KSGRRRTS
VKVGERKNSLNRPSDVLRQNNQKQNSASNKDGESSNTSAPYHKEKKSSSTGNMSRSTKTVS
RIVVNTTAA
TGIASIVETDVGKDLSSSRDSRVRSFTGKKQPVNVDIGSDECGADNMMKNKDERSIKCNLTIEG
CSNWET
ADRKNGSDVVSFTFTSPIKKSMPGPTSSSHVLEKNSALCLFPGSYDDQSDSRTSTMPSFRIGG
DDLGILL
EQKIKELTSKVGPSCEDFIKTGTASTSTNAFEDSVSIVAHGRRPQVDLLNEKAGDPGHSSVDDL
QLTATQ

MWQGPNRVENPKTASSITCEGEFSLASSMEPSISGGSCSSLDSFRSLATDGSKYHLSDGSHY
MMNWKTYM
RTHLVEGDAELLDSASSASLADAGEKESTTTLTSSNFNESAYWEFQYIRDIIRSSDMVMEEFLL
GEVQSI
IALDLFDKLENQQARTNKNAEEQLKMRRRVLFHSAVECLELRCKLSFGRGVEAWAKWTTLVQR
KEWLAEE
VYRVIASWTSMEELMVDEVVDKDMSTQDGKWTDFSFEACEEGVDIEKEILSSLMDDLIGDLM

>SlTRM26b (Solyc03g032110)
MTNDWPEKKNCYVKEVPMKKLISEELAKRPNTGQNVPSVVARLMGLDTLSVDEESLREVSSR
QTVFDSFD
RRSRNSLKFNELKPREHPQEEELQKFKKEFEAYQAAKFKEGSKFVELNTNTVLYANSTRKMVT
ERFIDLK
GLAATENIHERGISKIQKDKNEFLAAARNKTIRALNVKSGSAPAKIVILRPVSDRTGKNEESWAN
SPRIS
EDGSSMEEFLQEVKGRLKFELQEKSFRKTIEKPSDAKIIAQCIAKQARESVTRDVGTTHHRSKS
MQSDRS
EIQRDEASSPEFTKRDTRRILTERLRNVLSDESSHDIDKHDRVSSTSVAQHREKSKSEEMRYAP
NEVCHG
DDMKDESDRQCRYSRQELSNDVMLDQKLFHRNLVRSLSAPVSRSSFGKLLLEDQDMLTGAHI
RRQHEAIE
EVTVNVKKWRKEKFNLKAKVSSFKHSFVLKGKLFGRKIQSLEESHGNKQMHMKDLQNTQTVA
SKFYVRQE
NSTEVPPSPASVCSTSNEEFWRQTDNFSPSSSSISDVNPLDDTEIPHVFKEISSNLNGTSKNLC

>SlTRM18 (Solyc05g054770)
MEGKQHTPSVIARLMSLDELPPRQHLPVKRRRVLSQNYLQKMASIGLREKSSFSVGLSRGIST
QKHQIVK
DVSAAKLKMRKYTNSSVTPIKEKHSYLMDFEGTSESLLSTKHLRDLQAYKPYCHPVHSAVAKSI
CKMSAK
ATAKEKNLRSLHTLEIGSPKDDIGECSIHHLKKINFQLDPNENMPHPSTRVIVVKPSSGKYRKTN
HQSVS
LRHGLQSVPVDLKYKKFAEHENGAVHNERPGRAESISDTFLKAEALKLPSSKLFSTQRKDNTLN
FFSKRS
SFSKEVKKQTIEKWKLMNGLQEVETTCRSQNLGEMLATDDLETRPQFLDSKRDSQRFCSSSS
VNTQNSGS
CSKDSLVPNHSAGSRIASGSSEGMIGHKASLYGWCPRQKVAGAEKHSKSMNQKQKDNMEYR
DLNLKETDQ
RSPNSVLEPPFQEEEPYTSAFHGLCSVARQLQFLETNSEETYSEGSEMGVSTDGDSETGSPD
LLQDSENI
LKDFKTADGRDFSYLVDVLDEASLHGINLGMCFETWHSLEYPVNPSVFDLLEKKYGKQTSWLK
SERKLLF
DHINSGLSEILHSFLEIYIMGKSFKRRCCSTMRRTDIEEELWRMLVSHENEIRKDLSGKAIGNETK
WLQV
EEEIGSICREIEKYLLEELAAELASH

>SlTRM6/7/8a (Solyc01g060410)
MVVEKQGSKSGGYVGGFLQLFDWNAKSRKKLFSSKSDIPELSKQKKRCDGNLPMTRVHLNNE
DDTTAVSS
IKGSSDYSCASSVTDEEYYGIKPAGVVARLMGLDCLPSSTLSEPYSTPFFDSQSLRSAPSLSRN
LEYQQN

FQTVYSSNLHEKIEDLGRSSFEPKQQKIISRPIEKFQTEILPPKSAKSIPATHYKALSPIKRANSIPP
QN
AAHIMETAARILDAGPQATSKVKSPLIRSSSVPLKYKDLIGRAEASQKVAKIAEASRRPAESNAS
KYLKG
QPMNKSWSGSADIARQKDFSDSDDSFGGGKTKGKSVSLALQAKVNVQKREGLNAGSSRSILV
QKESPSKG
ISNQLFTSQPSTEKNTHKKSSVHNSSSVLRQNNQKQNSIADRGKSPSKQFLSNSQGKRTLSGD
SSFARQR
SSGKMAENSKVSSRRLSREADNKKEEAYSCTKSVSRKKRPSDGDIQYEKNQATGSMSTHKSG
KLIQSGTF
MDREISWGENSKGKGTDIISFSFTTPLARSVPTAEPPREVLGKSNEFSTDFRSNNMQLTSDCM
NNLKAPL
GHHNLSGGDALSTLLDQKLRELSSVVESSRQKTSNSSSSIFEDLSPSLNGLSKTTMLHVNRNH
DDMEVDD
LVSPCNPGFSSTVPLGITGQHKHQVVEEELSGYGSSEYECRKLFGSRFLSPISVLEQSFLTESC
NSSDTA
ESNNTGACKQSSSVQAKEVFGICSWNKFQSMEPEVDLLDSASSTFGKEEERKSPNWELEYVK
EIVYNIES
MFMDFTMGRCQKIINPHLFDQLERINIHRHDELKQRRKVVFDCVGECLDLRCKQFVEGGYDSW
SKGVLVV
KNKKRLAEEVYREISGWSGMGNYMVDELVDKDMSSGFGRWMNFEVEAFELGIQIEKRLLNSLI
DEVVADI
LLL

>SlTRM9 (Solyc01g091830.2.1)
MASSSPSPLARLSLERRPKLLKEFLLQDDPYSSNDFGSYPNKYIHGSTIIRSNKGSSHQLLRSRS
SRAAT
ATISAINKVISIVKFLPFTSVKSPSIFPRNISRKLSRKNNYKKSQKHNVDQDVSVKVKVKDILRWK
SFRD
LAEEKSTPLDSSYSPYRYGTITAMTTTTITTGKRTSWCDSDSTAEDLPSWWGENGELLGRKNS
VGGYCME
TTKSIINKEELCFDENEQHSPVSILESPFQEDDDEGSMAFSFQKRKSMLLHRIQQFESLAEENIN
SKVEE
ELKEDEEIEEKAKQLLINCEVNYMDDDQLLFDFFWNELITSGKKHQNNVSNVDEKLLREAKSWI
NDDYNE
EFEWEIEDKREAYIKDMEKVANWNKFEEEKQEFILDLEFEVFNDLVNEVLVDVFSHN

>SlTRM22 (Solyc02g086130)
MNDSLAITASSLAITEKKPQRPGGCVGIFFQLFDWNRRFAKKKLFPKKLLSPARLKQASKKFGG
DEKQPK
HRLIANENSGGFPIAKSNGMSNTRCESKREMKAPSLVARLMGLESMPAGPGSKAKKASASET
GSYVAEKL
DARPGGSDKEDMDCEKAEIKRELRPQKLQKIGVSERRPVSRFSAEALQLRTVLSRPRKHQPKL
TSPVKSP
RNVSGRNASRLIGAATRILEPGLQKSRAKCALTYPKYFSPLEDKADLALHHLEVPNPCVDSKTS
EVRASV
PSCKNCGYMLHSKNGTPNGEEHPSSVSSPVSSYSQPSCQGPGRNMLRLPIINSRDQLERVFE
GSSSDANA
EIDDVSYCAELILGKRPISRSRIAMHGACQGSNVKKDASSVTHVLNQKQNQTSQNRERGFMKS
KQSSLQS

NRVLAAAESTINTKSFVAQNRRLGASTRLRMPATADGCKFETERKPYSRRSDSLSPVRKKRLM
NVSRQGE
SSSFVNANLGRESSPYSDKTSRKDVFPISSVNSHSTKPKLPCLRESGATNNSSEGSNVVSFTF
RSAMKQK
AGIHAEVTKRKSQNSSSFDATPGRSFFTGNDETACLQKSFPLKGDILGALLEQKLKELTSEEEF
AEGDAA
PRKSTATILQELITALNDETQFHLDSLPSKPNRKEDLYDDREVSSRNTSMNFQAIPDSATDLVGN
SLDND
HLSPGCVLEATFSTDSYLSSSPNSSSKDKVLAESVDSIYDEPLFPEPDRDLSDCATSLFTRRSC
RALITD
HVNNISGVLSKINQLKGSKLGYANEVILNTELILGTSPEQQALPVDDGLSVSHFLLNELEMLSSLL
WMTF
GQLLGCNDPKQMNQLKGFAFDCLLEYLDSKFGRYSDSGFRIWSKLPSSMTKEILIADIIEEVKE
WTEFVG
LIPDELIEWDMSHSLGKWTDFEIEEFECGTEVDRHILQVLVDEVVLDLYSSS

>SlTRM1/2/3/4/5 (Solyc02g089050)
MSARMLSSITEDNKDLHKKIGCMNGLFQLFDRHHFLIGKHLHGQNHKRLLTGVTDKMETKCTM
QLATEKT
PRDVARNKVESSKANPKVEQSKKPQEEQPLCGQRNLPESPSKTLSYKQPSSPSHSGRQSPDF
RDVVKDSM
HREARSLSVKTVTKVEGKLHVMKHIDSPRPFQQPNCGKPSDGTRNVTAKFRDAPRNSKDDLK
HAPRDHPR
FSYDERDSREAMRSSIRLKDLPRLSLDSREQSFRSSASESRSNFLLGDHKRSSSVVAKLMGLE
ALPNSIP
SNEVDTVIPKSFSTNNSVSVSIKTAEKSKNNQVTRFSQINEKDFGSPRMKSTNSIMRAASTSRL
PLEPAP
WRQPEACRTPLKSSARNTDVELSIQSPKLSSSVYGEMEKRITELEFRKSGKDLRALKQILEAMQ
KTRARL
DVQTEELADSDANLEIVQKRQQCNLLSPTIKGTRPPKREDTADKKTWKDVTPRAKNIRDSGWL
LPSPDRK
TKEGTSRAVQNPTLRQQKEGSYPAIGRSSGTASPRPLQKKKQSCPTTTSPEFSRVRRQSIKQS
KESGSSK
RRLQAKPNNLLRVDEESSEISSSTRNFSEQSDAASLQSESNNSLSSHAEGEVTSRNHCVRVNA
KRLEDSK
DKSDILRLNEDRTMAELAISTIEQPSPVSVLDATFYEEDSPSPVKKKTTAFRVEDATDELWYLEY
QDHSP
YSKRIDLGTEATTQKKLEHIKDLVNQLRLLDSSYGASTDQFGSLSQNHNPDHRYITKILLASGLL
KDVDS
VSMAIQLHSSGHLIDQKLFHILEQTEERVIPATEHSKTSARIEFNQKMHRKNVFDTVDEILACKLA
SESC
LMQGQDHLSAQQLQKEVQSDIDLLNAKKVGMDSEEDDLISILNADLRHQSEDWTNGDSEIPSLI
LDVERL
IYKDLITEIISDEAREQQIRTKRHCRQLFTN

>SlTRM30 (Solyc04g080270)
MFSPKKTLLHSKTEGKIMSGKSDFAQKLLHDLKLRKERVAAAQSSGYSYSTTRDARANSGQTY
RGSRQTK
TLESVNSKVGSTSNRSFRFEDSSGQIVTYGTGRVRTSEKVGDLSMALAFAIENGGKFTKMDSG
SSRNPVL

TFLHQFSQRSMDISKTDRRTYHIPNSQFPSVSQVHINEVSKGIQKLNQILKACSNGLNFDRNSIE
VGQDL
LKGAMDLEESLRMLVNLQEASDHMIKPQNKNRITLLDEDEDEDAKIVDQKQLDLPRFSFDKPSK
NSYVAK
GIGKNDIKQRLMALSYPDQTPKLHEKQPLSRNKSMSHKRSASCAPDFRNLDQKNQLKGTKSG
SEKGRISN
VIAKLMGLDELPQKQDNKASRKGSDPKKKQEPVLMRSAGFIGTRDAENRSSLNVDKNMISDILP
VQDAKF
MRKAEITRASPSRNNDMDSSGRVQQQEERSIAVNKDTGTVPSGLPMINNMMDKPHSKNIQLN
QFNFQQKQ
KEQNQTSVKGKIIKTVEIKETISQTKKLQTSRVPPIDIVLQEDIIQKETDKFPLSNEKKALAKDEVH
HMQ
KLKKSEGQDEKHQAGKKEKLPSNKTMQARTHKANQVETISSPKSRSSAASLKKKQSSRNQSIL
GTKNPTK
SKNGAPAKDSSNGINQALAKHRNSSTFVGMQSSENKNTDQNVLSKEVLSRSEKLNNISQSSKQ
EKPVNLP
STDRMDHHNKIHRTETSPKIDELSPTLQDTELQKDDKSCSGGAEQSTESQTREANADNIGSNE
PDVSMEI
LDFQTELLGKIENSTSCNTIMEKECDNLTDSGTVISNENHQEKTPQEVEISMDQKVREDRPKILQ
ATDQF
NGIHQEASQNSKLFYDEQNKSFPAKFTGKGGIKISNVVRNDQETALTLVVQEPLTVPEKHFKET
VIKNQL
FLNTAEALFKLNIPISILHASDQNNQGEDVKLMIDCGYEIMKRKAIRQELAVHPYATISIGYTKTRS
LDN
LIKQLCNDFDTLKSYGGNEHMSDERDEADYLHNMIGKDMSNRNPDVNSMWDSGWSTEQIMF
SFLEKDDVV
KDVEKHLLNGLINEITMDLLRIAISV

>SlTRM16/32a (Solyc06g076130.2.1)
MGKNKWSRHVDHEDEDIHPGCMMGFIHALGYNSWHSRVKRKSLPRINDGSSHIRSIYSSKGKL
VGQDPSELEMLLDDNESHFLVDKGKRKSKGSNKKSLKAKIKDLIAEEMHKEKKKSKKKKSGSS
DQPKFQTSGSVEHSDKKHPSVLVPANVEGGESPDKKRMNAIDAYDTSNDKNALNSQLKDHAE
LLLEILRESEAEYQNLSRGQLASYKKARLTKSGSYPVSRKTNFKPIKLEDKKKEVWSSAKGERLI
GVTRTRSLSNYAKTLFTSLGLLDDDGRNLKLDKSSSLSTKGAVDNKENNEELVDVEDVNKVDT
DHQNKSNGCCEIDGFDISENTIIHKRSSSLNEFMNRYTKLFEHSFKKEMNLYPSKSLKLTSEYEI
PPSMSFRRIRSLSYSESCSLLPNGVSGDIRFSEWLVKTVEERNSHMRGEIEKEEKSIYSTEETG
CKIEQIEGSEDINIDEADNEVVEELSEIIDEVKVFNGNSYDEQEIKCNESIPSDVVPNSEICFKEDI
SNYAEFQISEGFAHKQNVDVDILAKADHHDKSYRKENADFFYVRDVLDHSDFSRNFFKTTWYS
EAQVLNPSLVKELESLWHEEEEECCLGEFDFCCHHHLLFDLVNEVLLQMYDRSFTYYPKALTY
NCRVPQLLENRMNEEVCNNVGTLLRLKPEQESIDTIVDQDLKMDDGWMNLQLESACLALELED
MIFNDLLEELRCF

>SlTRM10/11 (Solyc08g080280.2.1)
MDSKLMKPLPKTLMLKDYLLDDLSSCSSSGFRSYPRRQCCTTVRFLLEIDLKNKYQPALPPPPY
KNKPILRSKQSPPSAKVSAFHKASVAVINAVKHLPFAGARSSSTLKKKKPMMRTIFPRSISRKLK
RSFWKRGDHKEIYWWTAFNRLDKEELKSPVLSPVVIGKITGDSNSSTTTVSKSKCNSNTWSSD
SDYTASTDNSLQTSSGNSEVNSSETVNDAVASKKFGTENVTCSKKVGATTGDDSSDSTISSHG
STTNSPNTKKPWPNEEKEQFSPVSTLDCPFDDEDEVSSPFQHRLSRVEGTTTKKLMKKIKRFE
CLTELEPLNLDKRIASSESESESPLNNSSETEEDKQTVEDMVQELKASMPSYSLKFTTEKLLFD
FFKERILNGDDELKNKLLESALEWINGKPIDVLLDWKVQENRMAYIRAIENRGEWKNTELEKQQ
VILELEVEIFGSLMNEVLVDVMLS

>SlTRM31 (Solyc09g005570.2.1)
MAKNPKDTSSTKCFSSIFQRLICGGSLPTHPCDQFKEANTTTYEANSNKLVGGANSSPGIVARL
MGLESLPREEKSKFGSFSRSKSANSLDYLMQFDLTQQFHHRRVRTSLSFREIPNQEESKSEYL
VFCINDEKQEMMKPKKKRQNVEKKQVSGKQNRIGNNKVKKQVIKFEDYPTKMCVESKKNKRK
SKCVVSTKIQPLYYNSTPNDHLQQDATIPSEGKIQVNSDAMKSKVEKQKEISKEKDHYIKVVGEI
CRLTEEELNESHWITSIRNGENINFEDLCQQFGQQLLQLLIDQLVHELVIFAQ

>SlTRM27/28a (Solyc09g009220.2.1)
MAQKHLHELLQEDQEPFKLKHYIADRRCQLNKNPSKYSLQTVKKQKSISSPSNTSTLLCKHACF
FTSIRKKSDGGSSERLSPINFPASTKSPVRKVFLHIPASTSSLLLESAMRIQKKQKNRPKLKKTQ
MGFGLFGSILKKFMNKKREIGMKRNEIVAELTSGSCSCNHSRLSSAGWSESNEEKSMDFETSS
SCRSENEEIEDIELGICENKYCSSPISPFRFSLQKCPSSTGCRTPDFSSPVASPVRHKTEDKENY
EDLSNIEQEEDEKEQCSPVSVLDPPFDDDGHEREYEYEDEDEDEEYDDCGIDCNYALVQRAQ
QQLLSKLRRFEKLAELDPIELEKLMLEEEEEEGNNDLEYDNDSLLSYRDRDFETFASEVTFPFD
MKRLVSDLIYEEKTETNNSNNREEEVVFGRVCKRLDSWQHVRSDTIDMMIESDFKTELDDWKK
FHEQREEAALEVEVSIFRLLVEELAEEIVHFGGHHW

>SlTRM16/32b (Solyc09g010790.1.1)
MGKHQDPSISPESPPGCMWILHRKRLPRKRKGGGKRVAVVEDLEDNATATESSPLVATLCTY
MRAKLTMSSTVSFQIVSKIKEPNQVSKSSMLSRIRSLITQEETSKKRKGRHRRSSSCPIQLERTN
SIQHLDLADLQSSHDKTLEHTNYKEMYSVASLLDPPTKKIRDRAVQEPMEVENMRNDSSKLML
GFIKSISFPSRRSRGRKAVRSRKCRHHAKGEDDQSQVGSEVSGSGGFNSNSVTSTDLSSDDD
VGKNVLIRRAESFDNASACSPRAEKKDRESSKLILNRFKNLKQKIRYALEESRKERHRIFMDAVL
HKVPHGHRSSKDIEKGSTFAHCNSPFMRSEKMTSFRRTSSVNESLHKYNGFLDSSIYREEKHH
ISDRSSFRTSRSPSPGRRSPIGLDRILSMPDLTYYNSFKCEDSPERGSSYTLDRATSSSNLYVGI
CKSNEQKSLDIPLGSENHTEKGYNSDSKSTIFLDVSERFDDFGGLKTRENSSPVENIIEGTSSVV
SNVDKPIPVPLPDMIIQNATSGANELSAAKGAEEDVVHTDKRGLPSSDLNRILQIQVDKRYECEF
NYVKDVLELSGFSGDKIIGKWHSADKPVNPSLFDEVEGYCLLDQEGVTCDQLLLFDLINEVLLQI
YERSCSYWPKSLTCHTHIHTMPIGYHVLGEVWKDVTLCFESEMKNDQPIDDVVSRDLAGGET
WMNLQFDAVCGGLELEDLILNDLLEELVFT

>SlTRM27/28b (Solyc10g083530.1.1)
MALKHLHELLEEDQEPFYIANKRFHDQKPPSKLCKNTCFFSFGESPELRKSPLCLSSPAPAKSP
EIEKFVLNVPATTAALLLEAAIRIQKQQSSSKSKTQIKKVKFGGFGSILKKLKNRNSDKNGEKSFT
CSCINSRVNTEISEEKLMDLENYGDFGSSPLSPFRFSLQRCPSSSGELLPGFTSPAASPVYHTK
EDKENYETILGLASIEQLEEEDKEQCSPVSVLDPPFEEEDERENEDEDEDEDENLDCNYALVQR
AQQQLFCKLRRFEKLAELDPIELEKILLEEEEEDRAEEEEYQVSNLIFEEKIEVKSLNYGEAVFGR
ECKKIDLSSQELRSNNIDMMVKSNLKNEFDDWNEFKDQREEIAIEFAFSIFGLLVHELGEELIHLA
HS

>SlTRM16/32c (Solyc10g084750.1.1)
MGKGLRSRRECNPPGCMFGILHHLNQQHRWHQVRKRLPYIKQGGAKHIVAAGDRGSNATAT
DSSNTPEKINVKSEDFPIVVKAEAKTVATQQKSSIKSRLKTLITKELLSSKRVQHRRTLSCPITMP
LEQTAPIRYLGPANVEHSPKIRLNDEILQQPQNKNLSVASLLDPPLPEKRKDAVTNNKKCELCAS
MLDMNHLKQCDTKKNGKQPSTNFSFRRTQSLYLREQTKNVSVEESKLFLDALDLLNMREELFL
KILQDPNSSLARQLQGTRASKGLTKSVSFPSRLSLEKIAARSSNDKSSQDKSQIRGKLLGSAGF
ESAEKLNRHLVARNKEEVTKGVLTRLESFDKLPPSSPAALKHKRHNSKLVLARFRNLKEKITHAL
KESRKEKHRIAMDAVLHKVPHGHMSLKNVKPDGSDGSFSESTGNTSRCHSPFSKSQMKSFKR
TSSLNDSLDSYSRLLETCFSRDEKQNSSERSSLRASRSPSPARSRTIALERILSLPDLRHYSSFR
IEETPEASYSETLDTAASTASSSNLYSGATRSNEQKSLDIPLGSEKKTQQDSCSDSKILENSLDV

SENSDDIGGLKAEENSFPVEYNMDDNLSTNSTLDKPISTTLPDMIIQEASTIPADLSAIEGIAENAF
DSNEEEILDHEQMTSLLQIQVDEKNKAEFNYVKDVLNLSGFSGNEFMNLSVFEELGGSFLHQP
ECSGYAEERGNYDQLLLFDLINEVLLQLYERSSLYWPKALTSRSYIHPMLHVGYYLLEEVWKDV
SWWLSYKLENDQSLLDDAASRDLDKRDNWMNLQFDAECVGLELEELIFDDLLDELIFIDVY
