# Supplementary material for: Linkage mapping of root shape traits in two carrot populations
Source: G3 (Bethesda). 2024 Feb 27;14(4):jkae041. doi: 10.1093/g3journal/jkae041 (PMC10989876; doi:10.1093/g3journal/jkae041)
Supplement: jkae041_Supplementary_Data [file jkae041_supplementary_data.zip › Supplemental_Figures_G3-2023-404760.pdf]

Supplemental Figures

Andrey Vega, Scott H. Brainard, Irwin L. Goldman

Department of Plant and Agroecosystem Sciences, University of Wisconsin-Madison, Madison, Wisconsin, 53706, United States of America.

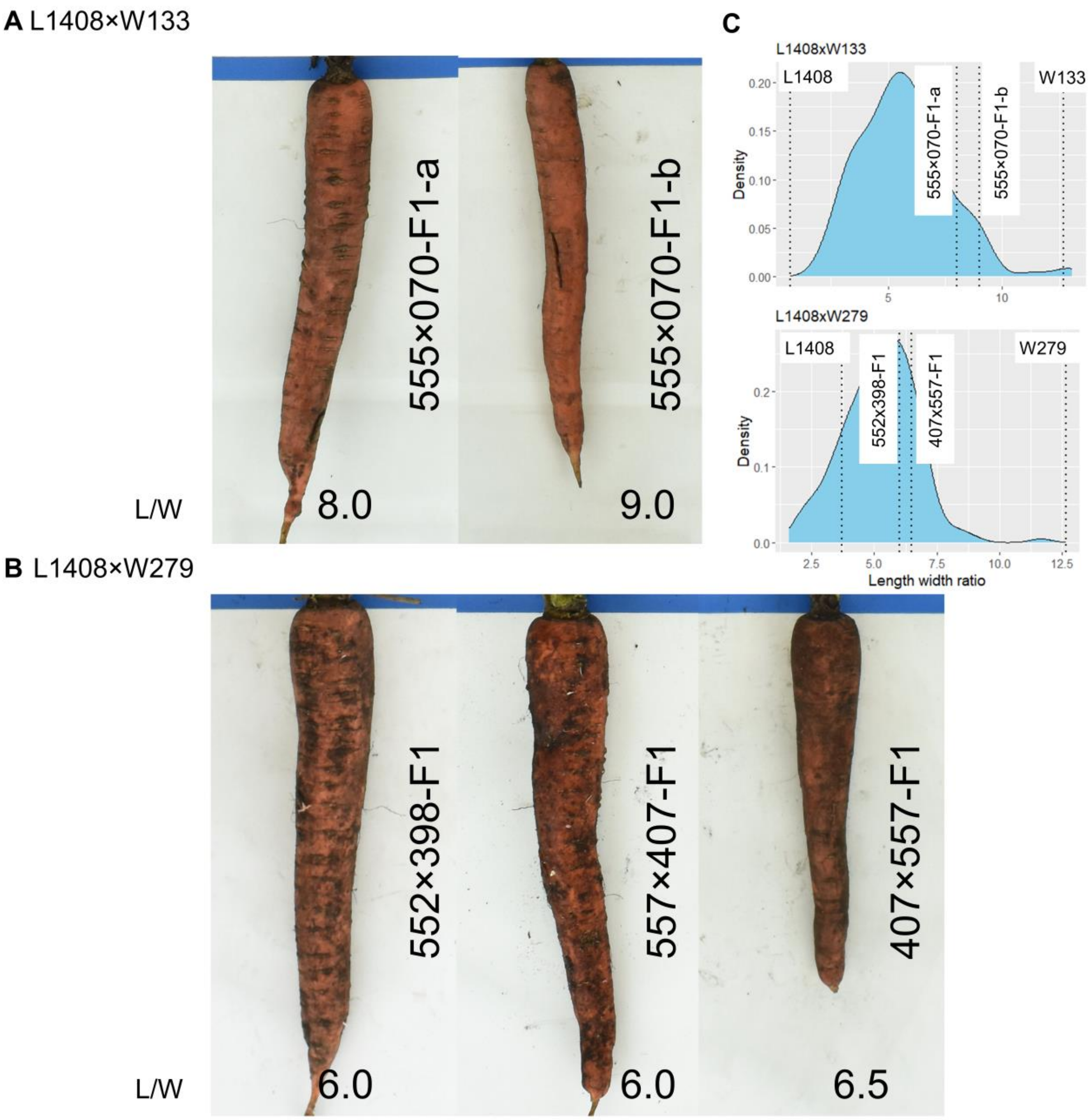

Figure S1 Scaled photographs illustrating the F$_1$ generation carrot roots of F$_{2:3}$ mapping populations: (**A**) L1408xW133 and (**B**) L1408xW279. Founder plants are denoted vertically: L1408 founder plants are 555, 552, and 557; W133 founder is 070; W279 founder plants are 398 and 407. (**C**) Phenotypic distributions of a population of 147 F$_1$ plants from the L1408xW133 cross and 189 F$_1$ plants from the L1408xW279 cross with the annotation of where the founders and F1 plants fell in the distribution. Ovule donor founder is shown before 'x' followed by the pollen donor. L/W represents length-to-width ratio score. In **A**, two F$_1$ roots from L1408xW133 cross (555x070-F1-A and 555x070-F1-B) were used to obtain F$_{2:3}$ progeny. In **B**, three F$_1$ roots from L1408xW279 cross (552x398-F1, 557x407-F1 and 407x557-F1) were used to obtain F$_{2:3}$ progeny.

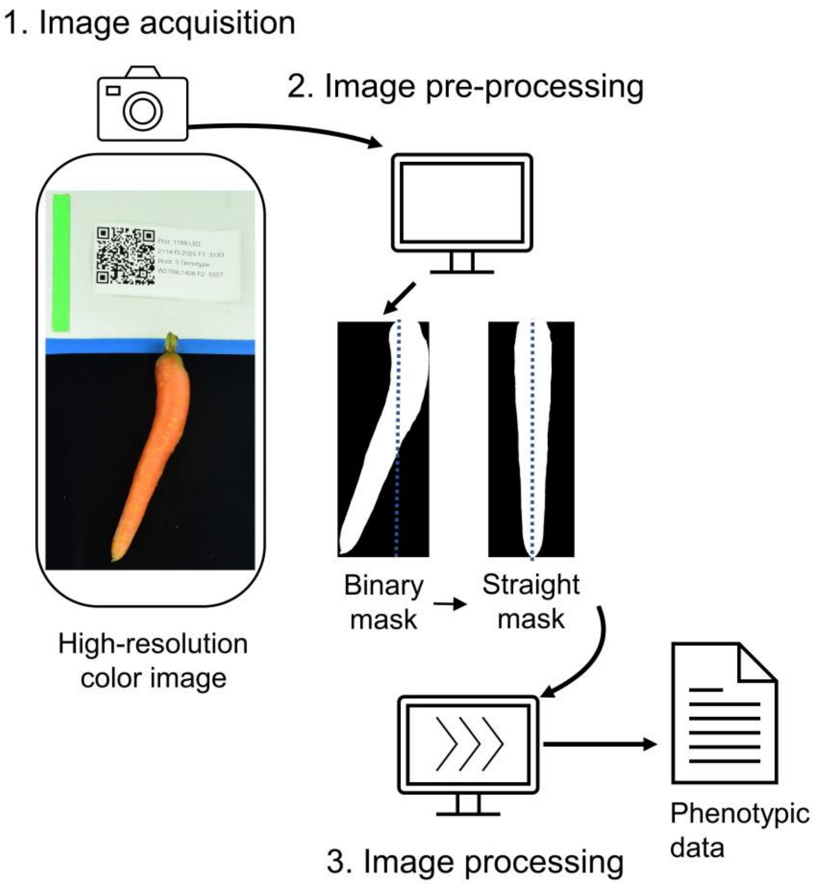

Figure S2 Digital imaging pipeline workflow using the digital imaging system for carrots by Brainard et al. (2021). Roots were cleaned and any forked or split roots were discarded. The remaining roots, along with a QR code containing genotype, unique identification, and year data, were placed in imaging templates. Image acquisition: high-resolution color images were acquired using a Nikon 5300 digital single-lens reflex camera connected to a computer running Smart Shooter (v.3.0). The images were saved with the information encoded in the QR code using carrot-phenotyping (https://github.com/shbrainard/carrot-phenotyping). Image pre-processing step: binary masks were created by distinguishing carrot pixels from the background in the high-resolution images. The binary masks were standardized by removing root tips and curving, resulting in straight masks. Image processing: phenotypes were computed on the straight masks using carrotsweeper (https://github.com/jbustamante35/carrotsweeper) and phenotypy.py from carrot-phenotyping.

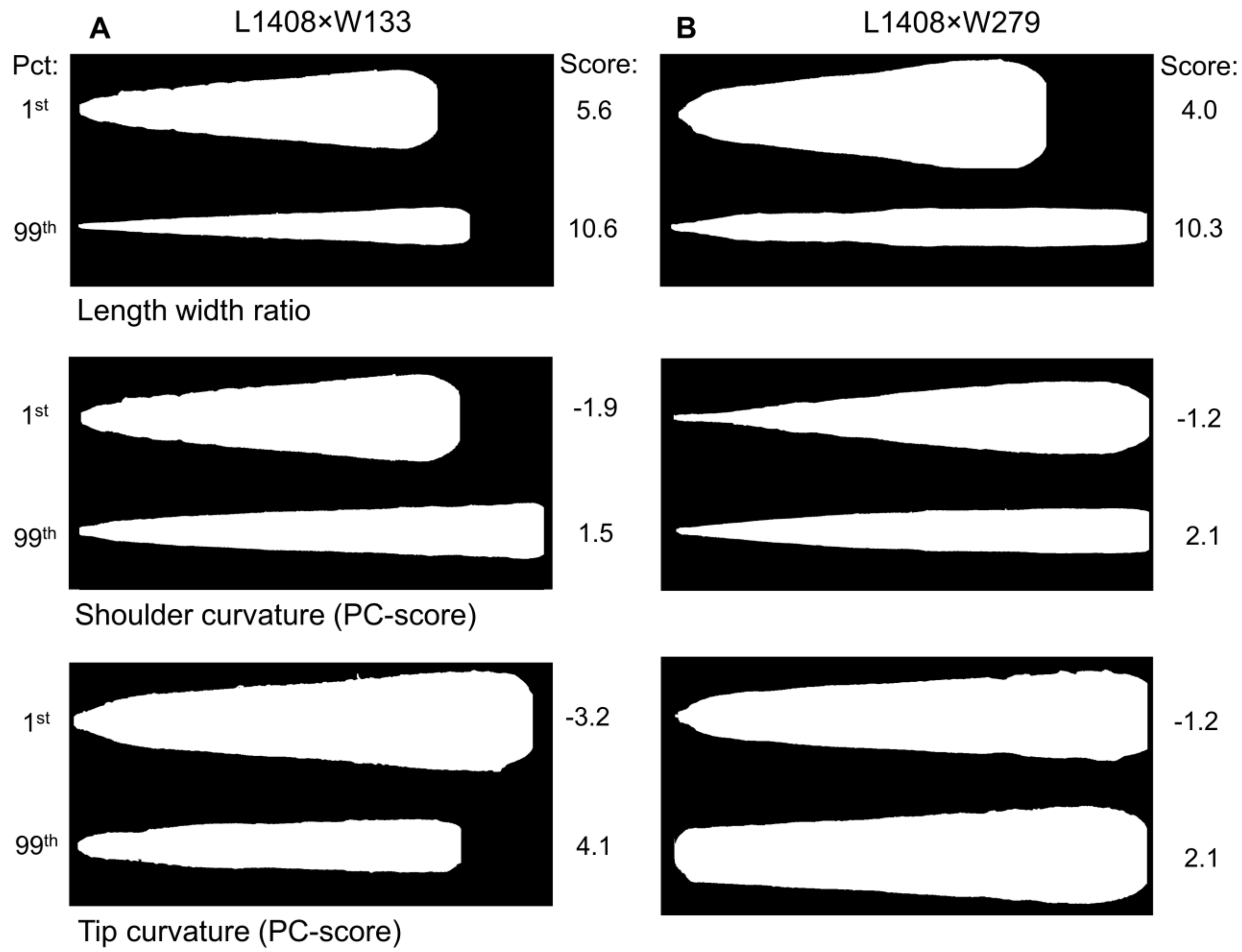

Figure S3 Schematic and quantitative score for tip curvature, shoulder curvature and length-to-width ratio phenotypes in carrot for $F_{2:3}$ mapping populations L1408×W133 (**A**) and L1408×W279 (**B**). Tip curvature and shoulder curvature were derived from Principal Component Analysis (PCA) of curvature values sampled at the first and last 50 pixels of the root contour respectively. Black and white pictures are straight masks of representative roots sampled from the 1-st and the 99-th percentiles (pct).

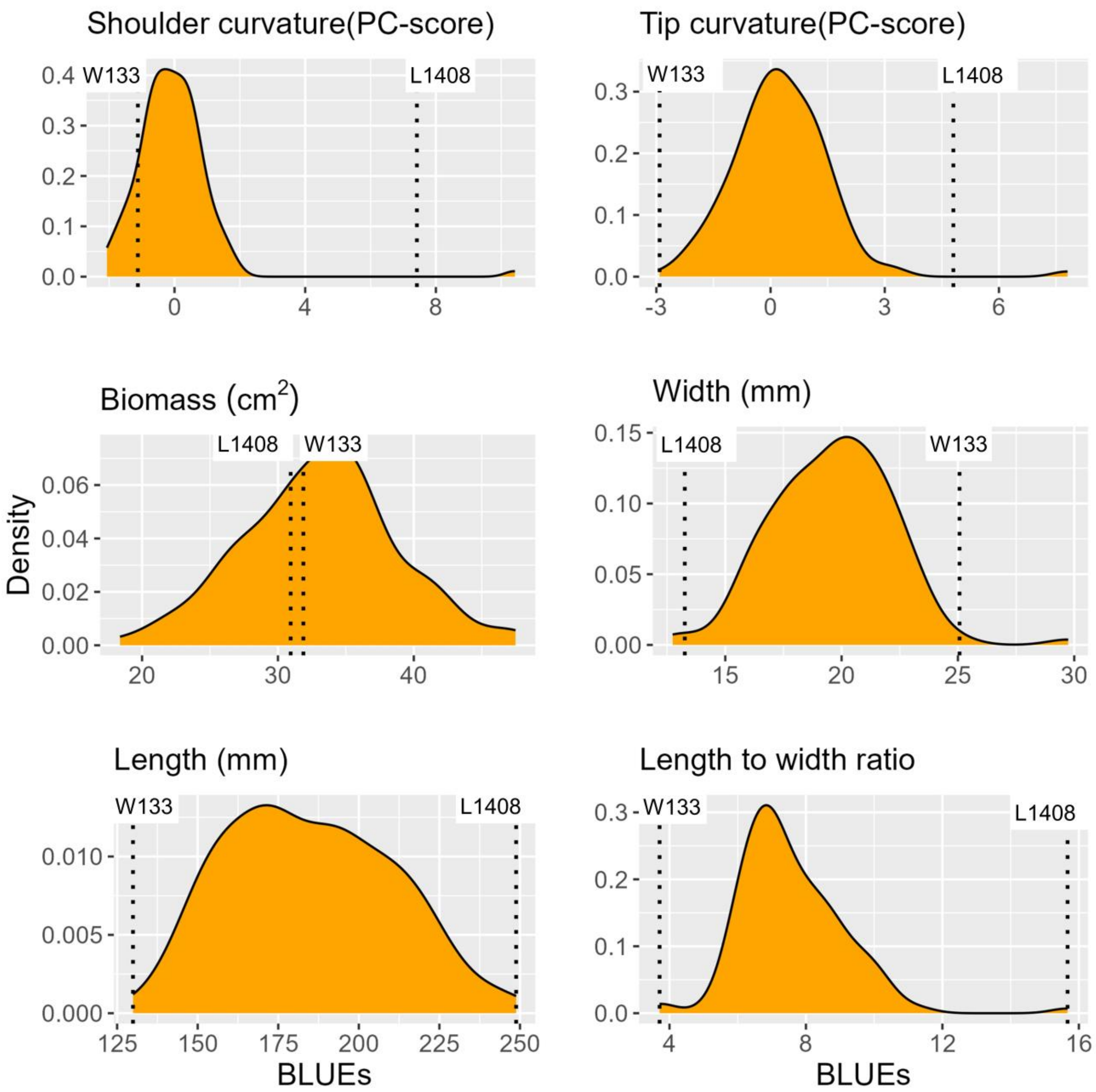

Figure S4 Density histogram for root shape traits for population L1408×W133. Vertical dotted lines indicate the position of the founders in each phenotypic variable. BLUEs=Best Linear Unbiased Estimator.

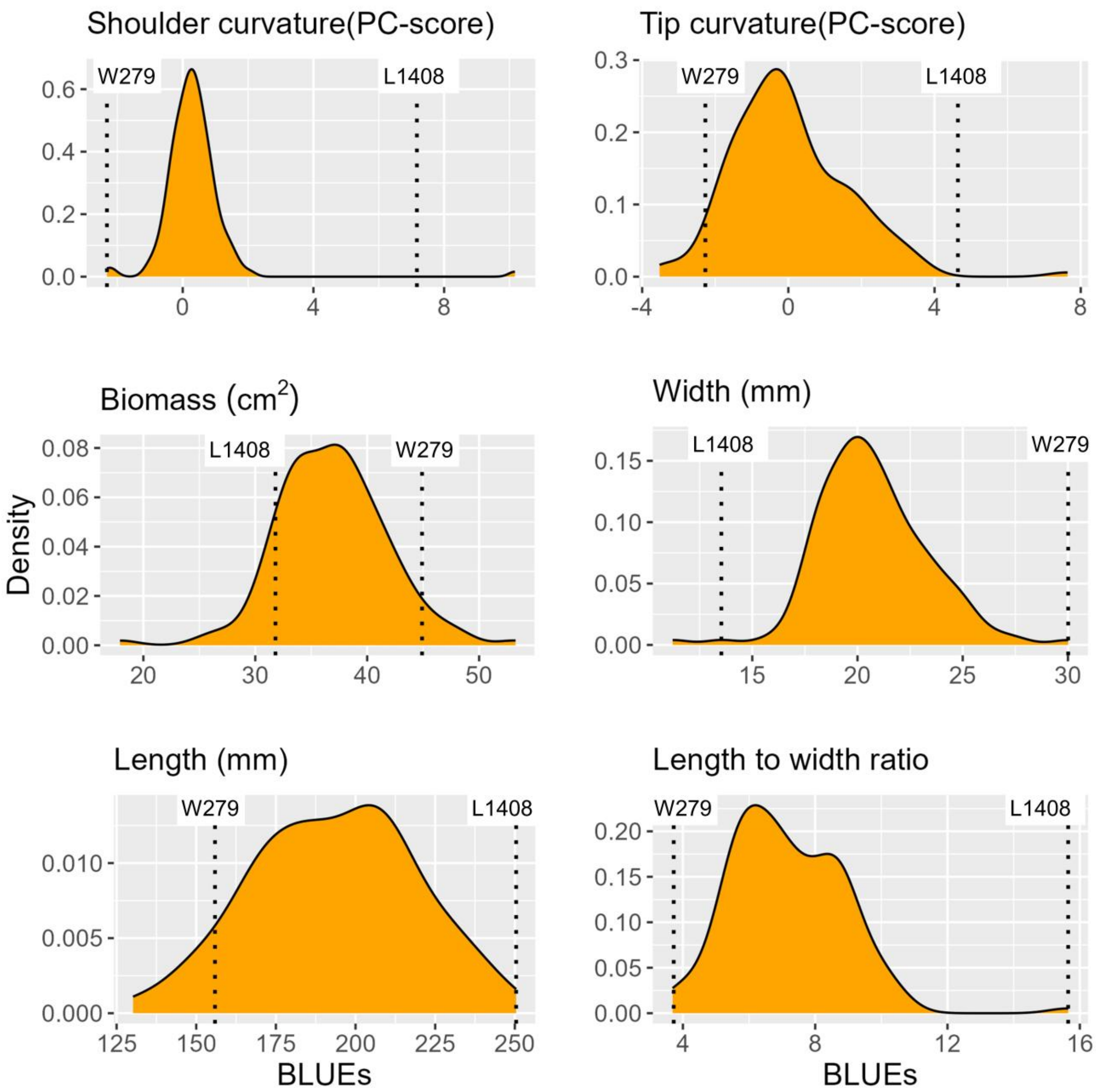

Figure S5 Density histogram for root shape traits for population L1408×W279. Vertical dotted lines indicate the position of the founders in each phenotypic variable. BLUEs=Best Linear Unbiased Estimator.

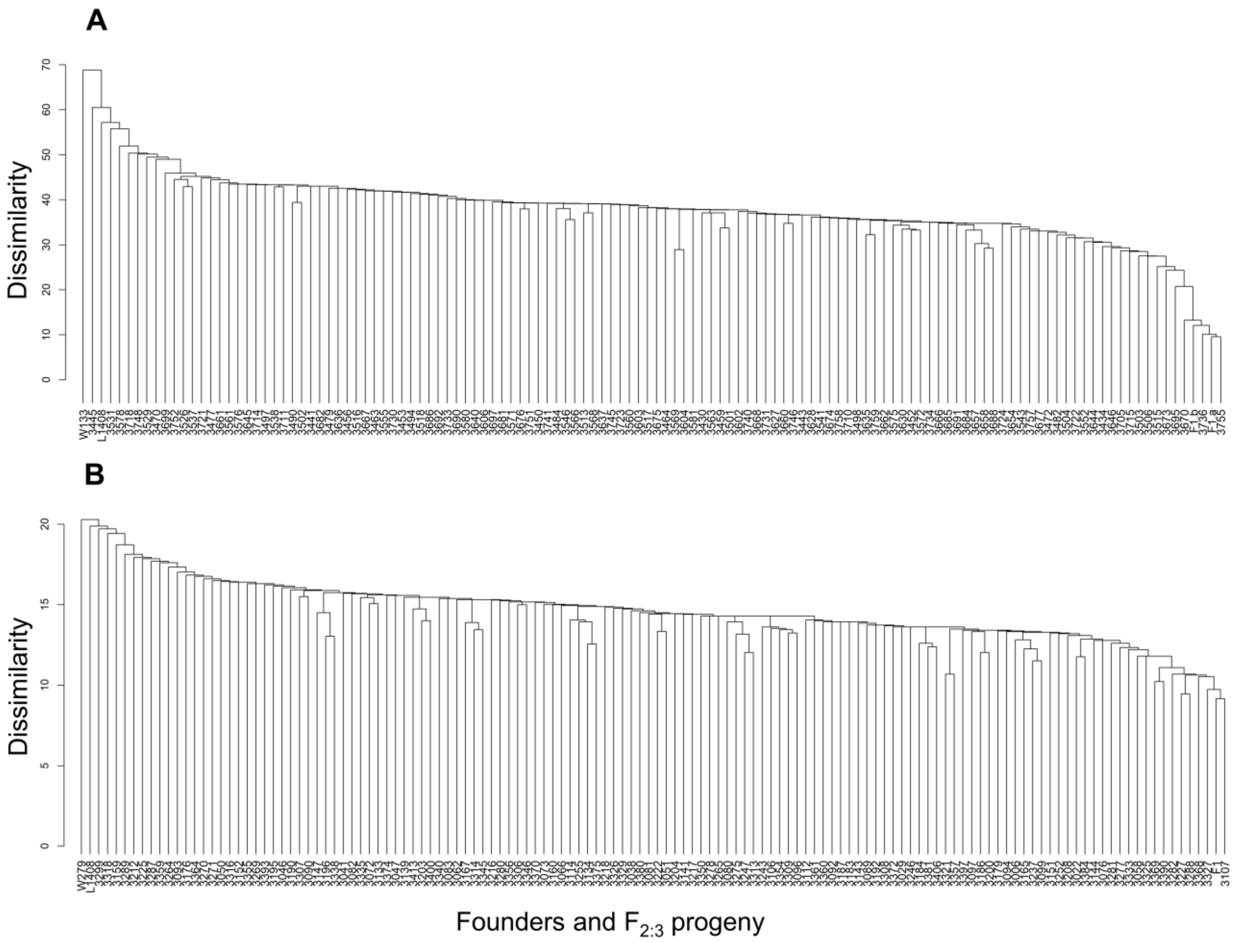

Figure S6 Agglomerative hierarchical clustering based on a dissimilarity matrix of SNP data for the L1408×W133 (**A**) and L1408×W279 (**B**) population. Clusters were generated by passing a dissimilarity matrix estimated using the dist function in R to the hclust function and using “single” as the argument for method. The dissimilarity between clusters (progeny) is represented by the height of the horizontal line segment connecting them. **A** Founders ‘L1408’ and ‘W133’ are the first to be included in the cluster and both F_1 individuals are the last. A total of 4,734 homozygous markers were used to estimate the dissimilarity matrix. **B** Founders ‘L1408’ and ‘W279’ are the first to be included in the cluster and the F_1 is one of the last. A total of 543 homozygous markers were used to estimate the dissimilarity matrix.

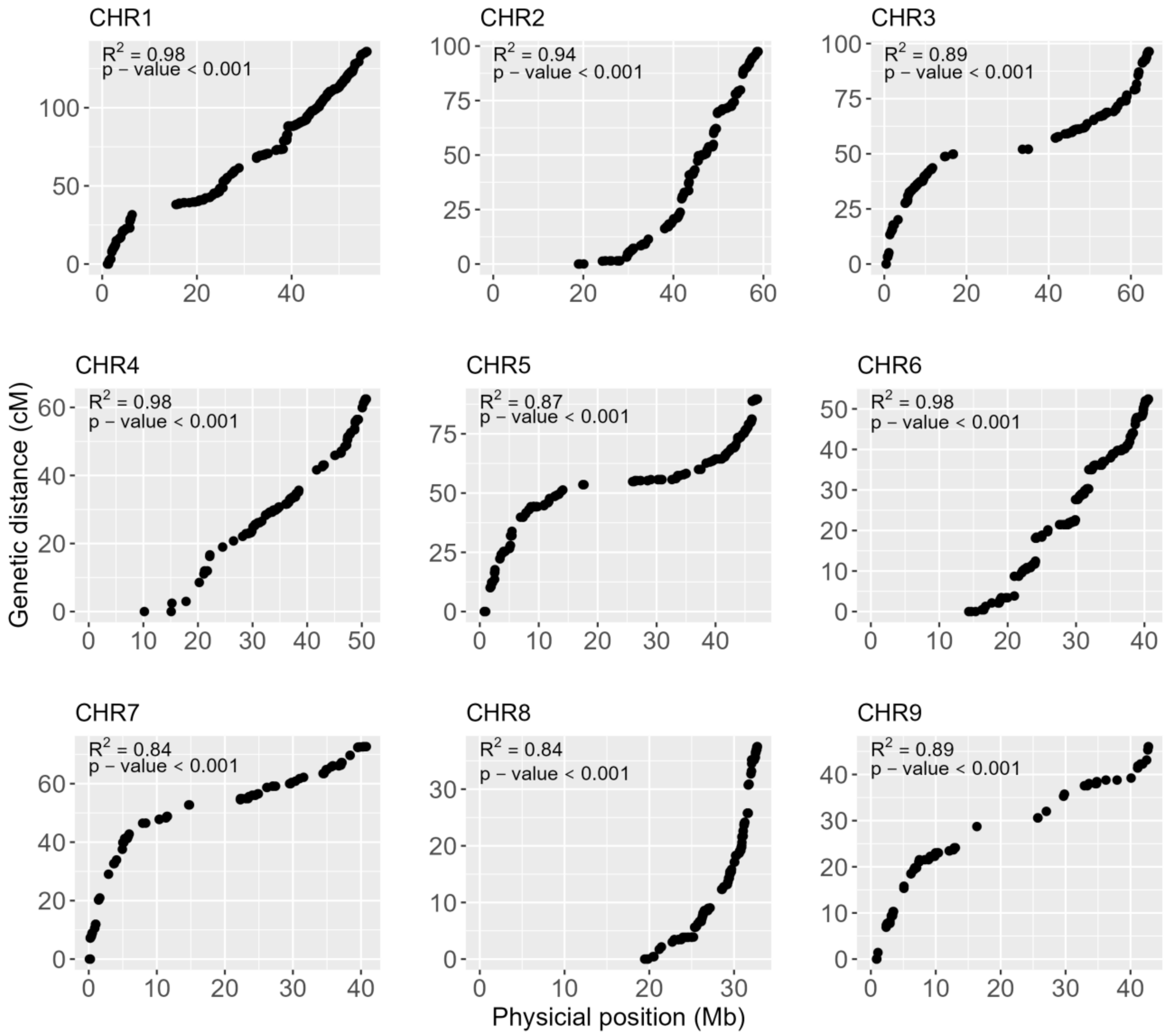

Figure S7 Comparison between version 3 of the carrot reference genome physical position (x-axis) and genetic distances (y-axis) estimated in the **L1408×W133** genetic map. Note that chromosomes 2,4,6 and 8 have no markers between 0 to 20 Mb and all chromosomes have low marker representation around the centromeres CHR=chromosomes. R^2 (adjusted coefficient of determination) and *P* value were obtained from the regression equation (Genetic distance ~ Physical position).

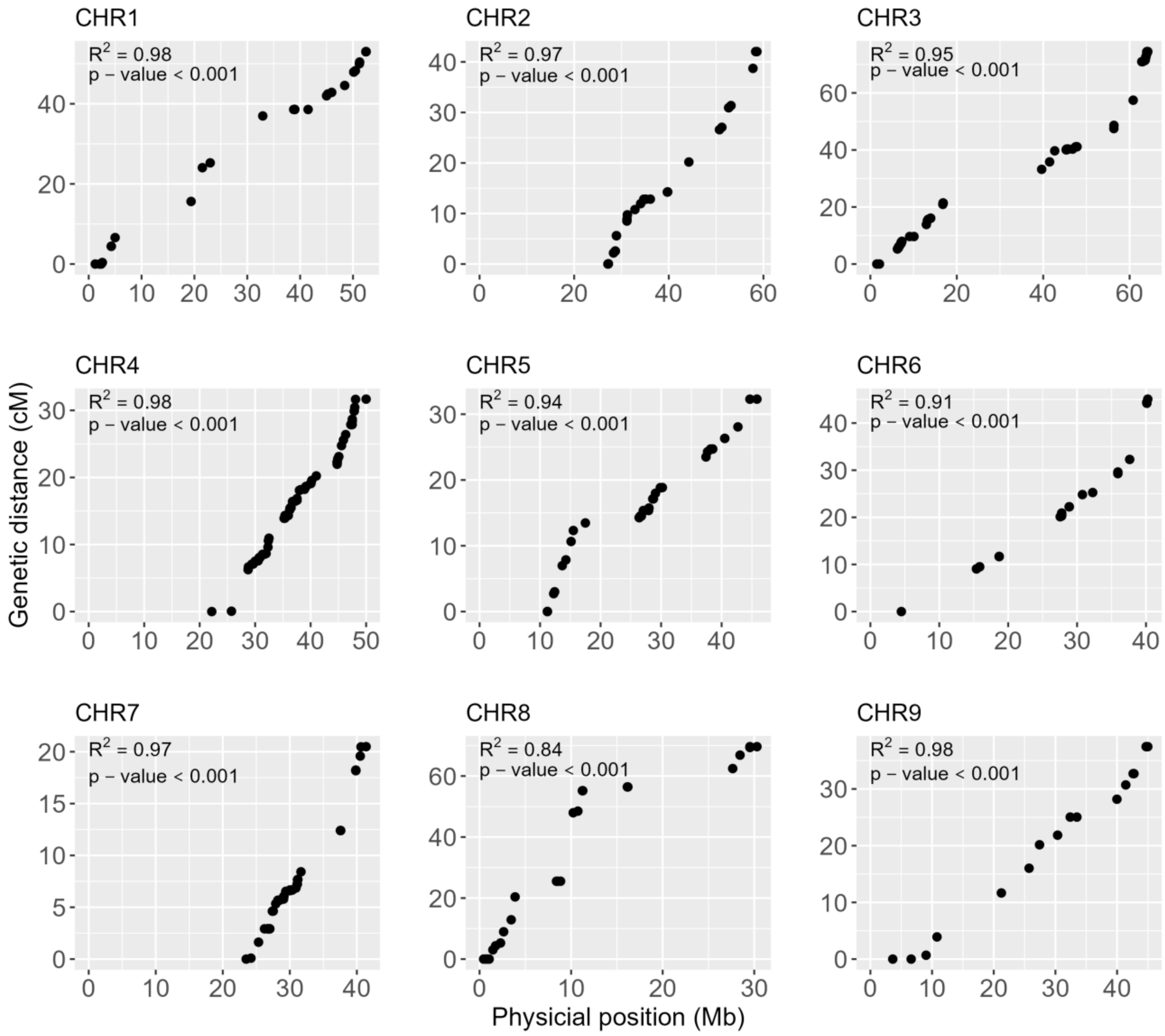

Figure S8 Comparison between version 3 of the carrot reference genome physical position (x-axis) and genetic distances (y-axis) estimated in the **L1408×W279** genetic map. Note that chromosomes 2,4,6 and 7 have no markers between 0 to 22 Mb and all chromosomes have low marker representation around the centromere. CHR=chromosomes. R^2 (adjusted coefficient of determination) and *P* value were obtained from the regression equation (Genetic distance ~ Physical position).

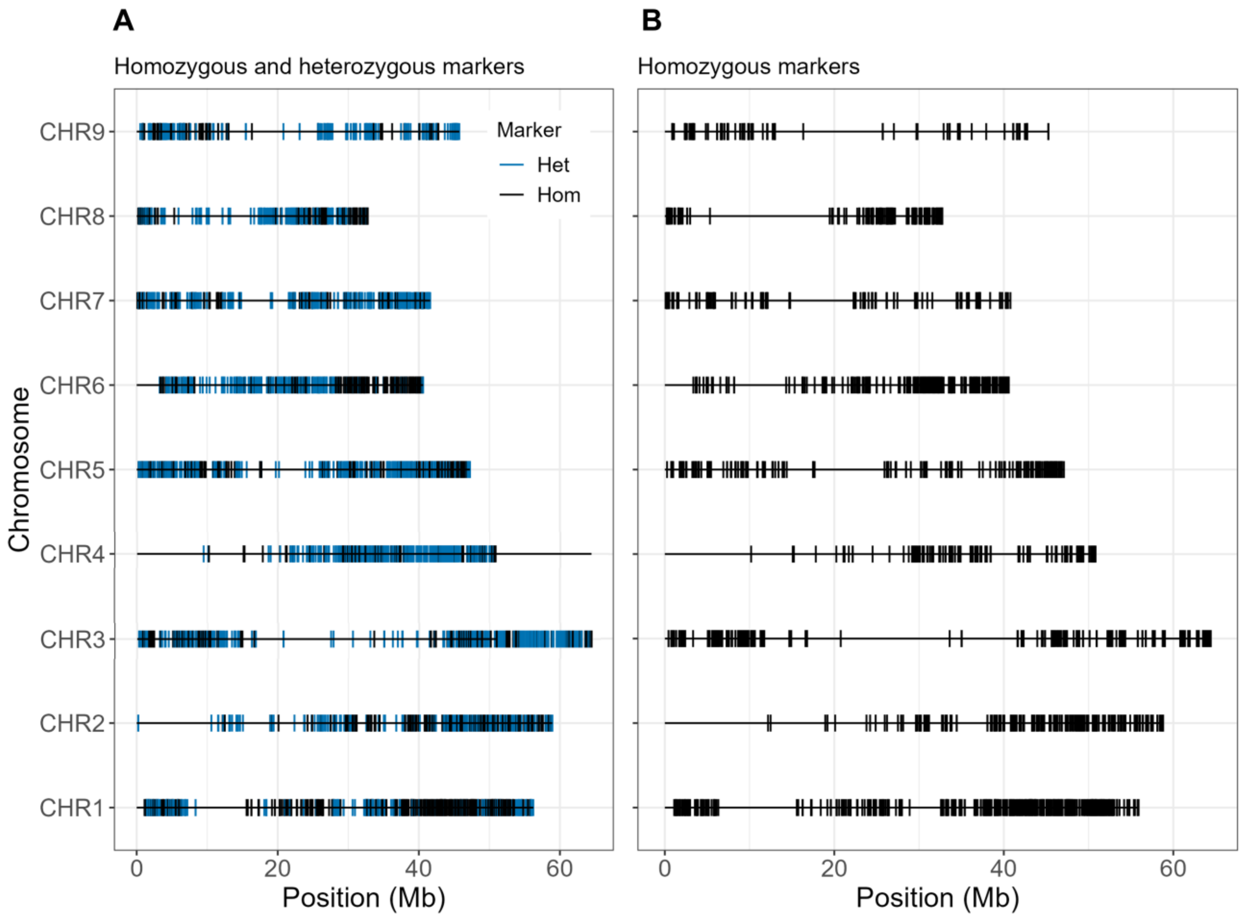

Figure S9 Coverage of SNP markers across the genome for population L1408×W133. **A** coverage of heterozygous and homozygous markers. **B** coverage of homozygous markers. Het=heterozygous, Hom=homozygous. Homozygous markers include only A×B or B×A markers and heterozygous include A×H, H×A, B×H, H×A markers (Braun et al. 2017). 'A' and 'B' denote the two homozygous states of founders ‘L1408’ and ‘W133’ respectively and 'H' the heterozygote state. Proportion of Hom markers= 42%.

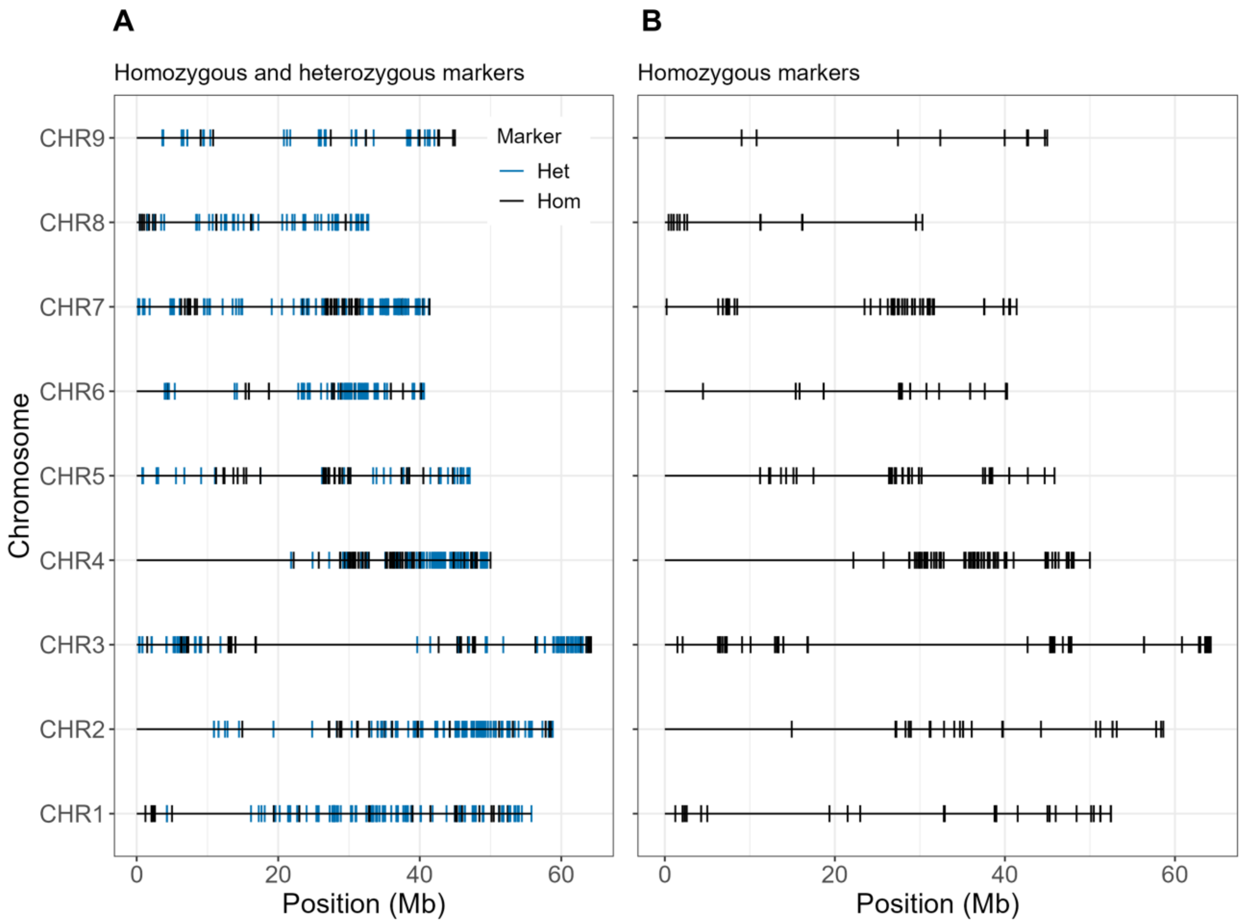

Figure S10 Coverage of SNP markers across the genome for population L1408×W279. **A** coverage provided by heterozygous and homozygous markers. **B** coverage of homozygous markers. Het=heterozygous, Hom=homozygous. Homozygous markers include only A×B or B×A markers and heterozygous include A×H, H×A, B×H, H×A markers (Braun et al. 2017). 'A' and 'B' denote the two homozygous states of founders ‘L1408’ and ‘W133’ respectively and 'H' the heterozygote state. Proportion of Hom markers= 31%.

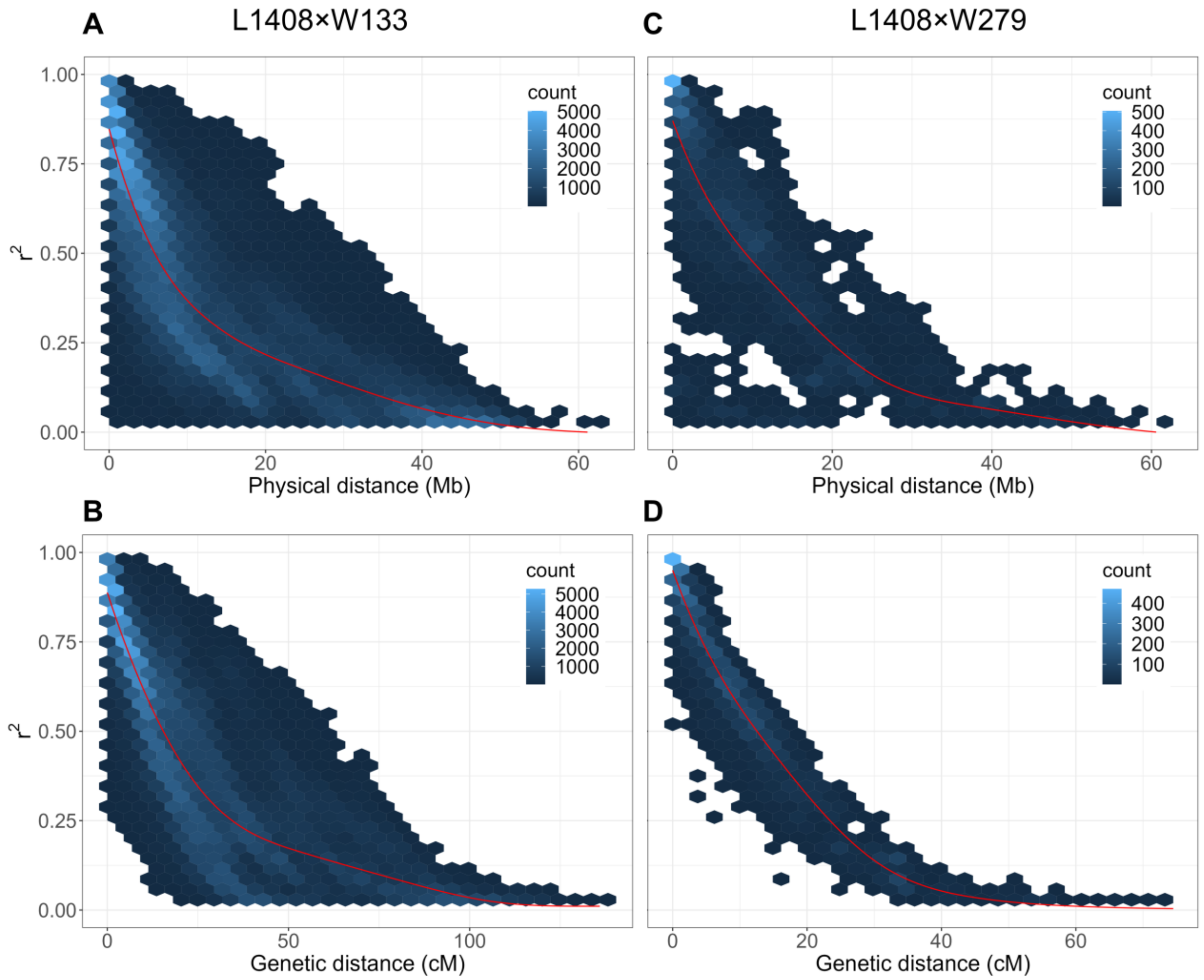

Figure S11 Genome-wide LD decay in two carrot $F_{2:3}$ mapping populations. Average LD decay plotted against physical distance (**A**) and genetic distance (**B**) in population L1408×W133. A value of r^2 = 0.15 intersects physical distance at 28 Mb and genetic distance at 58 cM. Average LD decay plotted against physical distance (**C**) and genetic distance (**D**) in population L1408×W279. A value of r^2 = 0.15 intersects physical distance at 26 Mb and genetic distance at 29 cM.

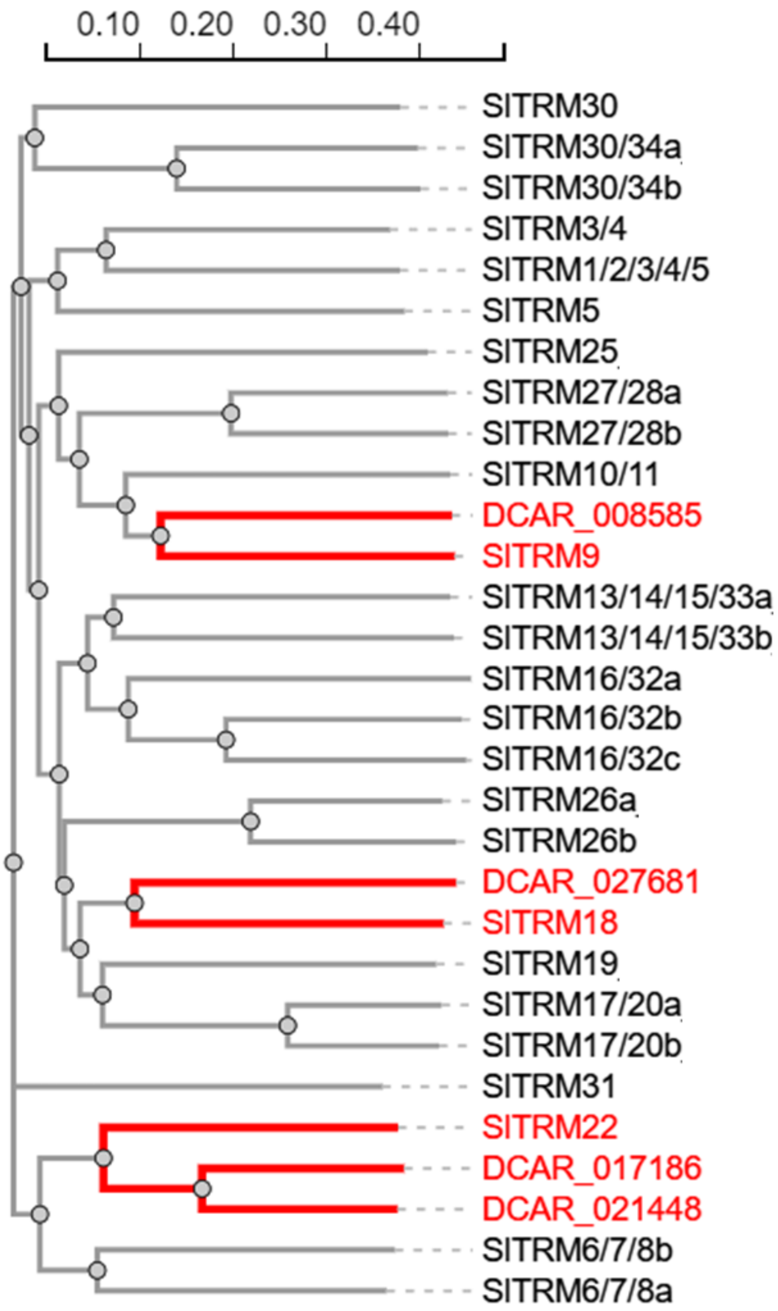

Figure S12 Visual representation of the relationships among gene sequences of carrot and tomato *TRMs* homologs. Branches represent the relatedness of sequences based on alignments. Length of the branches represents degree of genetic divergence. The closer the branches or sequences are to each other, the more closely related they are. In red the carrot gene sequence with its closest tomato homolog.
