# Supplementary material for: Linkage mapping of root shape traits in two carrot populations
Source: G3 (Bethesda). 2024 Feb 27;14(4):jkae041. doi: 10.1093/g3journal/jkae041 (PMC10989876; doi:10.1093/g3journal/jkae041)
Supplement: jkae041_Supplementary_Data [file jkae041_supplementary_data.zip › Supplemental_Tables_G3-2023-404760.pdf]

## Supplemental Tables

Andrey Vega, Scott H. Brainard, Irwin L. Goldman

Department of Plant and Agroecosystem Sciences, University of Wisconsin-Madison, Madison, Wisconsin, 53706, United States of America.

**Table S1**. Residual heterozygosity in the founders and $F_1$ plants of carrot mapping populations L1408×W133 and L1408×W279.

| ID | Founder | Homozygous genotype frequency (AA or BB) | Heterozygous genotype frequency (AB) |
|---|---|---|---|
| | | *L1408×W133* | |
| 555 | L1408 | 0.76 | 0.24 |
| 070 | W133 | 0.55 | 0.45 |
| 555×070-a | F1-a | 0.32 | 0.68 |
| 555×070-b | F1-b | 0.37 | 0.63 |
| | | *L1408×W279* | |
| 552 | L1408 | 0.71 | 0.29 |
| 557 | L1408 | 0.44 | 0.66 |
| 398 | W279 | 0.66 | 0.34 |
| 407 | W279 | 0.58 | 0.42 |
| 552×398 | F1-a | 0.42 | 0.58 |
| 557×407 | F1-b | 0.38 | 0.62 |
| 407×557 | F1-c | 0.29 | 0.71 |

**Table S2.** Number of markers and maximum spacing for linkage map of chromosomes 3, 8 and 9 of population L1408×W279 constructed using either only homozygous or homozygous + heterozygous. Marker types include homozygous (A×B, B×A), and heterozygous (A×H, H×B, B×H and H×B) markers. A= Homozygous marker for L1408, B= Homozygous marker for W279, and H= Heterozygous marker.

| Chromosome | Homozygous | | Homozygous and Heterozygous | | Number of additional Heterozygous markers | Gap reduction (cM) |
|---|---|---|---|---|---|---|
| | Number of markers | Maximum spacing (cM) | Number of markers | Maximum spacing (cM) | | |
| 3 | 60 | 19.0 | 62 | 13.5 | 2 | 5.5 |
| 8 | 15 | 44.7 | 24 | 22.5 | 9 | 22.2 |
| 9 | 9 | 20.4 | 16 | 7.8 | 7 | 12.6 |

**Table S3**. Loci significantly associated with root shape traits identified in carrot $F_{2:3}$ mapping populations L1408×W133 (n=119) and L1408×W279 × (n=128). In bold, two QTLs recovered in both populations with an overlapping 1.5 LOD interval (reproducible QTLs).

| Trait | Locus | Chr[1] | Map pos[2] (cM) | Phys pos[3] (bp) | LOD | %var exp[4] | 1.5 LOD interval[5] (bp) | Dom degree [6] | Allele substitution effects[7] | |
|---|---|---|---|---|---|---|---|---|---|---|
| | | | | | | | | | $\alpha$ | δ |
| | | | | *Population L1408×W133* | | | | | | |
| Shoulder[1] | c.5loc.45 | 5 | 45 | 10886001 | 6.2 | 22 | 7348535-13412756 | 0.5 | -0.7 | 0.3 |
| Tip[1] | c.3loc.79 | 3 | 79 | 61018994 | 4.5 | 16 | 11742006-61751069 | 1.0 | -0.5 | 0.5 |
| Biomass | c.8loc.2 | 8 | 2 | 21431131 | 3.8 | 14 | 19453332-32696614 | 0.2 | -343 | 80 |
| Width | c.3loc.53 | 3 | 53 | 35021817 | 4.3 | 16 | 11742006-63711032 | 19 | 0.1 | 1.9 |
| **Width** | **c.6loc.50** | **6** | **50** | **39847186** | **4.6** | **17** | **14319870-40582356** | **0.7** | **1.1** | **0.8** |
| **L/W[1]** | **c.2loc.19** | **2** | **19** | **39653470** | **5.7** | **20** | **32851615-54149218** | **0.3** | **-0.9** | **-0.3** |
| L/W | c.3loc.56 | 3 | 56 | 41614217 | 4.7 | 17 | 11516803-61378990 | 1.0 | -0.6 | -0.6 |
| L/W | c.6loc.50 | 6 | 50 | 39875002 | 4.3 | 16 | 14319870-40582356 | 0.1 | -0.7 | -0.1 |
| L/W | c.8loc.8 | 8 | 0 | 19453332 | 5.7 | 20 | 19453332-26176663 | 0.6 | -0.8 | -0.4 |
| Length | c.6loc.6 | 6 | 18 | 24151730 | 3.8 | 14 | 14319870-29921627 | 1 | -12 | 12 |
| Length | c.8loc.0 | 8 | 0 | 20539941 | 7.9 | 27 | 19453332-25702759 | 0.1 | -19 | -2.4 |
| | | | | *Population L1408×W279* | | | | | | |
| Shoulder | c.2loc.18 | 2 | 18 | 44243993 | 6.1 | 20 | 32846084-5070617 | 0.2 | -0.5 | -0.1 |
| **Width[8]** | **c.6loc.45** | **6** | **45** | **40259670** | **5.0** | **16** | **37630119-40259670** | **0.3** | **1.5** | **0.5** |
| **L/W** | **c.2loc.34** | **2** | **34** | **53149932** | **4.3** | **14** | **39769213-58630847** | **0.3** | **-0.9** | **-0.3** |
| Length | c.2loc.37 | 2 | 37 | 57786982 | 3.3 | 11 | 39769213-58630847 | 1 | -12 | 12 |

[1] Chr=Chromosome, Tip=tip curvature, Shoulder= shoulder curvature. L/W= length-to-width ratio.
[2] Genetic distances in centimorgans (cM) estimated from linkage maps constructed from $F_{2:3}$ carrot mapping populations L1408×W133 and L1408×W133.
[3] Physical position (bp) according to version 3 of the carrot reference genome of the most significant SNP.
[4] Percent phenotypic variance explained by the QTL estimated as $1 - 10^{-2LOD/n}$ where LOD is LOD score, and n is the number of individuals.
[5] Support interval for the QTL location in bp.
[6] Dominance degree, defined as the absolute value of the ratio of dominance to additive substitution effects: $\left|\frac{\delta}{\alpha}\right|$.
[7] $\alpha$ is the additive substitution effect defined as $\alpha = \frac{1}{2}(g_B - g_A)$. $\delta$ is the dominance substitution effect defined as $\delta = g_H - \frac{g_B + g_A}{2}$ where $g_B$ and $g_A$ are phenotype means for the two homozygote states and $g_H$ is the phenotype mean fort the heterozygote expressed in the units corresponding to each trait.

**Table S4**. List of homologous genes in the OFP-TRM and IQD regulon within 1.5 LOD interval of significant QTLs. In bold are candidate genes 2 Mb or less from predicted genes controlling biological shape.

| Trait | SNP[1] (chrom:pos) | Gene ID | Locus tag | Gene position (bp) | Dist[2] (Mb) | Description[3] |
|---|---|---|---|---|---|---|
| L/W | SDCARV3_CHR2:39653470 | **LOC108210025** | **DCAR_007928** | **38299380-38300937** | **1.4** | **Transcription repressor OFP5** |
| | | LOC108206931 | DCAR_007743 | 36783814-36787044 | 2.9 | Domain of unknown function. |
| | | LOC108208046 | DCAR_008585 | 43315793-43317575 | 3.7 | Uncharacterized (TRM9) |
| | SDCARV3_CHR6: 39875002 | LOC108224231 | DCAR_020875 | 27642280-27646934 | 12.2 | Predicted protein IQD 1 |
| | | LOC108225605 | DCAR_020362 | 32009199-32011469 | 7.8 | Protein IQ-DOMAIN 1-like |
| | | LOC108226575 | DCAR_020520 | 30661816-30664209 | 9.2 | Protein IQ-DOMAIN 14-like |
| | | LOC108226810 | DCAR_020721 | 28906376-28907372 | 10.9 | Transcription repressor OFP6 |
| | SDCARV3_CHR3:41614217 | **LOC108213599** | **DCAR_012074** | **43525575-43526563** | **1.9** | **Transcription repressor OFP8** |
| | SDCARV3_CHR8:19453332 | **LOC108200088** | **DCAR_027681** | **20635477-20641073** | **1.2** | **Uncharacterized (TRM18)** |
| | SDCARV3_CHR2:53149932 | LOC108206091 | DCAR_008398 | 41828348-41832564 | 11.3 | Uncharacterized |
| | | LOC108208046 | DCAR_008585 | 43315793-43317575 | 9.8 | Uncharacterized |
| Width | SDCARV3_CHR6:39847186 | LOC108225605 | DCAR_020362 | 32009199-32011469 | 7.8 | Protein IQ-DOMAIN 1-like |
| | | LOC108225303 | DCAR_020324 | 32328760-32332301 | 7.8 | Protein IQ-DOMAIN 1-like |
| | | LOC108226562 | DCAR_020722 | 22863700-22868859 | 17 | Transcription repressor OFP13 |
| | SDCARV3_CHR3:35021817 | **LOC108214575** | **DCAR_011147** | **33004221-33012820** | **2.0** | **Protein IQ-DOMAIN 31-like** |
| | | LOC108214737 | DCAR_009937 | 12710628-12715941 | 22.3 | protein IQ-DOMAIN 32-like |
| | | LOC108213599 | DCAR_012074 | 43525575-43526563 | 8.5 | transcription repressor OFP8-like |
| | | LOC108213408 | DCAR_012488 | 47514286-47515260 | 12.5 | transcription repressor OFP12-like |
| | | LOC108211116 | DCAR_012508 | 47684073-47688137 | 12.6 | Protein LONGIFOLIA 1 |
| | SDCARV3_CHR6:40259670 | *No OPFs, TRMs or IQDs found within the 1.5 LOD support interval* | | | | |
| Length | SDCARV3_CHR8:20539941 | LOC108197110 | DCAR_028703 | 2501780-2507107 | 4.5 | Uncharacterized |
| | | **LOC108199732** | **DCAR_027520** | **22295708-22300142** | **1.7** | **Protein IQ-DOMAIN 14** |
| | | LOC108197768 | DCAR_027238 | 24917458-24919729 | 6.7 | Protein IQ-DOMAIN 14-like |

| | | | | | | |
|---|---|---|---|---|---|---|
| | | LOC108200088 | DCAR_027681 | 20635477-20641073 | 7.1 | Uncharacterized |
| | SDCARV3_CHR2:57786982 | LOC108208046 | DCAR_008585 | 43315793-43317575 | 14.4 | Uncharacterized |
| | SDCARV3_CHR6:24151730 | LOC108224231 | DCAR_020875 | 27642280-27646934 | 3.5 | Predicted protein IQD 1 |
| | | LOC108226810 | DCAR_020721 | 28906376-28907372 | 4.8 | Transcription repressor OFP6 |
| | | **LOC108226562** | **DCAR_020722** | **22863700-22868859** | **1.3** | **Transcription repressor OFP13** |
| | | **LOC108228003** | **DCAR_021448** | **22863700-22868859** | **1.3** | **Uncharacterized (TRM22)** |
| | | LOC108224245 | DCAR_020774 | 28467273-28472568 | 4.3 | Uncharacterized |
| Shoulder | SDCARV3_CHR5:10886001 | **LOC108219819** | **-** | **9853096-9855397** | **1.0** | **Protein IQ-DOMAIN 14-like** |
| | | LOC108221137 | DCAR_016221 | 1326651-1327597 | 2.4 | transcription repressor OFP14 |
| | | **LOC108220104** | **DCAR_017186** | **12549724-12554368** | **1.7** | **Uncharacterized (TRM22)** |
| | SDCARV3_CHR2:44243993 | LOC108206825 | DCAR_007973 | 38613160-38615754 | 5.6 | Protein IQ-DOMAIN 1-like |
| | | LOC108206589 | DCAR_007588 | 35586356-35589381 | 8.6 | Protein IQ-DOMAIN 14 |
| | | LOC108210025 | DCAR_007928 | 38299380-38300937 | 6.0 | transcription repressor OFP5 |
| | | LOC108208046 | **DCAR_008585** | **43315793-43317575** | **0.9** | **Uncharacterized (TRM9)** |
| Tip | SDCARV3_CHR3:61018994 | LOC108214575 | DCAR_011147 | 33004221-33012820 | 28 | Protein IQ-DOMAIN 31-like |
| | | LOC108214737 | DCAR_009937 | 12710628-12715941 | 48.3 | protein IQ-DOMAIN 32-like |
| | | LOC108213599 | DCAR_012074 | 43525575-43526563 | 17.5 | Transcription repressor OFP8-like |
| | | LOC108213292 | DCAR_010451 | 23239336-23240569 | 37.8 | transcription repressor OFP8-like |
| | | LOC108213408 | DCAR_012488 | 47514286-47515260 | 13.5 | transcription repressor OFP12-like |
| | | LOC108210824 | DCAR_012067 | 43481190-43485847 | 17.6 | Uncharacterized |
| | | LOC108211116 | DCAR_012508 | 47684073-47688137 | 13.4 | Protein LONGIFOLIA 1 |
| Biomass | SDCARV3_CHR8:21431131 | **LOC108199732** | **DCAR_027520** | **22295708-22300142** | **0.8** | **Protein IQ-DOMAIN 14** |
| | | LOC108197768 | DCAR_027238 | 24917458-24919729 | 3.5 | protein IQ-DOMAIN 14-like |
| | | **LOC108200088** | **DCAR_027681** | **20635477-20641073** | **0.8** | **Uncharacterized** |
| | | LOC108197110 | DCAR_028703 | 2501780-2507107 | 3.6 | Uncharacterized |

[1] Single Nucleotide Polymorphism (SNP) in the column refers to the most significant SNP (highest LOD score) chosen to represent the QTL region. Variants were aligned to version 3 of the reference genome (Coe et al. 2023).
[2] Physical distance (Mb) between the start of the candidate gene position and the SNP at a significant QTL. Distance is based on the reported physical distance of candidate genes on the assembly GCA_001625215.1, bioproject PRJNA268187.
[3] Description. Some candidate genes are labeled as uncharacterized in the NCBI BLAST datasets as they lack functional or predicted annotation. Two TRM conserved motifs were aligned to four previously uncharacterized predicted carrot genes DCAR_008585 (LOC108208046), DCAR_017186 (LOC108220104), DCAR_021448 (LOC108228003) and DCAR_027681 (LOC108200088). All genes are within the 1.5 LOD support interval of QTL peaks controlling length, width and length-to-width ratio in chromosomes 2,5,6 and 8 (See supplementary material 3).
